# Structural Aspects of the Superionic Transition in AX_2_ Compounds With the Fluorite Structure

**DOI:** 10.3389/fchem.2021.723507

**Published:** 2021-10-18

**Authors:** Paul C. M. Fossati, Alain Chartier, Alexandre Boulle

**Affiliations:** ^1^ DES–Service de Corrosion et du Comportement des Matériaux dans leur Environnement (SCCME), CEA Saclay, Université Paris Saclay, Gif-sur-Yvette, France; ^2^ Institut de Recherche sur les Céramiques (IRCer), CNRS UMR 7315, Université de Limoges, Centre Européen de la Céramique, Limoges, France

**Keywords:** molecular dynamics, fluorite, superionic, atomic-scale simulation, computational diffraction, XRD

## Abstract

Some *AX*_2_ binary compounds with the fluorite structure (space group $Fm\bar{3}m$ ) are well-known examples of materials exhibiting transitions to ionic superconducting phases at high temperatures below their melting points. Such superionic states have been described as either highly defective crystals or part-crystal, part-liquid states where the *A* ions retain their crystalline order whilst the *X* ions undergo partial melting. However, no detailed description of the structure of these phases exists. We present here the results of our investigation of the structural changes that occur during these transitions and the structural characteristics of the resulting superionic materials. This work is based on atomic-scale molecular dynamics modelling methods as well as computational diffraction techniques. We employed a set of empirical potentials representing several compounds with the fluorite structure to investigate any potential-dependent effect. We show the importance of small-scale structure changes, with some local environments showing a hexagonal symmetry similar to what is seen in the scrutinyite structure that has been documented for example in UO_2_.

## 1 Introduction

Superionic conductors are ionic solids that exhibit a very high ionic conductivity, close to that of their melt or comparable to that of a liquid electrolyte (Rice and Roth, 1972; Boyce and Huberman, 1979). The first materials undergoing a transition to a superionic state were documented in the 19th century (Faraday, 1849). Such a transition is now known in several compounds with the fluorite structure (Derrington et al., 1975). In the literature, this is referred to as the Faraday transition, Bredig transition, or diffuse transition because the change occurs over a finite temperature range instead of a single temperature. It corresponds to a second-order or class III superionic transition (O’Keeffe, 1976; Hull, 2004). Common examples are fluorides such as CaF_2_, SrF_2_, BaF_2_, or *β*-PbF_2_ (Lidiard, 1980), but also chlorides such as SrCl_2_ (Carr et al., 1978; Dixon, 1980; Gillan and Dixon, 1980; Fomin, 2021) or *β*-BaCl_2_ (Hull et al., 2011), or oxides such as Li_2_O (Fracchia et al., 1998; Goel et al., 2004), CeO_2_ (Klarbring et al., 2018), or UO_2_ (Hiernaut et al., 1993). It has also been demonstrated by simulations in more complex compounds such as actinides mixed oxides (U,Pu)O_2_ (Cooper et al., 2015; Takoukam-Takoundjou et al., 2020; Bathellier et al., 2021) (U,Th)O_2_ (Cooper et al., 2014a), or (Pu,Th)O_2_ (Galvin et al., 2016).

Besides the increased ionic conductivity, a known feature of the superionic transition is a peak in the constant-volume heat capacity *C*_P_(*T*) with a characteristic Λ shape (Goff et al., 1991). The cause for this is an increase in the enthalpy *H*(*T*) beyond what would be expected from the trend measured from the same materials at low temperatures (Naylor, 1945; Dworkin and Bredig (1963, 1968)). This has been commonly referred to as anomalous heat (Dworkin and Bredig, 1968; Szwarc, 1969). Coincidently, an anomalous increase in the lattice parameter of the materials can also be measured, or equivalently a Λ peak in the linear thermal expansion coefficient *α*(*T*) (Goff et al., 1991). Both peaks have their highest point at the same temperature for any given compound, which is conventionally taken as the superionic transition temperature *T*_S_.

**FIGURE 1:**
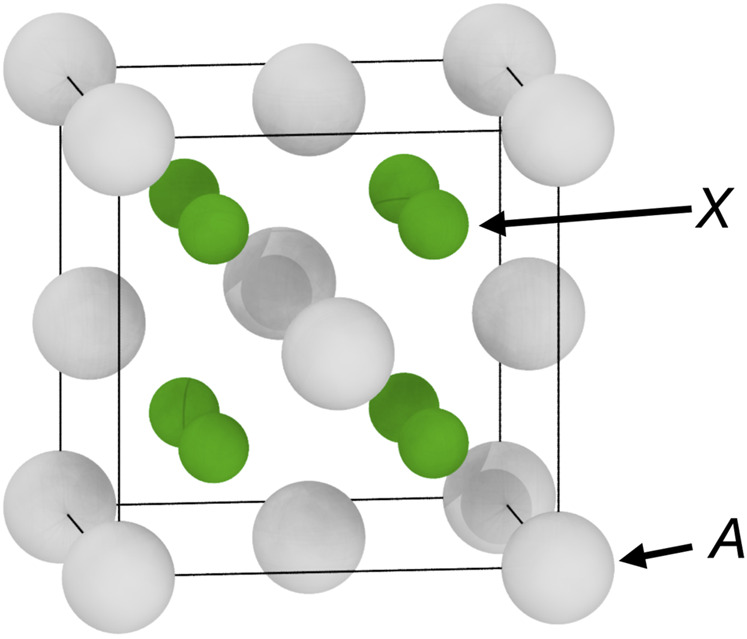
The fluorite structure (space group $Fm\bar{3}m$ ) for *AX*_2_ compounds, showing both *A* and *X* sublattices.

The fluorite structure (space group $Fm\bar{3}m$ ) is common for many compounds with the *AX*_2_ form (Hayes (1974)). In the classic fluorite structure, following the prototype CaF_2_, *A* is a cation—such as Ca^2+^, Sr^2+^, U^4+^ — whereas *X* is an anion—commonly F^−^, Cl^−^, or O^2−^. This structure can be viewed as the combination of a face-centred cubic (FCC) sublattice containing the *A* chemical species and a simple cubic (SC) sublattice with the *X* species, as shown in Figure 1. The anti-fluorite structure is very closely related, the only difference being that in this case *A* is an anion, and *X* is a cation. Both Na_2_O and Li_2_O are common examples of the anti-fluorite structure. However, this difference in ionic character does not change the geometry of the crystals. In the rest of the article, both structures will be referred to as either *fluorite* or $Fm\bar{3}m$ , but the conclusions apply equally to anti-fluorites.

Compounds with the fluorite structure have been very well studied for industrial applications (*e.g.* UO_2_, CeO_2_, yttria–stabilised zirconia), or because they are a simple model for ionic conductors (Hayes, 1974). Their tendency to behave as superionic conductors at high temperatures has been a particular area of interest since the 1970s. The nature of the high-temperature superionic phase in *AX*_2_ compounds with the fluorite structure is not fully explained yet, and conflicting interpretations exist. One of them is that high conductivity is the result of the availability of many crystalline sites for diffusion resulting from extensive Frenkel disorder and defects clustering on the *X* sublattice (Hutchings et al., 1984; Hull et al., 2011). Another view is that the superionic phase is the combination of a melted *X* sublattice and an *A* sublattice that retains its crystalline character, and thus the integrity of the material (Boyce et al., 1977; Castiglione and Madden, 2001). Recent works on the dynamical behaviour of the superionic phase of UO_2_ and Li_2_O seem to validate this description, showing string-like collective diffusion mechanisms otherwise common in supercooled liquids (Gray-Weale and Madden, 2004; Annamareddy and Eapen, 2017; Zhang et al., 2019). This interpretation is also strengthened by measurements of entropy changes during superionic transitions, which produced results consistent with some partial melting (O’Keeffe, 1976; Boyce et al., 1977).

In this work, we set out to investigate the structural aspects of the superionic transition in compounds with the fluorite structure. We aim to provide some insight on key structure changes happening at that transition and on the structural characteristics of the superionic phase. Molecular dynamics (MD) techniques have been recognised as a powerful tool for a very long time for the study of ionic conducting oxides (Catlow et al., 1978). It is particularly well-suited for this type of investigations that bridge thermodynamical properties and structural features. Indeed, its atomic-scale resolution makes accessible structure details such as point defects, local environments, or structure changes. This has resulted in a large collection of empirical potential that can be used to simulate a variety of compounds. Instead of focusing on one specific material, we considered a list of them for which successful potentials have been published. This way, we intend to describe phenomena that are characteristic of ionic compounds in the $Fm\bar{3}m$ structure rather than specific to one composition or one potential.

Structures from MD simulations of superionic phases can be very difficult to analyse because of temperature-induced effects. This includes not only structural defects that form normally at high temperatures, such as Frenkel pairs, Schottky trios, or larger clusters, but also the resulting strain fields that distort the structure, and large thermal vibrations. All of this obscures the underlying structure of the materials and makes any structural analysis challenging. To overcome this, we employed computational diffraction techniques to generate virtual X-ray diffraction (XRD) patterns. These provide another way of characterising structural features that are difficult to describe otherwise, by showing structures in the reciprocal space that are not subject to the same perturbations as the atomic positions at high temperatures.

## 2 Methods

### 2.1 Empirical Potentials

We selected a broad range of empirical potentials that have been designed to simulate crystals with the fluorite structure. Thanks to the sustained interest for *AX*_2_ materials with the $Fm\bar{3}m$ structure, a large number of such potentials are available and can be used for our purposes. The final set of compounds that can be simulated using the selected potentials included seven different chemical compositions, with some fluorides, oxides and one chloride. Most of these compositions were represented by several different potentials listed in Table 1 with their main characteristics.

**TABLE 1:** Empirical potentials used in this study.

Compound	Potential	Charges	Interactions
BaF2	Catlow and Norgett (1973) ^a^	Formal	Buck. + ZBL
	Cazorla et al. (2018)	Formal	Buckingham
	Sayle et al. (2003)	Formal	Buckingham
CaF_2_	Bingham et al. (1989)	Formal	Buckingham
	Catlow and Norgett (1973) ^a^	Formal	Buck. + ZBL
	Evangelakis and Pontikis (1989)	Formal	Buck. + ZBL
	Sayle et al. (2003)	Formal	Buckingham
Li_2_O	Asahi et al. (2014)	Partial	Buckingham
	Oda et al. (2007)	Partial	Buckingham
	Pedone et al. (2006)	Partial	Morse + repulsion
*β*-PbF_2_	Catlow et al. (1978) ^a^	Formal	Buckingham
SrCl_2_	Bendall and Catlow (1980) ^a^	Formal	Buckingham
	Gillan and Dixon (1980)	Formal	Buckingham
SrF_2_	Bingham et al. (1989)	Formal	Buckingham
	Catlow and Norgett (1973) ^a^	Formal	Buck. + ZBL
	Cazorla et al. (2018)	Formal	Buckingham
UO_2_	Cooper et al. (2014b) ^a^	Partial	Buck. + EAM
	Morelon et al. (2003)	Partial	4-terms Buck

a The Catlow and Bendall potentials originally had polarisable shells, which were ignored in this study.

The seven selected compounds include some variety in the properties of both *A* and *X* ions. The *X* ions were Li, F, Cl, or O, having electric charges of respectively +1, −1,−1, and−2. The ions on the *A* sublattice were O, Ca, Sr, Ba, Pb, or U with masses ranging from 16 u to 238 u, and with $\frac{A}{X}$ mass ratio from 2.31 (Li_2_O) to 14.88 (UO_2_). The potentials we used take different analytical forms. Most of the potentials, particularly older ones, use simple Buckingham interactions. However, they are not identical to each other and differ in the numerical values of their parameters and their fitting procedure. Indeed, they have been initially fitted to reproduce different properties such as defect energies at room temperatures, which are not necessarily related to the superionic transition. In some more recent potentials, the electric charges of the different species are a parameter that was adjusted during the potentials fitting in the same way as the parameters of the non-electrostatic pair interactions. This reflects the imperfect ionic nature of these materials. Amongst the potentials used in this study, it is the case for the potentials representing Li_2_O and UO_2_. Some potentials use analytical forms other than Buckingham, such as Morse (Pedone potential for Li_2_O), 4-terms Buckingham (Morelon potential for UO_2_), or additional embedded atom model (EAM) many-body contributions (CRG potential for UO_2_). The Pedone potential was not even designed to simulate the $Fm\bar{3}m$ structure, but instead complex minerals and glasses. The analytical form of the electrostatic interactions was the Wolf summation (Wolf et al., 1999).

In some instances, several potentials were proposed in the original references. This is the case for the Catlow potential for BaF_2_, CaF_2_, and SrF_2_, and the Oda potential for Li_2_O. For the Catlow potential, we chose the first model of the three proposed. The difference between the first and second model is only a different set of parameter for the core-shell interactions, and the third model showed less accurate lattice parameters at higher temperatures in preliminary simulations. For the Oda potential for Li_2_O, we selected the FIT-GGA parameters, which offered the most accurate thermal expansion and melting point.

All the potentials we used predicted the $Fm\bar{3}m$ structure as the ground state at room pressure, and showed reasonable thermodynamical properties. However, no assessment regarding the quality of the potentials, or their ability to reproduce quantitatively given materials properties, was made otherwise.

Some of the potentials were designed with polarisable core-shell models to improve dielectric properties and elastic constants. However, the shell models tested tended to show instabilities and large fluctuations of the thermodynamical properties at high temperatures. We therefore ignored core-shell interactions when using these potentials, and considered only rigid ions. This should not affect the structure at low temperatures because of the way the interactions were fitted. In fact, the first step in the potential design process was to adjust the pair interactions to reproduce the structure correctly (Catlow and Norgett, 1973). Adjusting the parameters for the shell models was done in a following step, to improve dielectric constants. In any case, these potentials were not intended to reproduce very disordered structures or melts, therefore they should not be less accurate in principle than other, rigid-ions only potentials at high temperatures.

In addition, the Catlow potentials for BaF_2_, CaF_2_, and SrF_2_, as well as the Evangelakis potential for CaF_2_ showed some instability at high temperatures, due to the relatively low energy barrier in their F–F Buckingham interactions. To avoid issues during the simulations with ions falling in the Buckingham singularity at short separation distances, a Ziegler-Biersack-Littmark (ZBL) contribution was added (Ziegler et al., 1985). We used the switching scheme implemented in LAMMPS with an outer radius of 2.0 Å and inner radii of 1.5 Å and 1.6 Å for the Catlow and Evangelakis potentials respectively. The Catlow potential for *α*-PbF_2_ was derived independently and did not suffer from the same issue, and was therefore not modified.

Considering this diversity of compounds and potentials, it is expected to see some variance in the properties and structures of each specific material depending on the potential used. However, this diversity is also expected to limit any bias in our results by separating effects shown with only some of the potentials from common features independent of potentials details. Our intent was to identify features and qualitative behaviour universal in crystals with the $Fm\bar{3}m$ structure, rather than finding the absolute most accurate potentials. For this reason, we privileged a larger number of potentials rather than a more limited selection. This means that our results should be interpreted in general in terms of trends across compounds and potentials, and not quantitative predictions of materials properties. We will point out when a given potential deviates significantly from experimental results, or from the behaviour of other potentials.

### 2.2 Molecular Dynamics Simulations

The input structures for the MD simulations were 10 × 10 × 10 supercells of the conventional $Fm\bar{3}m$ unit cell. This resulted in cubic simulation boxes with 12 ,000 atoms and sides around 50 Å long, depending on potential and temperature. With all the potentials, electrostatic interactions were calculated using the Wolf sum with a damping parameter of 0.3 Å^-1^ (Wolf et al., 1999). All pair interactions, including electrostatics, were truncated and shifted using a cutoff radius of 11 Å.

Equilibrium MD calculations were carried out from room temperature to the temperature at which only liquid phases were observed. This upper bound for the temperature range depends on both composition and potential. To sample these ranges similarly for each potentials, 50 evenly-spaced temperatures were selected in each case. The code used was LAMMPS (Plimpton, 1995), with both temperature and pressure controlled by the time-reversible Tuckerman integrator (Tuckerman et al., 2006) based on the Nosé-Hoover (Nosé, 1984) method and the Parrinello-Rahman strain energy (Parrinello and Rahman, 1981). When structure optimisation was useful, it was done by minimising the potential energy of the structure whilst keeping the simulation box fixed, using the implementation of the Broyden—Fletcher—Goldfarb—Shanno (BFGS) algorithm from the L-BFGS library (Byrd et al., 1995).

Each simulation consisted of a several successive relaxation steps. The first one was a constant-temperature, fixed-volume (*NVT*) equilibration for 10 ps in order to thermalise the simulation box. This was followed by a fixed-temperature and room-pressure (*NPT*) equilibration for 240 ps, during which the average box lengths were calculated. Both cell size and shape were allowed to fluctuate during this step in order to avoid constraining the relaxation process. Although the box shape could change during this step, it remained very close to cubic on average except in simulations of liquids. Then, the box shape was fixed to match the average shape calculated during the *NPT* step. Following this, each supercell underwent a last *NVT* relaxation for 10 ps. Finally, data was accumulated over a 20 ps simulation at constant volume and energy (*NVE*). The time step for all these simulations was 1 fs, and the damping time for the thermostat and barostat were 0.01 and 0.5 ps respectively. The enthalpy *H*(*T*) was determined for each temperature *T* in K by taking its average over the *NVE* runs. The constant-pressure heat capacity *C*_P_(*T*) was then determined by differentiating the enthalpy

$$C_{\text{P}}\left(T\right)=\frac{\partial H}{\partial T}\;.$$ (1)

This was done numerically using a second-order centred finite difference scheme. The lattice parameter *a*(*T*) was calculated by taking the average of the box size along the *x*, *y*, and *z* axes and dividing it by 10, the number of conventional cells along each direction. The linear thermal expansion coefficient

$$\alpha \left(T\right)=\frac{\partial \ln\left(a\right)}{\partial T}$$ (2)

was calculated using the measured lattice parameter, with the same numerical scheme employed to calculate *C*_P_(*T*). Eq. 2 was used instead of the equivalent and more common expression $\alpha =\frac{1}{a}\;\frac{\partial a}{\partial T}$ for the increased numerical precision of its finite differences discrete form. Statistical sampling of the phase space is important, particularly when considering highly disordered structures. For this reason, all simulations were run three times independently, with identical initial atomic positions but different initial velocities. The figures presented here are averages over these three simulations, but figures showing the complete data are available in the Supplementary Material for this article.

Diffusion being an important aspect of the superionic transition, we also calculated diffusion coefficients from the MD simulations. This was done by using the Einstein relation

$$D=\frac{1}{6}\;\underset{t\to \infty }{\lim}\;\frac{d}{dt}\left\langle |u\left(t\right){|}^{2}\right\rangle ,$$ (3)

where *t* is the simulation time, and **u** is the atomic displacement. The mean squared displacements ⟨|**u**(*t*)|^2^⟩ were calculated for both *A* and *X* species separately, to produce specific diffusion coefficients.

Point defects are an important structural feature at high temperatures. Since they play an important role in diffusion and have been linked to the onset of the superionic transition (Hiernaut et al., 1993), it is useful to be able to determine how common they are. Given that the structure considered here are stoichiometric to ensure electric charge neutrality and that the number of atoms is fixed and does not change over the course of a simulation, the only possible defects are Frenkel pairs, where an ion leaves its initial lattice site to form an interstitial elsewhere in the crystal. Counting the number of Frenkel pairs requires counting either the number of vacancies or the number of interstitials. We did so by mapping each ion in structures of interest taken from MD simulations to a reference crystal, which was a $Fm\bar{3}m$ structure with the same composition and lattice parameter. Thus, there are three possibilities for each lattice site. It can be: (i) empty, indicating the presence of a vacancy; (ii) occupied by one ion; or (iii) occupied by two ions, which indicates the presence of an interstitial. This analysis has been performed separately for the *A* (FCC) and *X* (SC) sublattices. It can result in principle in under-counting defects compared to experimental methods, if some ions are displaced enough to leave their site but not enough to be mapped onto another one. We also did not consider the interstitial octahedral site as a separate site for the purpose of the defects analysis.

We performed additional structure analysis using the polyhedral template matching (PTM) (Larsen et al., 2016) technique implemented in Ovito (Stukowski, 2010). This method assigns a kind of symmetry environment to each atom by comparing the relative positions of its neighbours to reference polyhedra for prototype structures such as simple cubic, hexagonal close packed, body-centred cubic, or face-centred cubic. This was used to show the structural disorder across the superionic transition. The RMSD cutoff for the PTM analysis was set to 0.5 for all structures.

### 2.3 Diffraction Patterns

Powder diffraction patterns were generated using the Debyer code, which implements the Debye scattering equations (Farrow and Billinge, 2009; Wojdyr, (https://github.com/wojdyr/debyer)). We use the copper *Kα*_1_ wavelength *λ* = 1.54056 Å for these calculations. In order to increase the signal-to-noise ratio, the 12 ,000-atom fluorite cells were first duplicated 7 times in all three directions, resulting in 4,116 000-atoms supercells. To investigate separately the structures of the *A* and *X* sublattices, additional supercells were set up by copying the full supercells and then removing either the cations or the anions. Thus, for each simulation we calculated a full pattern accounting for all the atoms, plus partial patterns representing respectively the structures of the *A* and *X* sublattices. A sinc damping function was applied to the radial distribution functions. This, in combination with the supercells, is helpful to limit artefacts from the Fourier transform, which would otherwise be large if the 12 ,000-atoms boxes had been used instead. We set the cutoffs for the Fourier transforms to less than one half of the lengths of the supercells. The intensity was finally plotted in powder XRD patterns as a function of the scattering angle 2*θ*.

Two-dimensional single crystal diffraction patterns were generated using the approach described in (Jin et al., 2020). So called reciprocal space maps (RSMs) were computed in the *HHL* planes, where *H* and *L* are non-integer multiples of the reciprocal lattice unit cell of a given fluorite structure $1/a_{AX_{2}}\left(T\right)$ , where $a_{AX_{2}}\left(T\right)$ is the lattice parameter of the *AX*_2_ crystal at the considered temperature *T*. In order to increase the signal-to-noise ratio the *HHL* planes have a “thickness” of $\frac{1}{4}$ reciprocal lattice unit cell over which the intensity was integrated. The RSMs were computed with the 12 ,000–atoms cells which results in a visible broadening of the reciprocal lattice points, and the presence of finite box size interference fringes, especially visible at low disorders.

In order to quantify the intensities of the different reflections observed in the RSMs, line scans (with a 0.2 width in *HKL* units) were first extracted from the 2D data. These scans were further modelled with pseudo -Voigt functions— *i.e.* a linear superposition of a Gaussian and a Lorentzian function—to represent the Bragg peaks, an asymmetrical Gaussian function to represent the isotropic diffuse scattering and an additional linear background. The intensities were obtained after a conventional least-square fitting of the model to the 1D scans.

## 3 Results and Discussion

### 3.1 Thermodynamical Properties

As a first step, the general behaviour of the potentials was assessed by calculating their thermodynamical properties as a function of temperature: enthalpy *H*, constant-volume heat capacity *C*_P_, and thermal expansion coefficient *α*. These were used to verify that all potentials predicted a superionic transition for their compound, and to estimate the transition temperature *T*_S_.

Some room-temperature properties calculated using the empirical potentials are shown in table 2. The constant-pressure heat capacities are very similar across the potentials, and close to the value expected from the Dulong–Petit law of 2.59 ⋅ 10^–4^ eV/(atom K). This indicates a strong harmonic behaviour from all the potentials, consistently with experimental observations. The main discrepancy with experiments is with Li_2_O, which has a significantly smaller heat capacity. The linear thermal expansion coefficient is more potential-dependent than the heat capacity. In general, the models tend to over-estimate *α*, sometimes significantly. For example, the Cazorla and Sayle potentials for BaF_2_, as well as the Bingham and Sayle potentials for CaF_2_, the Gillan potential for SrCl_2_, and the Bingham potential for SrF_2_ have errors between 40 and 50%. This is not surprising, considering that *α* is very sensitive to the details of the potentials, and that the parameters are almost never fitted to it. Overall, the values for both *C*_P_ and *α* show that all the potentials behave well at room temperature, even though some are more accurate than others for these properties.

**TABLE 2:** Thermophysical properties at room temperature for the different potentials, compared to experimental references: (1) Wicks and Block (1963); (2) Roberts and White (1986); (3) Dworkin and Bredig (1968); (4) Johnston and Bauer (1951); (5) Hull et al. (1988); (6) Popov et al. (2017); (7) Schröter and Nölting (1980); (8) Smith et al. (1963); (9) Pavlov et al. (2017); (10) Martin (1988).

Compound	Potential	*C*_P_/10^–4^ eV/(atom K)		*α*/10^–5^ K^–1^	
		This work	Exp	This work	Exp
BaF_2_	Catlow and Norgett (1973)	2.69	2.44^(^^1)^	1.82	1.96^(^^2)^
	Cazorla et al. (2018)	2.71		2.93	
	Sayle et al. (2003)	2.71		2.92	
CaF_2_	Bingham et al. (1989)	2.72	2.66^(^^3)^	2.49	1.90^(^^2)^
	Catlow and Norgett (1973)	2.70		1.71	
	Evangelakis and Pontikis (1989)	2.71		1.96	
	Sayle et al. (2003)	2.70		2.34	
Li_2_O	Asahi et al. (2014)	2.69	1.80^(^^4)^	2.82	2.78^(^^5)^
	Oda et al. (2007)	2.70		2.34	
	Pedone et al. (2006)	2.78		3.25	
*β*-PbF_2_	Catlow et al. (1978)	2.71	2.56^(^^1)^	2.29	2.85^(^^6)^
SrCl_2_	Bendall and Catlow (1980)	2.69	2.67^(^^7)^	2.82	2.50^(^^2)^
	Gillan and Dixon (1980)	2.81		3.57	
SrF_2_	Bingham et al. (1989)	2.70	2.43^(^^8)^	2.57	1.97^(^^2)^
	Catlow and Norgett (1973)	2.68		1.72	
	Cazorla et al. (2018)	2.67		2.09	
UO_2_	Cooper et al. (2014b)	2.56	2.14^(^^9)^	0.97	0.976^(^^10)^
	Morelon et al. (2003)	2.60		1.13	

The simulation were performed in a temperature range from 300 K to the temperature *T*_M_ at which the initial crystal turned into a liquid. This transition does not correspond to the experimental melting point *T*_m_, and is in general higher as shown in Table 2. By definition, the melting point measured experimentally is the temperature at which the liquid becomes thermodynamically more favourable than the crystal. However, this does not mean that the crystal is strictly unstable: it can be metastable instead and require climbing a non-negligible energy barrier to melt. The small length-scale and short times considered in our simulations inhibits nucleation of a liquid phase, which is the first step of a usual melting transition, by preventing the structure from climbing that energy barrier. The solid to liquid transition we observe is more analogous to a mechanical melting mechanism, and indicate the temperature at which the crystal becomes unstable rather than metastable (Wang et al., 1997). Other methods, such as the so-called moving interface technique (Morris et al., 1994), would be required to measure the thermodynamical melting points *T*_m_ accurately. For this reason and to avoid confusion, the thermodynamical (experimental) and mechanical melting points will be noted *T*_m_ and *T*_M_ respectively throughout this article.

**FIGURE 2:**
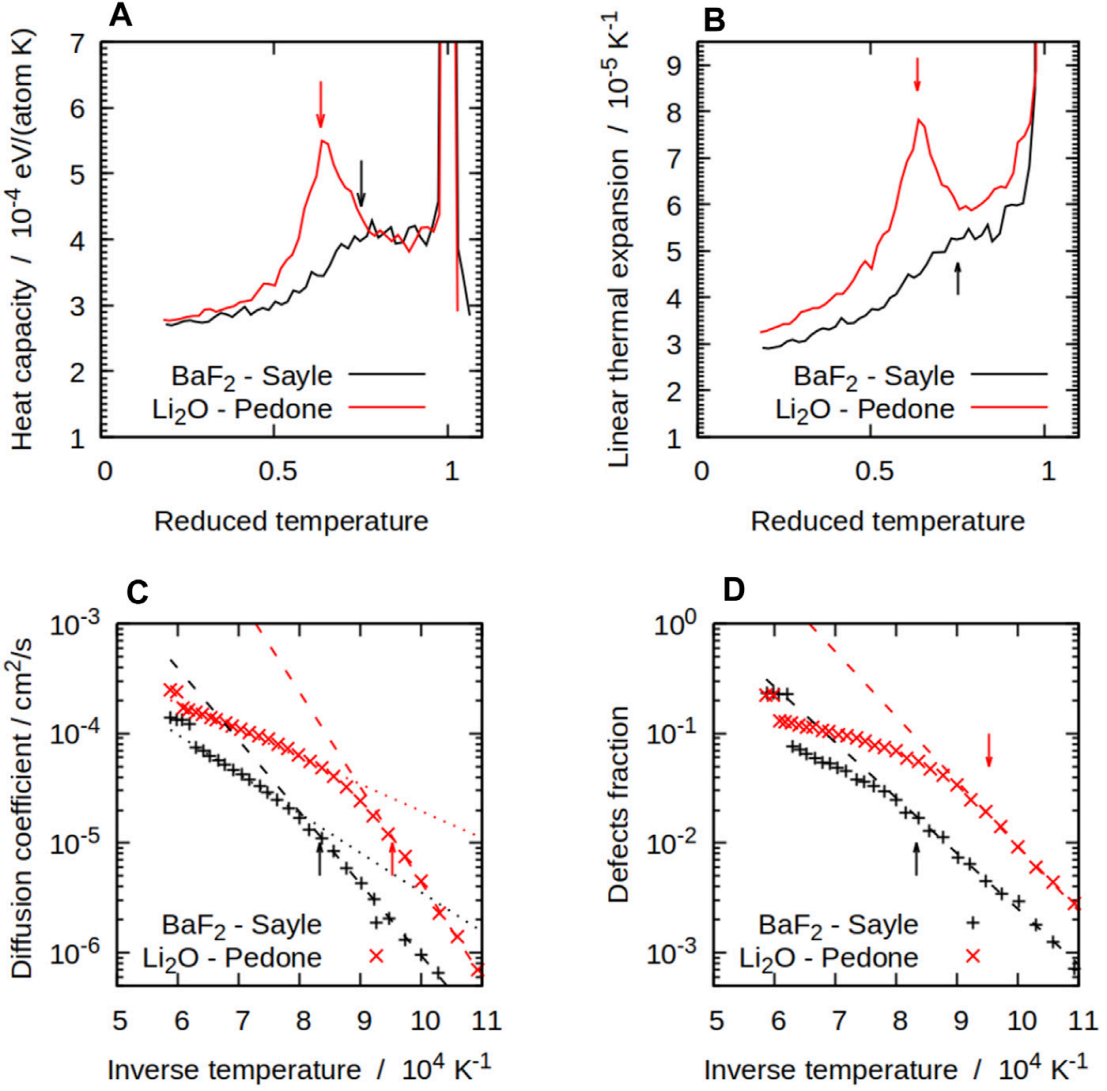
Some thermodynamical and structural properties of compounds with the $Fm\bar{3}m$ structure, BaF_2_ with the Sayle potential (Sayle et al., 2003) and Li_2_O with the Pedone potential (Pedone et al., 2006): **(A)** constant-pressure heat capacity; **(B)** linear thermal expansion coefficient; **(C)** diffusion coefficient; **(D)** numbers of defects. Reduced temperatures in **(A)** and **(B)** are $\frac{T}{T_{\text{M}}}$ , where *T*_M_ is the mechanical melting temperature of each compound. The arrows indicate the superionic transition temperatures *T*_S_.

The most visible manifestation of the superionic transition from an experimental point of view is the Λ-shaped peak in *C*_P_, or equivalently the inflection point in the enthalpy *H*(*T*). Such peaks have been reported for several fluoride (Hull, 2004), chloride (Schröter and Nölting, 1980; Hull et al., 2011), and oxide (Ralph, 1987) compounds with the $Fm\bar{3}m$ structure. Amongst the potentials considered here, some, such as the Pedone potential for Li_2_O shown in Figure 2, showed a well-defined, sharp peak as generally expected. It was not as clear for other potentials such as the Gillan potential for SrCl_2_, the Cazorla potential for BaF_2_, or the Sayle potential for BaF_2_, which is also shown in Figure 2. These potentials actually showed a plateau instead of a peak, and the superionic transition is not very visible when looking at thermodynamical properties for such potentials. The other potentials show intermediate behaviours, with *C*_P_ peaks with various degrees of sharpness. Two potentials for the same compound can have different *C*_P_ characteristics. An example of this is BaF_2_, where the Catlow potential has a very sharp peak, whilst no peak is visible with the Cazorla potential. Figures equivalent to Figure 2 for the other potentials can be found in the Supplementary Material for this article. A similar behaviour, also with a sharp Λ-shaped peak, was observed in the linear thermal expansion coefficient *α*(*T*). Interestingly, the potentials showing a strong *C*_P_ peak similarly tend to show a clear *α* peak at the same temperature. From the *C*_P_(*T*) and *α*(*T*) curves, we can define four qualitatively different temperature ranges:

i) a low-temperature regime, in which the heat capacity varies slowly as a polynomial;
ii) an exponential increase leading to the top of the Λ peak and the superionic transition, attributed to the formation of Frenkel defects (Szwarc, 1969);
iii) a decrease forming the right-side of the Λ peak;
iv) a high-temperature regime in which *C*_p_ varies slowly, leading to *T*_M_.

Some potentials show only some of these features. For instance, regime (i) can be very short for potentials with a low superionic transition temperature. The potential in which the *C*_P_ peak is not visible would not have a clear stage (iii), instead going directly from (ii) to (iv). For the potentials that show all four stages, the *C*_P_ peak is the simplest way of measuring the superionic transition temperature *T*_S_, which is by convention the temperature corresponding to the top of the peak. This is naturally more difficult to measure for potentials that show a plateau or no peak at all.

The potentials show significant variation in their predictions for *T*_S_, with differences of ∼300 K between the lowest and highest values for each compound. This highlights the variety of purposes for which the potentials were designed. More recent potentials tend to be more accurate, presumably thanks to more sophisticated potentials fitting techniques. Potentials that were intended to reproduce the superionic transition, such as Oda for Li_2_O, are unsurprisingly the most accurate. Overall, the trend across materials is reproduced, with SrCl_2_ and UO_2_ having respectively a lower and a higher *T*_S_ on average compared to the other materials. The main outlier is the Catlow potential for *β*-PbF_2_, which overestimates *T*_S_ by 700 K. However, few other potentials are available for this compound, which makes the discussion of any potential-dependent effect difficult. It should also be noted that Pb^2+^ ions in PbF_2_ are quite polarisable, which could point to a limitation of the rigid ions models to simulate this material. This point has been made in the literature (Castiglione and Madden, 2001), where calculations were done using shell models, with results closer to experimental references. The mechanical melting temperatures *T*_M_ are not available experimentally, therefore those obtained from our simulations cannot be compared directly with references. We could still verify that they were greater than the reported melting temperatures *T*_m_ for each compound.

The transition temperature *T*_S_ has been linked to the melting point *T*_m_. The values for both *T*_S_ and *T*_M_ from our simulations are summarised in Table 3 and compared with experimental values. There is some variation across potentials, but overall there is a qualitative agreement for *T*_S_, *i.e.* compounds with a high transition temperature experimentally also tend to have higher values using empirical potentials. It has been reported that the $\frac{T_{\text{S}}}{T_{\text{m}}}$ ratio is generally around 0.8 (Hiernaut et al., 1993). However, this is not very accurate, as experimental references summarised in Table 3 show $\frac{T_{\text{S}}}{T_{\text{m}}}$ ratios between 0.63 (for *β*-PbF_2_) and 0.85 (for SrF_2_ and CaF_2_). Whilst we did not measure the thermodynamical melting points *T*_m_ that would be directly comparable with experiments, we did measure the mechanical melting points *T*_M_. The $\frac{T_{\text{S}}}{T_{\text{M}}}$ ratios from our simulations are similarly scattered. They are potential-dependent, and range from 0.57 (for CaF_2_ with the Evangelakis potential) to 0.79 (for SrCl_2_ with the Bendall potential).

**TABLE 3:** Superionic transition temperatures *T*_S_ for the different potentials, compared to experimental references. Superionic transition temperatures were calculated from *C*_P_(*T*) curves, except for those marked.

Compound	Potential	*T*_S_/K		*T*_m_/K	*T*_M_/K
		This work	Ref	Ref	This work
BaF_2_	Catlow and Norgett (1973)	1,550	1,135^b^ ^(1)^	1,350^b^ ^(1)^	2,290
	Cazorla et al. (2018)	1,200^a^	1,275^(2)^	1,628^(2)^	1,630
	Sayle et al. (2003)	1,200^a^	1,300^(3)^	1,555^(3)^	1,600
CaF_2_	Bingham et al. (1989)	1,300^a^	-	1,660^b^ ^(1)^	1,910
	Catlow and Norgett (1973)	1,650	1,400^b^ ^(1)^		2,670
	Evangelakis and Pontikis (1989)	1,400	1,430^(5)^	1,696^(2)^	2,430
	Sayle et al. (2003)	1,300^a^		1,633^(4)^	1980
Li_2_O	Asahi et al. (2014)	1,275	1,350^(6)^	1,703^(7)^	1,910
	Oda et al. (2007)	1,300			1,950
	Pedone et al. (2006)	1,050			1,650
*β*-PbF_2_	Catlow et al. (1978)	1,400^a^	711^(8)^	1,128^(2)^	1,950
SrCl_2_	Bendall and Catlow (1980)	1,200^a^	1,001^(5)^	1,148^(2)^	1,510
	Gillan and Dixon (1980)	920^a^		1,146^(4)^	1,290
SrF_2_	Bingham et al. (1989)	1,200^a^	1,150^b^ ^(1)^	1,430^b^ ^(1)^	1,810
	Catlow and Norgett (1973)	1,630	1,370^(3)^	1,746^(2)^	2,530
	Cazorla et al. (2018)	1,500^a^	1,400^(2)^	1,723^(4)^	2,080
UO_2_	Cooper et al. (2014b)	2,550	2,670^(9)^	3,126^(10)^	3,440
	Morelon et al. (2003)	2,600^a^			4,100

a which were estimated from *D*(*T*) curves. The reference values are from experiments, except for those marked.

b which come from DFT calculations. The *T*_M_ temperatures are the mechanical melting points from our simulations. Whilst they are not directly comparable, we also provide experimental melting points *T*_m_ for context. Experimental references are: (1) Cazorla et al. (2018); (2) Lidiard (1980); (3) Thomas (1976); (4) Catlow et al. (1978); (5) Dworkin and Bredig (1968); (6) Hull et al. (1988); (7) Kurasawa (1982); (8) Dickens et al. (1982); (9) Hiernaut et al. (1993); (10) Böhler et al. (2014).

### 3.2 Point Defects and Diffusion

We calculated the fraction of point defects as a function of temperature for each potential. In general, we found as expected that the fraction of *A* defects was negligible in most of the considered temperature ranges (Catlow and Norgett (1973)). In all cases, the number of *A* Frenkel pairs just below *T*_M_ was still two orders of magnitude smaller than that of *X* defects. No defect at all could be seen on the *A* sublattice for temperatures lower than about 100 K below *T*_S_, therefore they will be ignored and the following discussion is focused on the *X* defects.

Populations of *X* defects were significant at higher temperatures with all the potentials we considered. Figure 2D shows this for both BaF_2_ and Li_2_O with the Sayle and Pedone potentials respectively. The simulations showed a pattern with a transition from a low-temperature Arrhenius regime, in which the *X* defect fractions takes the form

$$N_{def}\left(T\right)=N_{0}\;e^{-H_{f}/2k_{B}T}\;,$$ (4)

where *H*_f_ is the formation enthalpy of an *X* Frenkel pair, to a high-temperature regime in which the number of defects grows with a smaller effective formation energy. The effective formation enthalpy below the superionic transition *H*_f_ is shown for all potentials in Table 4. The difference between both regimes and the inflection point can be seen in Figure 2D. The *T*_S_ temperature marks the start of the deviation of the number of defects calculated from MD form its low-temperature fit. This links the change in defect properties to the onset of the superionic transition. The same pattern was observed for all potentials, with some variation in the magnitude of the transition. In general, potentials showing a sharp *C*_P_ peak also had a large difference between the low-temperature and high-temperature behaviours. On the other hand, in cases such as SrCl_2_ with the Gillan potential, the transition is not very noticeable and there is not a large difference between both regimes. At low temperatures, Equation 4 can be fitted to data from the MD simulations to estimate the formation energy of *X* Frenkel pairs. Formation enthalpies for *X* Frenkel pairs for all the considered potential are summarised in Table 4. In general, the empirical potentials are in qualitative agreement with available references and their formation enthalpies fall within the range of experimental values. Notable outliers are the Catlow and Cooper potentials for BaF_2_ and UO_2_, which significantly over-estimate *H*_f_, and Pedone for Li_2_O, which under-estimate it. Beyond the superionic transition, the *X* sublattice is expected to be highly disordered. However, defects are detected using the $Fm\bar{3}m$ structure as a reference, which may be inadequate in presence of large distortions and possible extended defects. For this reason, the number of defects is much less reliable in the superionic phase, and no attempt was made at obtaining a quantitative measurement of the number of point defects in this case.

**TABLE 4:** Formation enthalpies for *X* Frenkel pairs in the fluorite structures (*H*_f_). Experimental references are: (1) Lorger et al. (2019); (2) Bollmann (1980); (3) Lidiard (1974); (4) Farley et al. (1991); (5) Chadwick et al. (1988); (6) Samara (1979); (7) Boyce et al. (1977); (8) Bollmann et al. (1970); (9) Matzke (1987).

Compound	Potential	*H*_f_/eV	
		This work	Exp
BaF_2_	Catlow and Norgett (1973)	3.67	1.6^(1)^, 1.81^(2)^, 1.9^(3)^
	Cazorla et al. (2018)	2.07	
	Sayle et al. (2003)	2.01	
CaF_2_	Bingham et al. (1989)	2.58	2.2–2.8^(3)^, 2.43^(2)^
	Catlow and Norgett (1973)	2.99	
	Evangelakis and Pontikis (1989)	1.69	
	Sayle et al. (2003)	1.26	
Li_2_O	Asahi et al. (2014)	2.98	1.8^(1)^, 2.1^(4)^, 2.53^(5)^
	Oda et al. (2007)	2.30	
	Pedone et al. (2006)	1.17	
*β*-PbF_2_	Catlow and Norgett (1973)	2.93	0.89^(6)^, 1.06^(7)^
SrCl_2_	Bendall and Catlow (1980)	2.64	1.6–1.8^(3)^, 1.26–5.0^(2)^
	Gillan and Dixon (1980)	1.64	
SrF_2_	Bingham et al. (1989)	2.37	1.74^(8)^, 2.05^(2)^, 1.7–2.3^(3)^
	Catlow and Norgett (1973)	3.65	
	Cazorla et al. (2018)	3.87	
UO_2_	Cooper et al. (2014b)	8.58	2.6–4.2^(2)^, 5.6^(9)^
	Morelon et al. (2003)	4.18	

Crystals in the $Fm\bar{3}m$ structure are known to exhibit three different diffusion behaviours (Voronin and Volkov, 2001). The diffusion is extrinsic when it is limited by the availability of extrinsic defects to provide diffusion pathways. It becomes intrinsic, with a higher activation energy, when the temperature is high enough to provide a significant population of Frenkel pairs. Diffusion is then mediated by point defects hopping, and depends on the availability of *X* Frenkel pairs. Finally, in the superionic phase, diffusion is faster and is thought to involve collective displacements (Annamareddy and Eapen, 2017), with a lower activation energy than the intrinsic Frenkel pair mechanism (Voronin, 1995; Voronin and Volkov, 2001). Whilst our simulations could not reproduce extrinsic diffusion mechanisms because of the lack of extrinsic defects, we found intrinsic and superionic behaviours consistent with what could be expected from the literature. Both behaviours are visible in Figure 2C. We fitted exponential functions of the form $D\left(T\right)=D_{0}\;e^{-E_{\text{a}}/k_{\text{B}}T}$ to the calculated diffusion coefficients on both sides of the superionic transition to obtain diffusion activation energies $E_{\text{a}}^{\text{c}}$ and $E_{\text{a}}^{\text{s}}$ for the perfect crystal and the superionic phase respectively. The resulting activation energies are shown in Table 5. All the potentials considered here showed an activation energy change during the superionic transition, with $E_{\text{a}}^{\text{c}}>E_{\text{a}}^{\text{s}}$ , albeit to different degrees. The crossover between the low- and high-temperature behaviours does not occur at *T*_S_ for the potentials for which *T*_S_ could be calculated from the Λ peak in the *C*_P_(*T*) curves. Instead, as was the case for the number of defects, *T*_S_ marks the point at which the diffusion coefficients from MD start deviating from the low-temperature fit. The switch to the high-temperature behaviour itself occurs over a temperature range of a few hundred Kelvins. The upper bound of this range corresponds to the transition between regimes (iii) and (iv) as described in Section 3.1, *i.e.,* to the high-temperature end of the Λ peak. For potentials that do not show a clear peak in *C*_P_(*T*), the change in diffusion coefficients can be used to estimate *T*_S_. This method is less accurate, however, because the deviation of *D*(*T*) to the fit is more difficult to characterise than a clear peak of *C*_P_. All the transitions temperatures are summarised in Table 3.

**TABLE 5:** Activation energies for the *X* diffusion process in both the perfect fluorite phase ( $E_{\text{a}}^{\text{c}}$ ) and the superionic states ( $E_{\text{a}}^{\text{s}}$ ). The reference values are from experiments, except for those marked.

Compound	Potential	$E_{\text{a}}^{\text{c}}$ /eV		$E_{\text{a}}^{\text{s}}$ /eV	
		This work	Exp	This work	Ref
BaF_2_	Catlow and Norgett (1973)	2.90	1.6^(^^1)^, 2.52^(^^2)^	0.61	0.48^(2)^
	Cazorla et al. (2018)	1.32		0.95	
	Sayle et al. (2003)	1.30		0.73	
CaF_2_	Bingham et al. (1989)	1.52	1.92^(^^3)^, 2.02^(^^1)^, 3.34^(^^2)^	0.86	0.74^(2)^
	Catlow and Norgett (1973)	2.89		0.65	
	Evangelakis and Pontikis (1989)	2.19		0.61	
	Sayle et al. (2003)	1.63		0.82	
Li_2_O	Asahi et al. (2014)	2.02	1.6^(^^4)^, 2.52^(^^5)^	0.63	0.98^a^ ^(6)^
	Oda et al. (2007)	1.68		0.67	
	Pedone et al. (2006)	1.50		0.48	
*β*-PbF_2_	Catlow and Norgett (1973)	1.95	1.04^(^^7)^	0.81	-
SrCl_2_	Bendall and Catlow (1980)	1.89	4.43^(^^2)^	0.83	0.42^(2)^
	Gillan and Dixon (1980)	1.07		0.86	
SrF_2_	Bingham et al. (1989)	1.80	1.81^(^^3)^	0.79	0.55^(2)^
	Catlow and Norgett (1973)	2.76		0.63	
	Cazorla et al. (2018)	2.04		0.95	
UO_2_	Cooper et al. (2014b)	5.35	2.46–2.57^(^^8)^	1.43	
	Morelon et al. (2003)	2.74		1.40	

a which come from DFT calculations. References are: (1) Ando et al. (1980); (2) Voronin and Volkov (2001); (3) Bollmann et al. (1970); (4) Lorger et al. (2019); (5) Oishi et al. (1979); (6) Gupta et al. (2019); (7) Samara (1979); (8) Ando and Oishi (1983).

The activation energies in the $Fm\bar{3}m$ phases $E_{\text{a}}^{\text{c}}$ from the MD simulation agree with the experimental references in general.

The fact that all potentials showed two different regimes for diffusion of the *X* chemical species regardless of the presence of a peak in *C*_P_ and *α* confirms that the superionic transition can hapen without resulting in Λ peaks. This means that it is not inconceivable that such a transition could also happen without Λ peak in real materials. In fact, there are reports of this in SrCl_2_ (Dent et al., 2004).

### 3.3 Simulated Powder XRD Patterns

As mentioned previously, the analysis of the structural evolution of *AX*_2_ fluorites with temperature by a direct analysis of the MD simulations proved very challenging, particularly in the superionic phases. An approach to avoid this issue is to visualise the information contained in MD cells in a more compact representation. This is for instance the case when considering the radial distribution functions (RDFs). In the present study we choose to compute powder XRD patterns, *i.e.*, Fourier transforms of RDFs. They are more sensitive to long-range atomic order and its alteration (Debelle et al., 2014), and are therefore a very useful complement to real-space structure analysis. We illustrate our findings with the example of BaF_2_ with the Sayle empirical potential, bearing in mind that all conclusions drawn below are qualitatively valid for all the other potentials. This particular case was selected firstly because it exhibits XRD features common to all the composition–potential combinations investigated, with clearly visible temperature-dependent evolutions. It also has a plateau in *C*_P_ and *α* instead of a Λ peak. It is hence both a representative example of all configurations investigated in this work, and a demonstration of a superionic transition without a Λ peak.

**FIGURE 3:**
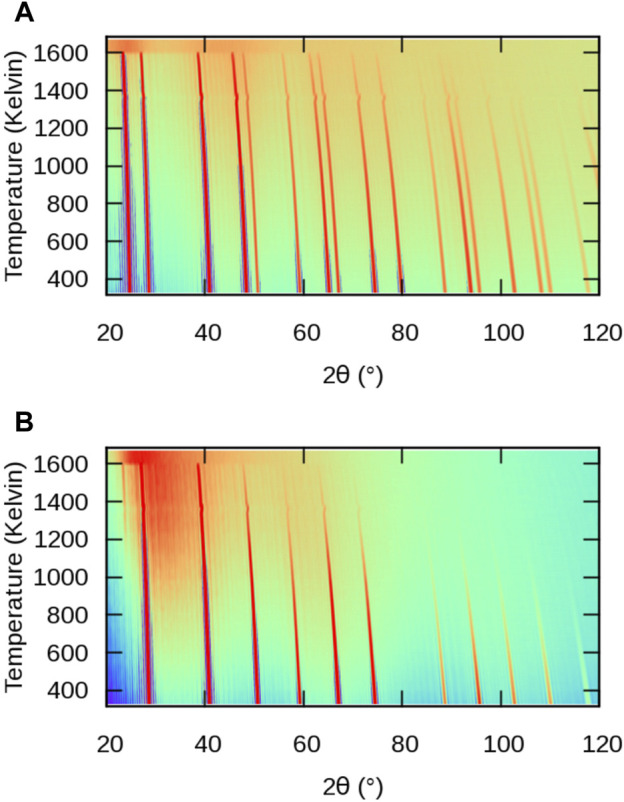
Simulated powder XRD patterns from MD simulations of BaF_2_ using the Sayle potential (Sayle et al., 2003) as a function of temperature: **(A)** full structure; **(B)** F sublattice only.

**FIGURE 4:**
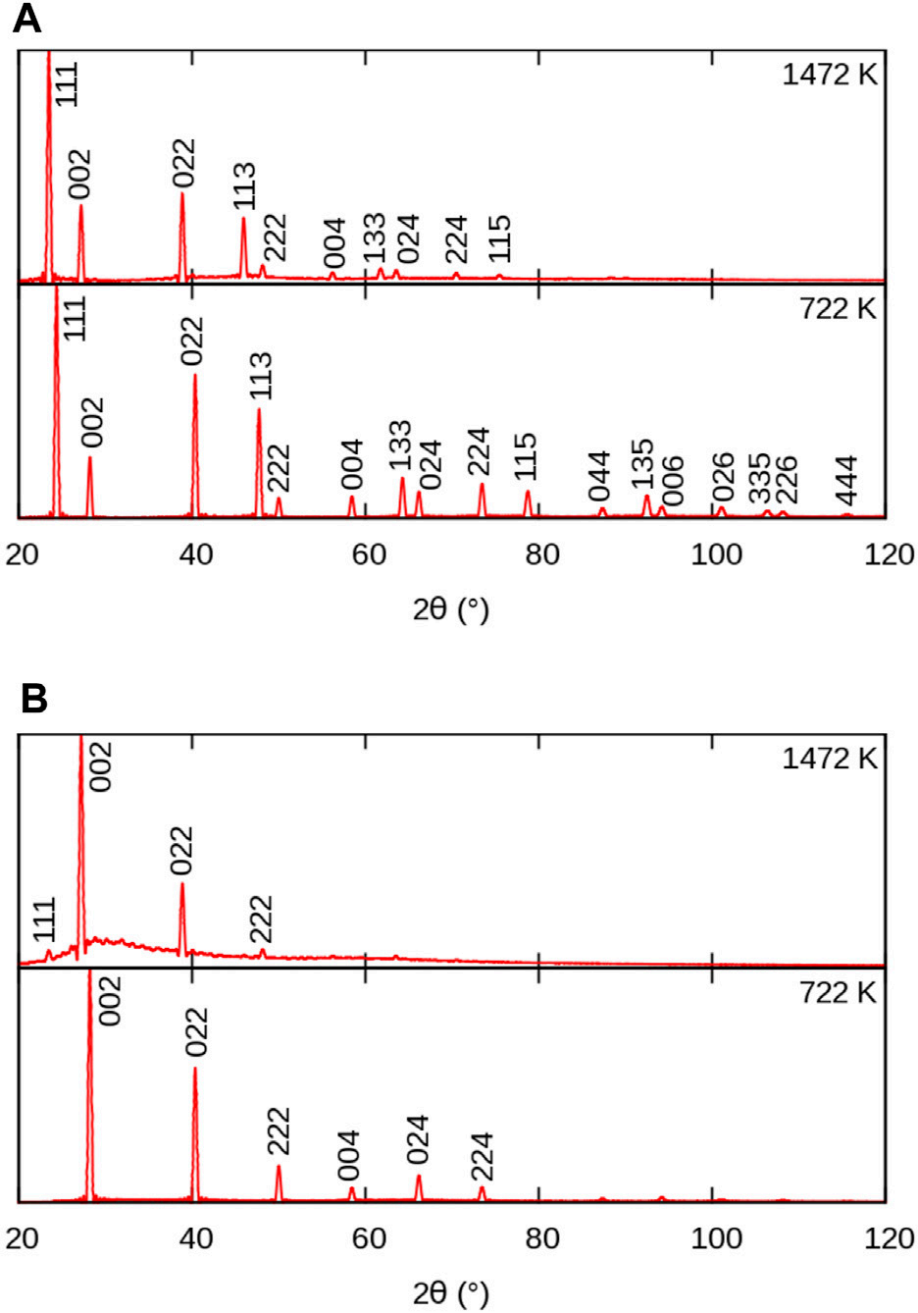
Simulated powder XRD patterns from MD simulations of BaF_2_ using the Sayle potential (Sayle et al., 2003), at temperatures below and above the superionic transition: **(A)** full crystal; **(B)** F sublattice only. Peak indices refer to the full $Fm\bar{3}m$ structure. The superionic transition temperature for this potential is 1,300 K.

The evolution of powder XRD patterns as a function of temperature is illustrated in Figure 3 (2*θ*–*T* figure), considering all atoms in the MD cell (Figure 3A) and only the *X* sublattice (Figure 3B). We will refer to them as full-crystal XRD and *X*-only XRD patterns, respectively. Additionally, we report selected full XRD and *X*-only XRD patterns at two temperatures on Figure 4.

Let us first consider the full XRD patterns shown in Figures 3, 4 A. At 300 K, the characteristic peaks of the fluorite unit cell are clearly visible and can be indexed within the $Fm\bar{3}m$ symmetry group. Unsurprisingly, these peaks gradually shift towards smaller angles with increasing temperature, following the thermal expansion of the material. This is more pronounced at higher angles in agreement with the derivative of Bragg’s law

Δ2θ=−2αΔT⁡tan⁡θ, (5)

Δ*T* being the temperature change.

The intensity of the peaks also decreases with increasing temperature, as a result of increasing local disorder, *i.e.* increasing thermal motion. The decrease in intensity is quantified by the Debye-Waller factor (Trueblood et al., 1996), which, in the case of harmonic and isotropic vibrations, is expressed by the simple expression

$$T\left(\theta \right)=\exp\left[-8\pi^{2}\left\langle u^{2}\right\rangle \sin^{2}\theta /\lambda^{2}\right]$$ (6)

where *u* is the displacement of the atom from its regular lattice site. The effect of intensity damping is more pronounced at higher angles, as was the case for the peak shift.

Finally, it can be noticed that a background of diffuse scattering develops around the most intense peaks. The intensity of this background increases up to the melting point where only diffuse scattering remains, but with a significantly redistributed intensity. The origin of this diffuse scattering background lies in the presence of thermal motion also responsible for the above-mentioned intensity damping, but it can also reveal the presence of structural defects.

In all the cases we investigated, the *A*-specific pattern was virtually identical to the full-crystal pattern. For this reason, the *A* patterns will not be discussed here, but 2*θ* − *T* maps can be found int the Supplementary Material. The similarity between full-crystal and *A*-only patterns can be simply understood by considering the difference in atomic numbers *Z* between light elements constituting the *X* sublattice (F, Cl, O), as compared to the heavier elements occupying the *A* sublattice (Ca, Ba, Pb, U). The latter dominate the overall XRD signal the intensity of which is proportional to *Z* ^2^, thus making the contribution from lighter elements fainter.

Let us now consider the *X*-only XRD patterns shown in Figures 3, 4 B. Besides the similar general features related to thermal expansion (peak shift) and thermal motion (decrease in intensity) there are two noticeable differences with the full-crystal patterns. The first difference is the positions and intensities of the peaks. This is easily explained by the fact that the *X* sublattice has a SC symmetry, as opposed to the FCC symmetry of the full fluorite structure. The lattice parameter of the SC sublattice is half of that of the overall $Fm\bar{3}m$ structure. To avoid any ambiguity, we index the peaks of the *X*-only patterns using the same Miller indices as the full fluorite structure. This means that, for instance, the peak labeled 200 would correspond to the 100 peak of a SC unit cell of the *X* sublattice.

The second difference between *X* and full-crystal powder XRD patterns is the diffuse scattering background. Its intensity relative to the Bragg peaks is much higher, which indicates a much higher level of disorder on the *X* sublattice than on the *A* sublattice. The diffuse scattering is also more broadly distributed across the 2*θ* range, roughly centred around the 200 peak. This is a sign of the presence of amorphous or highly disordered local environments with a first neighbour distance approximately given by the *X*–*X* distance in the corresponding sublattice.

An additional 111 peak (or $\frac{1}{2}$ $\frac{1}{2}$ $\frac{1}{2}$ in a base SC cell) emerges at temperatures around *T*_S_ in the *X*-only patterns. This peak could not be detected in the full XRD pattern because its position exactly coincides with the 111 peak of the fluorite structure. The appearance of a superstructure peak with halved Miller indices points to a doubling of the periodicity of the unit cell of the *X* sublattice, which becomes equal to that of the overall $Fm\bar{3}m$ crystal. However, the lack of other superstructure peaks such as 113 and the weak intensity of the 111 peak makes it difficult to draw firm conclusions from those XRD patterns. For this reason, we now consider single-crystal diffraction.

### 3.4 Simulated Single-Crystal XRD Patterns

Single-crystal XRD provides another way of understanding the structural changes related to the superionic transition. Similarly to powder XRD, because it operates in the reciprocal space, it can reveal long-range features that are difficult to observe directly in simulation boxes due to thermal noise and increasing disorder at high temperatures. However, in contrast with powder XRD, there is no orientation averaging involved in the calculation. Hence, it is possible to precisely select definite regions of the reciprocal space to analyse. In order to be able to detect the appearance of superstructure peaks of the type 111, 113, etc., we computed the intensity distribution in (*HHL*) reciprocal lattice planes (that is, with a [1 $\bar{1}$ 0] zone axis). As for the powder diffraction study, we focus on BaF_2_ with the Sayle potential and we use Miller indices relative to the $Fm\bar{3}m$ structure even when discussing the *X* sublattice. Data showing the evolution of the RSMs of all investigated cases are available in the Supplementary Material.

**FIGURE 5:**
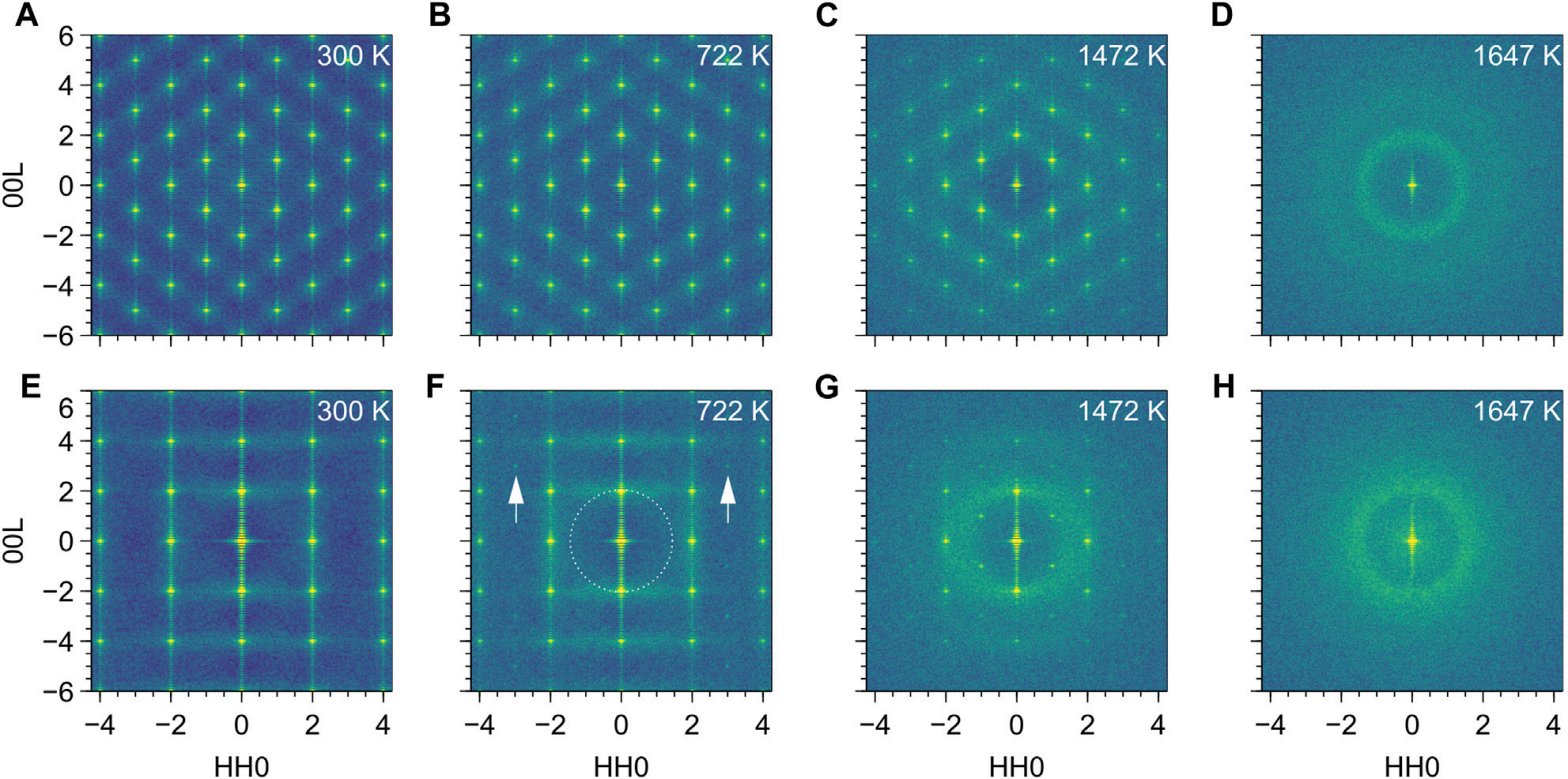
Simulated reciprocal space maps from MD simulations of BaF_2_ using the Sayle potential (Sayle et al., 2003): **(A–D)** Ba sublattice; **(E–H)** F sublattice. In **(F)** the arrows indicate the $\bar{11}3$ and 113 superstructure peaks, and the dotted line highlights the diffuse halo. The intensity is plotted on a logarithmic scale to enhance low-intensity details.

We first briefly discuss the RSMs obtained from the *A* sublattice shown in Figures 5A–D. From room temperature up to *T*_S_, besides diffuse scattering concentrated around the Bragg peaks, no significant changes were observed. The peak coordinates have here been corrected for thermal expansion to allow for an easier comparison between different temperatures. At *T*_M_, a diffuse scattering halo characteristic of an amorphous structure is observed pointing to the melting of the material (Figure 5D). These observations lead to two important conclusions. Firstly, apart from the observed thermal diffuse scattering, the fact that the signal from the *A* sublattice remains constant throughout the process - with no visible peak shift, peak broadening or splitting, or additional diffuse scattering—seems to indicate that the *A* sublattice is free of structural defects. This is in stark contrast with the *X* sublattice, which we will discuss shortly. We also know from direct analysis of the MD simulations shown in Section 3.2 that these defects are rare up until *T*_M_. Secondly, the thermal diffuse scattering forms streaks along the main ⟨111⟩ directions, indicating that atomic displacements are more pronounced in the close-packed {111} planes. This is also consistent with the weak and inhomogeneous diffuse scattering observed in the powder XRD data.

**FIGURE 6:**
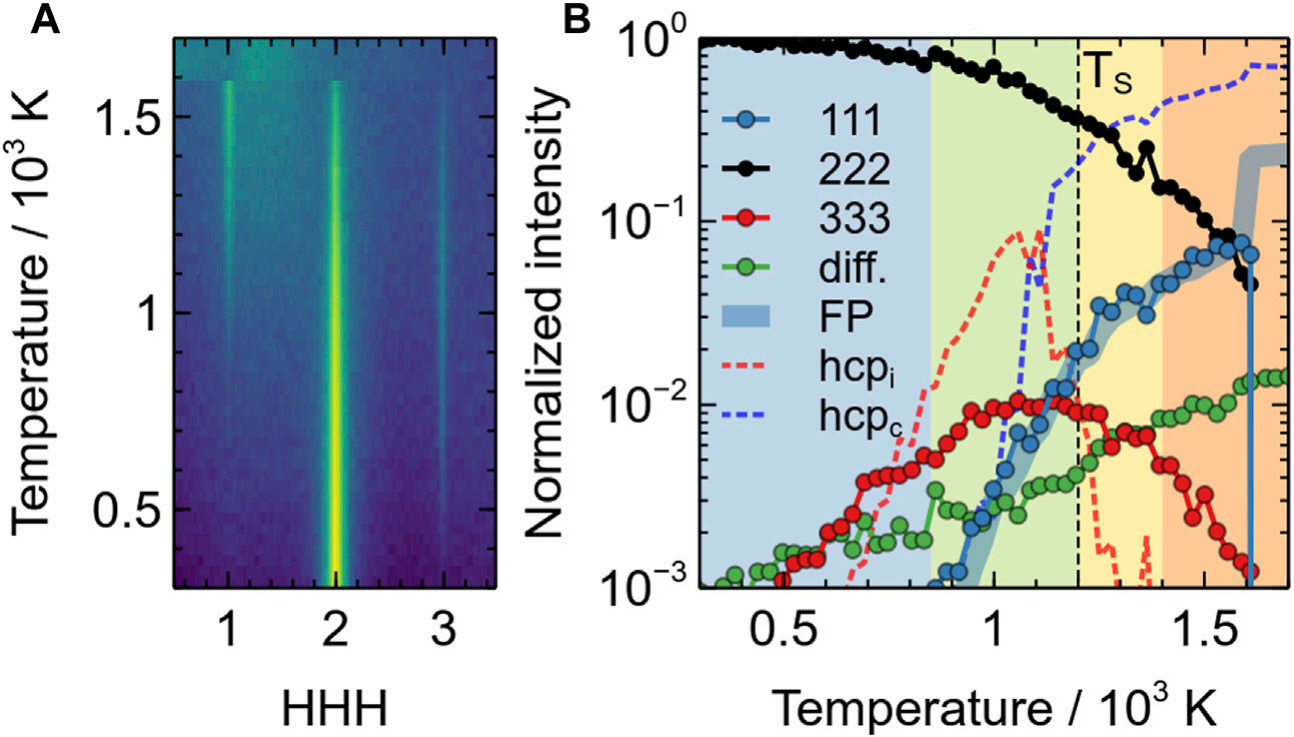
Evolution of the intensity of the additional single-crystal XRD peaks in the superionic phase of BaF_2_ using the Sayle potential: **(A)** evolution of the [111] intensity distribution as a function of temperature; **(B)** evolution of the 111 (blue circles), 222 (black circles), 333 (red circles) and diffuse scattering (green circles) peaks as a function of temperature. All intensities are on a logarithmic scale and normalised with respect to the room temperature of the 222 peak. Lines represent the evolutions of FP (thick blue), isolated HCP environments (red dotted) and clustered HCP environments (dotted blue). The different coloured areas correspond to the different regimes (i)–(iv) mentioned in the text.

As far as thermal diffuse scattering is concerned, similar conclusions can be drawn for the *X* sublattice shown in Figures 5E–H. Diffuse scattering streaks appear along the {001} and {110} planes of the SC structure, pointing to enhanced thermal motion in the corresponding lattice planes. However, as soon as the temperature increases, major changes are observed on the *X* sublattice. The first difference is the appearance of weak intensity high-order 33*L* superstructure peaks (for instance at 722 K, indicated by the arrows on Figure 5F). The second noticeable difference is the increase of the diffuse scattering connecting the Bragg peaks, together with the appearance of a weak diffuse scattering halo with [002] radius, indicated on the figure by a dotted circle. The halo is barely visible in the RSM in this case, but it can be definitely detected by analysing line scans performed along the [111] direction (see also Figure 6). The RSMs of all cases investigated are given in the Supplementary Material. All of them exhibit the diffuse halo appearing almost concomitantly with the 33*L* peaks with varying intensity. In the superionic phase (Figure 5G, representing a structure at 1,472 K), additional 11*L* superstructure peaks are formed and the diffuse halo is now clearly visible. It should be noted that the apparent ellipsoidal shape of the halo is due to the partial superposition of the halo with the diffuse streaks along [110] and [001]. The 33*L* peaks are still visible, but their intensity decreases because thermal motion starts affecting high angle reflections. Finally, all Bragg peaks disappear at *T*_M_. Only the diffuse halo remains then, characteristic of an amorphous structure, in this case of the molten phase. All the potentials showed both the additional peaks and the diffuse halo. However, the relative intensity of these features is potential- or material-dependent. For instance, all the potential for Li_2_O showed a particularly intense diffuse halo, which is explained by the fact that the light element Li is significantly affected by thermal motion. Some potentials also produced fainter 33*L* and 11*L* peaks than others. All these features are visible in the animations, which can be found in the Supplementary Material.

This evolution is summarised in Figure 6, which depicts the changes of the [111] line scans and the different intensities with temperature. There, it can be clearly observed that the diffuse halo starts to appear at a temperature slightly higher than room temperature (∼ 400–500 K, green circles), hence pointing to thermal vibrations. It then steadily increases with temperature. The 333 peak appears almost concomitantly (∼  500 K, red circles) and reaches a maximum close to *T*_S_. Around 850 K, the 111 peak starts to increase significantly (blue circles). This corresponds to the end of stage (i) and the beginning of stage (ii). The end of stage (ii), marking the superionic transition correspond to a temperature where the intensity of the 111 peak exhibit a change in slope and where the intensity of the 333 peak starts decreasing. Finally at *T*_M_, all intensities drop to 0 except the diffuse scattering, which remains constant.

This evolution is consistent with the powder XRD data, in particular the appearance of a 111 peak at *T*_S_ and a broad diffuse scattering background in *X*-only patterns. However, because of the orientation averaging inherent in powder XRD and the consecutive peak overlap, it is not possible to detect low intensity details as those discussed here. Finally, the fact that the diffuse scattering steadily increases until the melting temperature without any discontinuity, reinforces the description of the *X* substructure as a liquid made in the literature. In the next section, we discuss the origin of the superstructure peaks.

### 3.5 Structure of the Superionic Phase

**FIGURE 7:**
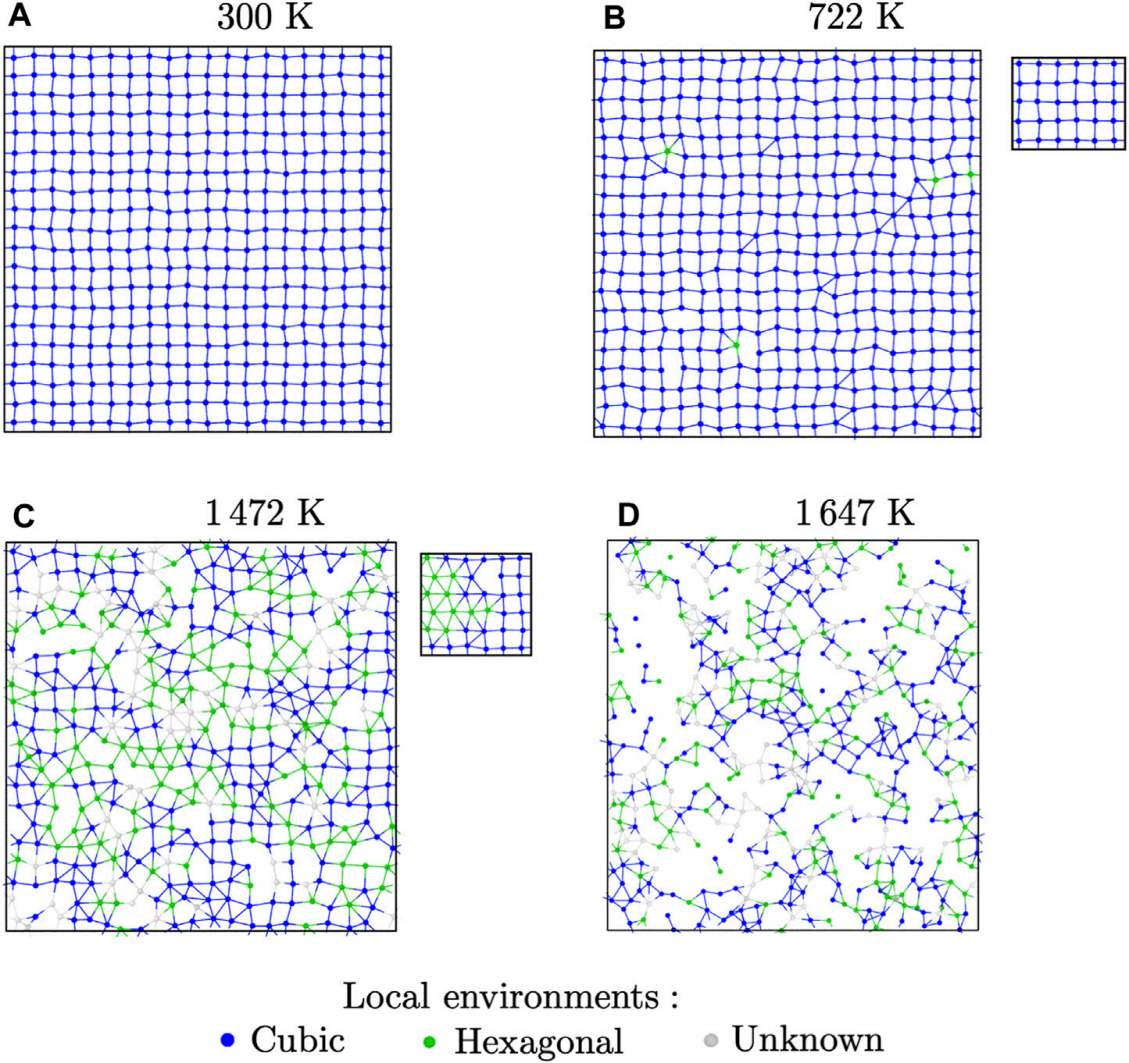
Slices showing (100) F atomic planes from MD relaxation of BaF_2_ with the Sayle potential at different temperatures, with Ba ions hidden for clarity: **(A)** room-temperature perfect crystal; **(B)** crystal with Frenkel defects; **(C)** superionic structure; **(D)** liquid. The colour code indicates the type of local environment according to the Polyhedral Template Matching analysis. The insets show the same slices after energy minimisation.

Visual examination of the structures during MD simulations at different temperatures reveals some structural changes. To highlight them, we extracted slices from supercells from MD simulations of BaF_2_ using the Sayle potential. The slices are 3 Å thick and centred on {100} atomic planes containing only F ions. At room temperature, the potential predicts a perfect $Fm\bar{3}m$ crystal. The slice at 300 K (Figure 7A) indeed shows a square structure, corresponding to the {100} faces of the cubes forming the *X* sublattice.

At intermediate temperatures, such as 722 K as in Figure 7B, some local distortions are visible, showing isolated triangular features. Visualisation of these features is helped by the use of the polyhedral template matching technique, which provides a characterisation of the local environment around each particle. At this temperature, almost all of the F ions are still in their ideal (SC) local environment. The closest matching structure for the ions with non-cubic local environments is HCP. The apparition of HCP local environments has been verified for all the potentials. With most of them, it coincides with the end of regime i and beginning of regime (ii). Most of the time, it also seemed to happen before Frenkel pairs were detected. However, this should be treated with caution. Indeed, the methods we used to count Frenkel pairs, though qualitatively accurate, can result in an under-estimation of the number of defects. On the other hand, the PTM analysis could also be too sensitive, resulting in false positives.

This changes around the superionic transition temperature *T*_S_. Indeed, in the superionic phase of regimes (iii) and (iv), we could see some clustering of the atoms with HCP environments, which were much more likely to be first neighbours. This is shown in Figure 7C, where we can see clusters of HCP environments mixed with cubic regions. This pattern holds until the mechanical melting point *T*_M_, after which the structure becomes liquid (Figure 7D).

The ions with unknown local environments are cases where the PTM algorithm could not decide how to categorise the particle. There are very few of them below *T*_S_. For example, in the case of BaF_2_ with the Sayle potential, they represent 5% of all F ions at the superionic transition. They are more prevalent in the superionic phase, but still amount to 13% at the mechanical melting point. They are much more present in the liquid phase, where the structure is not well-suited to the PTM analysis. These ions with disordered local environments are expected to contribute to the diffuse scattering observed in both powder and single-crystal diffraction patterns. The MD simulation boxes in the superionic phases also show that there can be a high degree of misorientation between the different types of local environments. This also contributes to the diffuse scattering.

This evolution of the number of ions with HCP local environments is plotted in Figure 6, together with the XRD data. This figure clearly confirms the findings described previously. Isolated HCP environments appear at relatively low temperatures (∼  600 K), well before Frenkel pairs. The concentration of those isolated HCP environments, noted hcp_i_, are neatly correlated with the apparition of the 33*L* superstructure peaks, which indicate that these defects reduce the unit-cell symmetry to a period of $∼\frac{1}{3}$ of the (111) fluorite lattice spacing.

In terms of temperature range, the fraction of clustered HCP environments, noted hcp_c_, is almost perfectly correlated both with the fraction of Frenkel pairs, and the intensity of the 111 peak. This indicates that the Frenkel pairs are at the origin of (i) the clustering of HCP environments, and (ii) the formation of ordered local environments on the *X* sublattice, with a periodicity equal to that of a fluorite unit-cell.

The PTM analysis in itself does not give a complete picture of the local environments it detects. For example, the actual local environments also depend on the position of neighbours on the *A* sublattice. Those were completely ignored in our PTM analysis, which was done separately for each sublattice. Determination of the local environments directly from MD simulations proved all but impossible due to distortions and large thermal fluctuations in the superionic phases. To work around this, we performed constant-volume geometry optimisations from the final structure of the MD simulations for each potential and temperature. During this operation, the ions were moved towards more energetically favorable positions, thus removing thermal noise from vibrations and unstable defects. The results were structures that were easier to interpret and characterise. It should be noted, however, that the energy-minimised and high-temperature structures are not the same. Indeed, some temperature-activated phenomena disappear during geometry optimisation. Thus, the energy-minimised structure can only provide some clues as to how to interpret high-temperature MD simulations.

The structures optimised from high-temperature simulations showed both cubic and non-cubic local environments after PTM analysis. The trend was also similar to what was observed in high-temperature simulations. In regime i, all potentials produced a perfect $Fm\bar{3}m$ crystal after optimisation. This changed in regime (ii), where we could observe some isolated ions with non-cubic environments, as well as some trapped Frenkel pairs, which could not recombine during energy minimisation. Above the superionic transition temperature, most potentials showed a separation into two phases, which contained the cubic and non-cubic environments respectively. Visual examination of the non-cubic phase showed the same triangular pattern observed in MD simulations, except that the pattern covered extensive regions of the simulation box instead of small, elongated clusters. This is visible in the inset in Figure 7C. In addition, because all thermal noise was removed, we could isolate the non-cubic environment for a more thorough characterisation. We found that the structure we isolated had the symmetry elements of the *Pbcn* space group. This space group corresponds to the scrutinyite structure (*α*-PbO_2_), and is well-known in some compounds of the *AX*_2_ form. In particular, there are some instances of such compounds undergoing a pressure-induced phase transition to distorted fluorite structures Léger and Haines (1997); López-Moreno et al. (2016). A transition from $Fm\bar{3}m$ to *Pbcn* hasn’t been documented in materials with the fluorite structure, however, except for UO_2_, where it was seen during fracture (Zhang et al. (2012)) or under tensile mechanical loading (Fossati et al. (2013)). Even in that case, the *Pbcn* phase was not thermodynamically stable.

**FIGURE 8:**
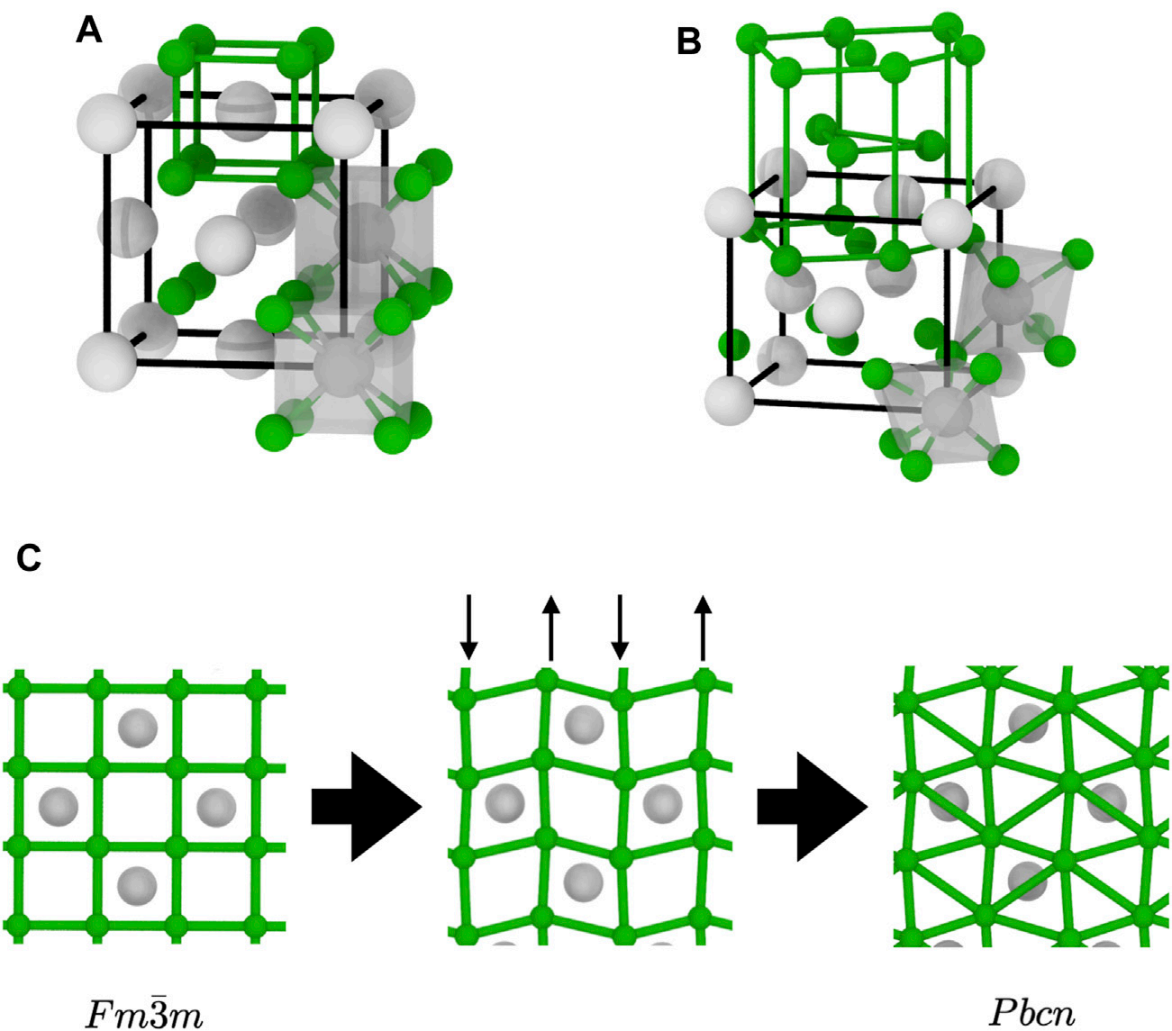
Structural transition between the two *AX*_2_ structures relevant to this study: **(A)** $Fm\bar{3}m$ ; **(B)** *Pbcn*; and **(C)** deformation of a [100] atomic plane during the transition, with the vertical arrows indicating the displacement of the columns of *C* ions. The bonds emphasise the structure of the *X* sublattice: simple cubic in $Fm\bar{3}m$ and distorted hexagonal close packed (HCP) in *Pbcn*. The polyhedra show the coordination environments of the *A* ions: cubic in $Fm\bar{3}m$ and octahedral in *Pbcn*. Subfigures on the bottom show the changes in a [010] plane during the transition and the appearance of the triangular patterns in the basal planes of the HCP substructure.

In the *Pbcn* structure, the *X* sublattice has a distorted HCP structure, whilst the *A* sublattice remains close to FCC, as shown in Figure 8B. The pattern shown in the (0001) basal plane of the *X* sublattice in that structure, shown in Figure 8, is the triangular pattern visible in both MD simulations and energy-minimised structures. Thus, we can conclude that the non-cubic local environments detected by the PTM analysis are actually *X* ions which have a local environment consistent with that of the *Pbcn* structure. The overall structure of the superionic phase, however, is quite different from what was observed during the $Fm\bar{3}m\to Pbcn$ transition (Fossati et al., 2013). Indeed, it does not consist in a clear separation between a $Fm\bar{3}m$ and *Pbcn* phases. It is formed instead of a mixture of local environments with different structures, along with some ions with a disordered environment. Moreover, we can see on Figure 7C that there can be a large degree of disorientation between different local environments.

We can now propose a mechanism for the superionic transition in materials with the fluorite structure. Upon heating, the *X* sublattice is subject to thermal vibrations compatible the with symmetry of the crystal. This gives rise to the diffuse scattering in the {001} planes observed in the RSMs of the *X* sublattice. There is a known vibration mode that fits this description. It is the *B*_1*u*_ mode with a [0 0 $\frac{1}{2}$ ] wavevector, which has been linked to the superionic transition in CeO_2_ (Klarbring et al., 2018). The polarisation vector of this mode corresponds exactly to the displacement of the *X* ions at the beginning of the $Fm\bar{3}m\to Pbcn$ transition shown in Figure 8C. These distortions result in the appearance of local environments with HCP symmetry elements, initially isolated and distributed in the $Fm\bar{3}m$ structure. Upon further heating, the crystal starts forming *X* Frenkel pairs. This coincides with the clustering of isolated HCP local environments, with symmetry elements characteristic of the *Pbcn* structure, throughout the crystal. These local environments are responsible for the anomalous enthalpy, and the exponential increase in the heat capacity leading to the Λ peak. The structure change could also be the reason of the different diffusion mechanism in the superionic phase. In contrast, the volume fraction of the disordered (liquid- or amorphous-like structure), responsible for the diffuse scattering halo, steadily increases from room temperature up to melting without any noticeable change. It is hence unlikely to be involved in the drastic changes observed in the thermodynamic properties.

## 4 Conclusion

We used a wide selection of empirical potentials to simulate several different compounds with the fluorite structure and investigate structural changes between the low-temperature perfect structure and that of the superionic phase.

Although a full validation of each potential was not done here, we verified that the properties they predict are realistic by comparing them to available experimental values. In particular, the superionic transition temperatures, the enthalpies of formation for the *X* Frenkel pairs, and the activation energies for *X* diffusion in both the $Fm\bar{3}m$ and superionic phases were found to be adequate. Therefore, whilst the potentials are quantitatively imperfect when taken separately, we are confident that the qualitative trends they show are not potential dependent.

From a thermodynamical point of view, not all the potentials reproduced the characteristic *C*_P_ and *α* peaks usually associated with the superionic transition, despite showing other aspects such as structural disorder and a change in the diffusion properties. This demonstrates that the transition could occur without a clearly-visible peak. Different potentials for the same compound could also exhibit different behaviour. For example, in BaF_2_, the Catlow potential showed a sharp Λ peak in its *C*_P_(*T*) curve, whereas the Sayle potentials showed a broad plateau, and the transition was almost invisible from the thermodynamical properties of the Cazorla potential.

All potentials are characterised by a 2-regime diffusion behaviour, with a change in the apparent activation energy at the superionic transition consistent with available experimental data. Moreover, populations of *X* Frenkel pairs showed a similar trend, with a low-temperature Arrhenius regime followed by a progressive transition towards a lower apparent formation energy in the superionic phases. This is consistent with a transition from a diffusion process mediated by isolated point defects in low-temperature crystals to a collective process involving clusters in the superionic phases.

Powder and single-crystal XRD patterns were calculated from the MD simulation boxes for investigate structural changes from the reciprocal space. Whilst the *A* sublattices did not change from room temperature to the melting point, additional Bragg peaks on the *X* sublattices showed the emergence of new structural features distinct from the simple partially molten structure sometimes assumed. This observation could not be made using experiments, which could not have separated signals from both sublattices. Subsequent analysis of the MD simulation boxes could determine that the additional peaks are the result from a the presence of local environments with a structure close to the *Pbcn* structure. In these local environments, the *X* sublattice has HCP features. This work is a demonstration of the power of combined MD and computational diffraction techniques to described structures associated with widespread disorder.

Following our observations, we can describe the evolution with temperature of the structure of the fluorite compounds, following the four stages defined from thermodynamical properties and outlined in Section 3.1. Only the *X* sublattice is discussed in the following: the *A* sublattice remains FCC from room temperature to *T*_M_, with some distortions at high temperatures.

### Stage (i)

This is the low-temperature regime, in which the heat capacity varies slowly as a polynomial. Near the end of this temperature range, isolated hexagonal local environments are formed in an otherwise perfect $Fm\bar{3}m$ matrix. This stage is the extrinsic diffusion regime. No diffusion could be measured from the MD simulations because for their short time scale and lack of extrinsic defects.

### Stage (ii)

In this regime, *C*_P_(*T*) follows an exponential increase leading to the top of the Λ peak, or the left-hand edge of the plateau depending on the potential. The end of this stage is *T*_S_ and the onset of the superionic transition. The structure is characterised by the accumulation of Frenkel pairs and hexagonal environments becoming more present. The number of isolated HCP environments peaks near the end of this stage. This corresponds to the intrinsic diffusion regime, limited by Frenkel pairs, where diffusion coefficients follow an Arrhenius law.

### Stage (iii)

In this transition stage, the *C*_P_(*T*) curve decreases sharply, forming the high-temperature side of the Λ peak. The number of isolated hexagonal environments also decreases, to become negligible by the end of this stage, at which point all the hexagonal local environments are part of clusters. The beginning of this stage is associated with a change in slope of the Frenkel pair concentration, diffusion coefficient, and the concentration of clustered hexagonal local environments, which all increase at a reduced rate. The diffusion coefficients progressively change from the stage (ii) to the stage iv Arrhenius regimes. This stage might not be visible if the potential does not predict a *C*_P_ peak.

### Stage (iv)

The growth of the *C*_P_(*T*) curve resumes slowly, leading to the mechanical melting at *T*_M_. The structure is characterised by a mixture of regions with hexagonal local environments, and regions that retain the cubic local environment of the perfect fluorite structure. The *X* diffusion coefficient follows another Arrhenius law, with an activation energy smaller than that of stage (ii), underlying the collective diffusion mechanism underlines elsewhere (Annamareddy et al., 2014).

## Data Availability

The raw data supporting the conclusions of this article will be made available by the authors, without undue reservation.
