# Supplementary material for: Structural Aspects of the Superionic Transition in AX2 Compounds With the Fluorite Structure
Source: Front Chem. 2021 Oct 18;9:723507. doi: 10.3389/fchem.2021.723507 (PMC8558309; doi:10.3389/fchem.2021.723507)
Supplement: Supplementary file 1 [file DataSheet2.pdf]

Supplementary Material — Structural aspects of the superionic transition in AX_2 compounds with the fluorite structure

ANIMATED FIGURES

The documents named video1.mp4 to video18.mp4 contain animated versions of the figure 5 of the main article. They show the evolution with temperature of reciprocal space maps (RSM) calculated from molecular dynamics simulations of different compounds with the fluorite structure. In each video, the maps on the left show the cation sublattice and those on the right show the anion sublattice. Each video corresponds to one of the compounds and potentials discussed in the main article, to which the reader is referred to for more information.

- Video1.mp4: BaF_2 with the Catlow potential
- Video2.mp4: BaF_2 with the Cazorla potential
- Video3.mp4: BaF_2 with the Sayle potential
- Video4.mp4: CaF_2 with the Bingham potential
- Video5.mp4: CaF_2 with the Catlow potential
- Video6.mp4: CaF_2 with the Evangelakis potential
- Video7.mp4: CaF_2 with the Sayle potential
- Video8.mp4: Li_2O with the Asahi potential
- Video9.mp4: Li_2O with the Oda potential
- Video10.mp4: Li_2O with the Pedone potential
- Video11.mp4: α–PbF_2 with the Catlow potential
- Video12.mp4: $SrCl_2$ with the Bendall potential
- Video13.mp4: $SrCl_2$ with the Gillan potential
- Video14.mp4: SrF_2 with the Bingham potential
- Video15.mp4: SrF_2 with the Catlow potential
- Video16.mp4: SrF_2 with the Cazorla potential
- Video17.mp4: UO_2 with the CRG (Cooper) potential
- Video18.mp4: UO_2 with the Morelon potential
