# Supplementary material for: Structural Aspects of the Superionic Transition in AX2 Compounds With the Fluorite Structure
Source: Front Chem. 2021 Oct 18;9:723507. doi: 10.3389/fchem.2021.723507 (PMC8558309; doi:10.3389/fchem.2021.723507)
Supplement: Supplementary file 7 [file DataSheet1.PDF]

# *Supplementary Material — Structural aspects of the superionic transition in $AX_2$ compounds with the fluorite structure*

## 1 THERMODYNAMICAL PROPERTIES

The main article contains a discussion of thermodynamical and defects properties of fluorite compounds simulated with empirical potentials. For the sake of conciseness, only 2 potentials are explicitly shown in figure 2. This document contains the equivalent figures for the other potentials. There are 6 figures for each potential.

The first 4 show the thermodynamical parameters $H$, $C_{\mathrm{P}}$, $a$, and $\alpha$. For each potential and temperature, these properties were measured by averaging 3 independent simulations. The points on these figures show results from individual simulations, whilst the solid black line is the average. The dashed lines show fits to the low-temperature, polynomial regime (regime *i* in the main article) and the exponential increase in both enthalpy and heat capacity that leads to the superionic transition (regime *ii*).

The last two figures for each potential show the diffusion coefficient and point defect fractions. The dashed line in the defects fraction figures shows a fit to the low-temperature Frenkel pair concentration, which is the equation (5) of the main article. The dashed and dotted lines in the diffusion coefficient curves show fits to Arrhenius exponential functions, respectively below and above the superionic transition.

The reader is referred to the main article for any other technical detail and for the full discussion.

## 1.1 $BaF_2$ – Catlow

| | |
|---|---|
| Superionic transition temperature: | $T_S$ = 1540 K |
| Mechanical melting point: | $T_M$ = 2293 K |
| Crossover temperature: | $T_C$ = 1615 K |
| Frenkel pair formation enthalpy: | $H_f$ = 3.666 eV |
| Diffusion activation energy in the crystal phase: | $E_a^c$ = 2.903 eV |
| Diffusion activation energy in the superionic phase: | $E_a^s$ = 0.626 eV |

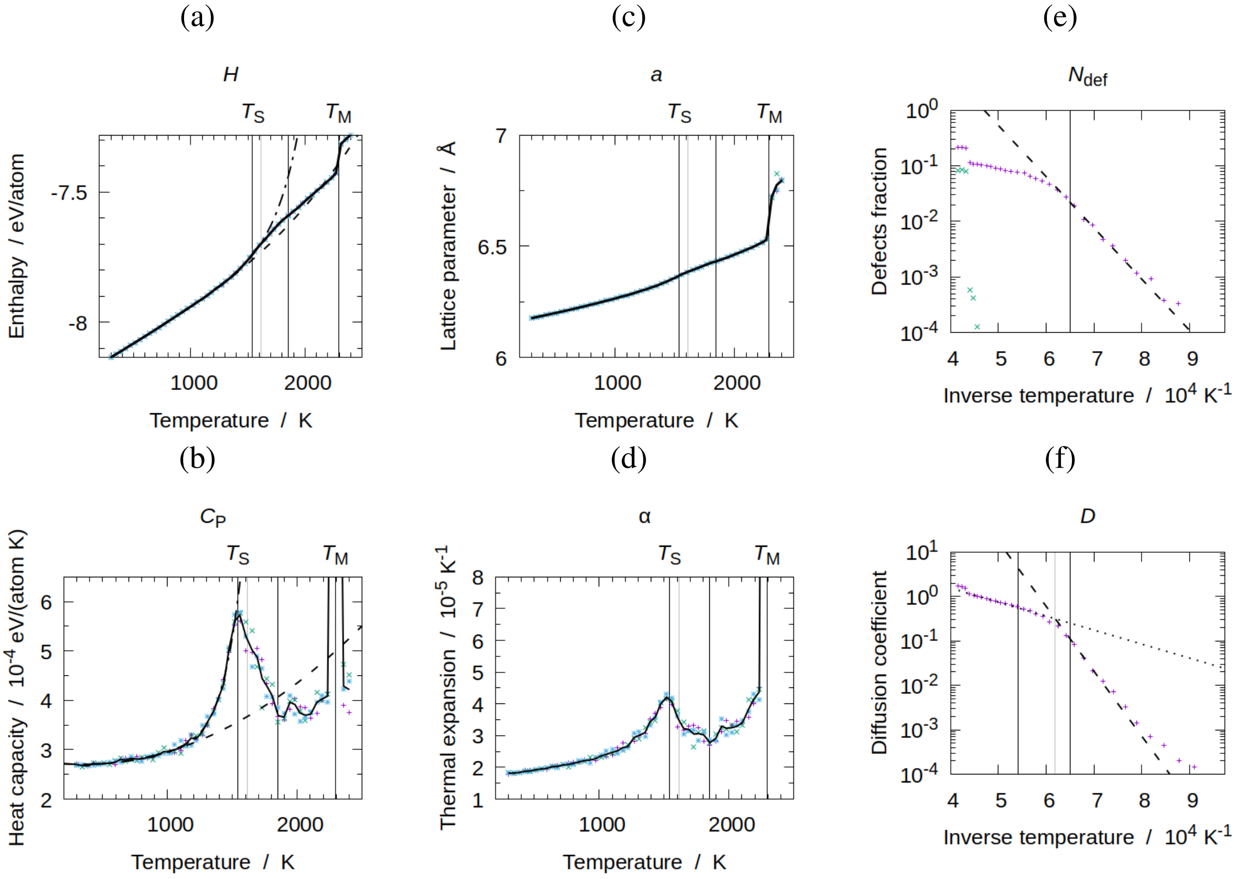


Figure S1: Thermodynamical properties from MD simulations of $BaF_2$ using the Catlow potential.

## 1.2 $BaF_2$ – Cazorla

| | |
|---|---|
| Superionic transition temperature: | $T_S$ = 1200 K |
| Mechanical melting point: | $T_M$ = 1631 K |
| Crossover temperature: | $T_C$ = 1405 K |
| Frenkel pair formation enthalpy: | $H_f$ = 2.069 eV |
| Diffusion activation energy in the crystal phase: | $E_a^c$ = 1.155 eV |
| Diffusion activation energy in the superionic phase: | $E_a^s$ = 1.044 eV |

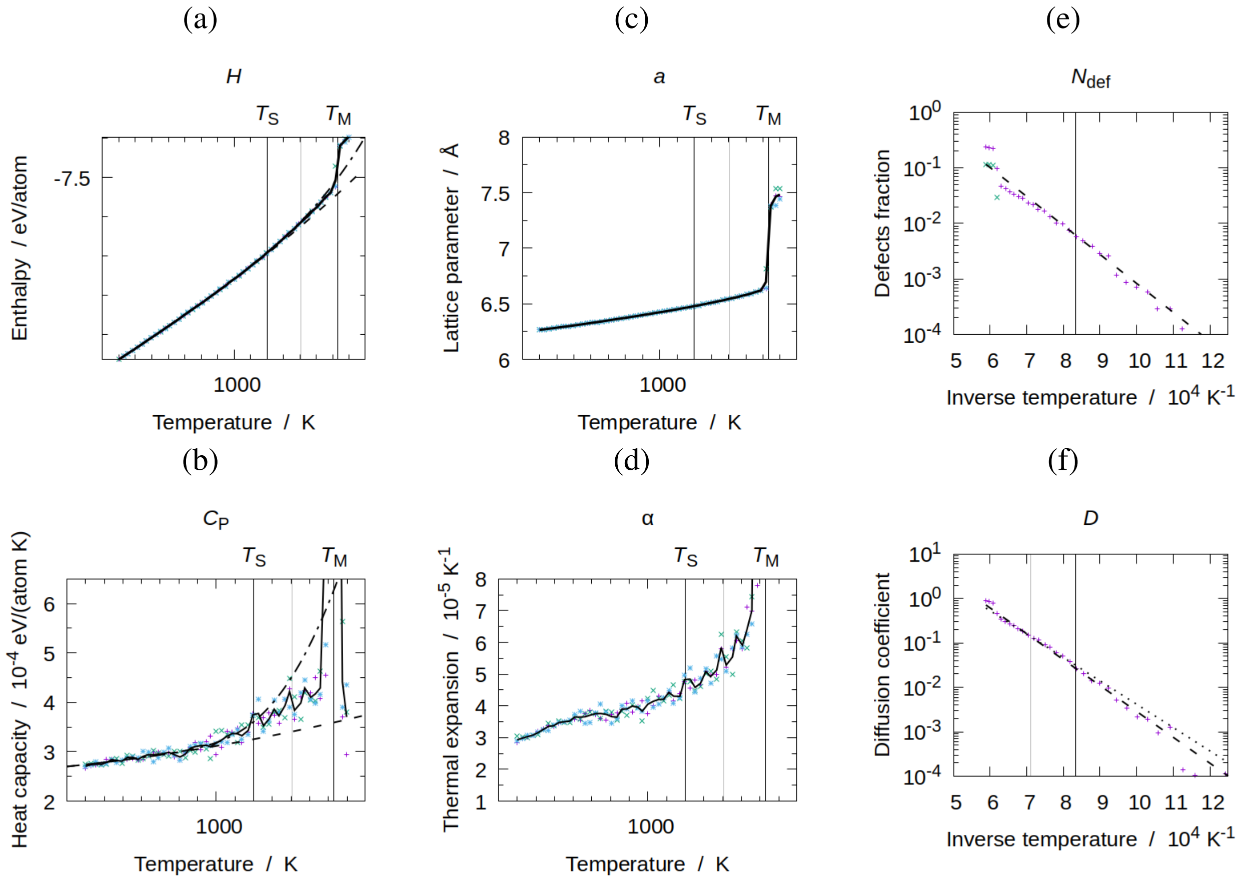


Figure S2: Thermodynamical properties from MD simulations of $BaF_2$ using the Cazorla potential.

## 1.3 $BaF_2$ – Sayle

| | |
|---|---|
| Superionic transition temperature: | $T_S$ = 1200 K |
| Mechanical melting point: | $T_M$ = 1599 K |
| Crossover temperature: | $T_C$ = 1253 K |
| Frenkel pair formation enthalpy: | $H_f$ = 2.013 eV |
| Diffusion activation energy in the crystal phase: | $E_a^c$ = 1.301 eV |
| Diffusion activation energy in the superionic phase: | $E_a^s$ = 0.683 eV |

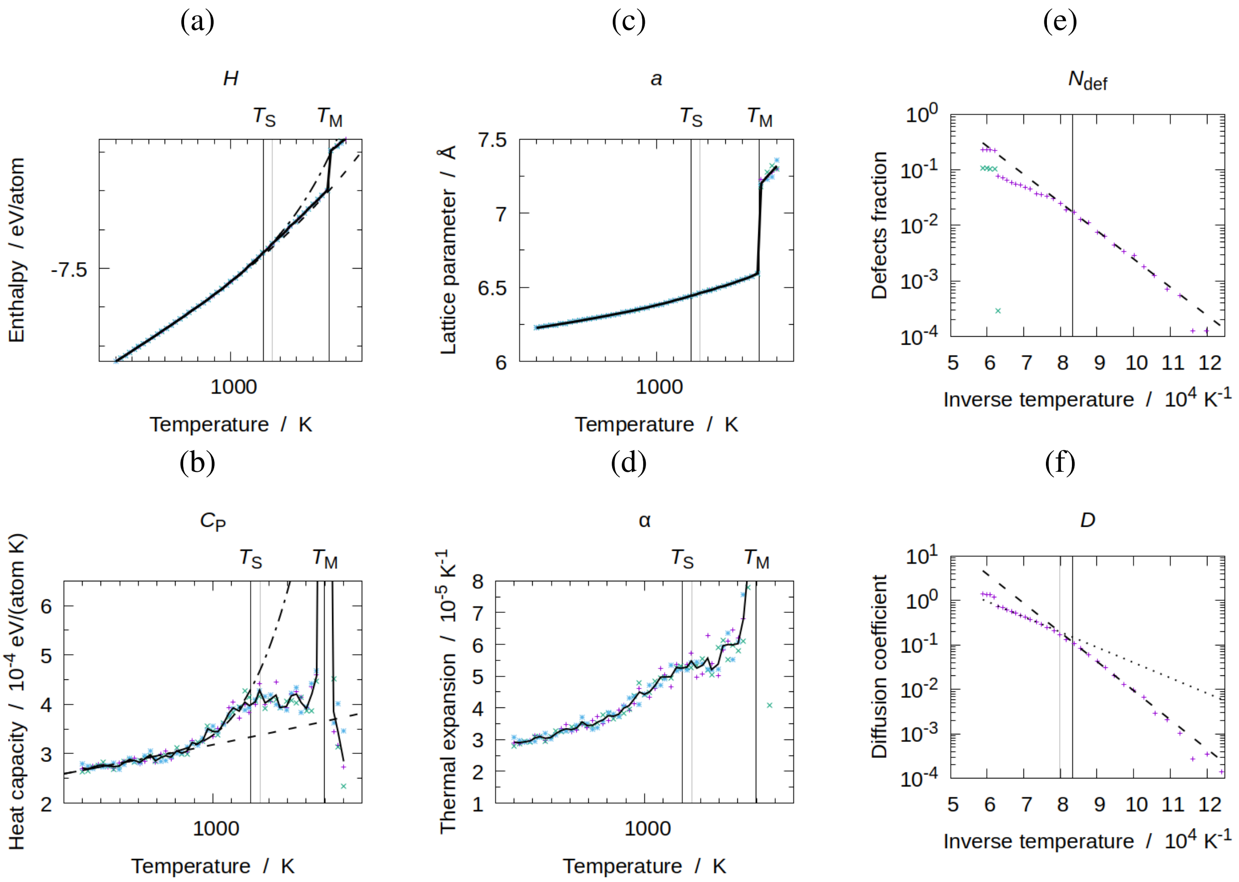


Figure S3: Thermodynamical properties from MD simulations of $BaF_2$ using the Sayle potential.

## 1.4 $CaF_2$ – Bingham

| | |
|---|---|
| Superionic transition temperature: | $T_S$ = 1300 K |
| Mechanical melting point: | $T_M$ = 1913 K |
| Crossover temperature: | $T_C$ = 1431 K |
| Frenkel pair formation enthalpy: | $H_f$ = 2.579 eV |
| Diffusion activation energy in the crystal phase: | $E_a^c$ = 1.523 eV |
| Diffusion activation energy in the superionic phase: | $E_a^s$ = 0.821 eV |

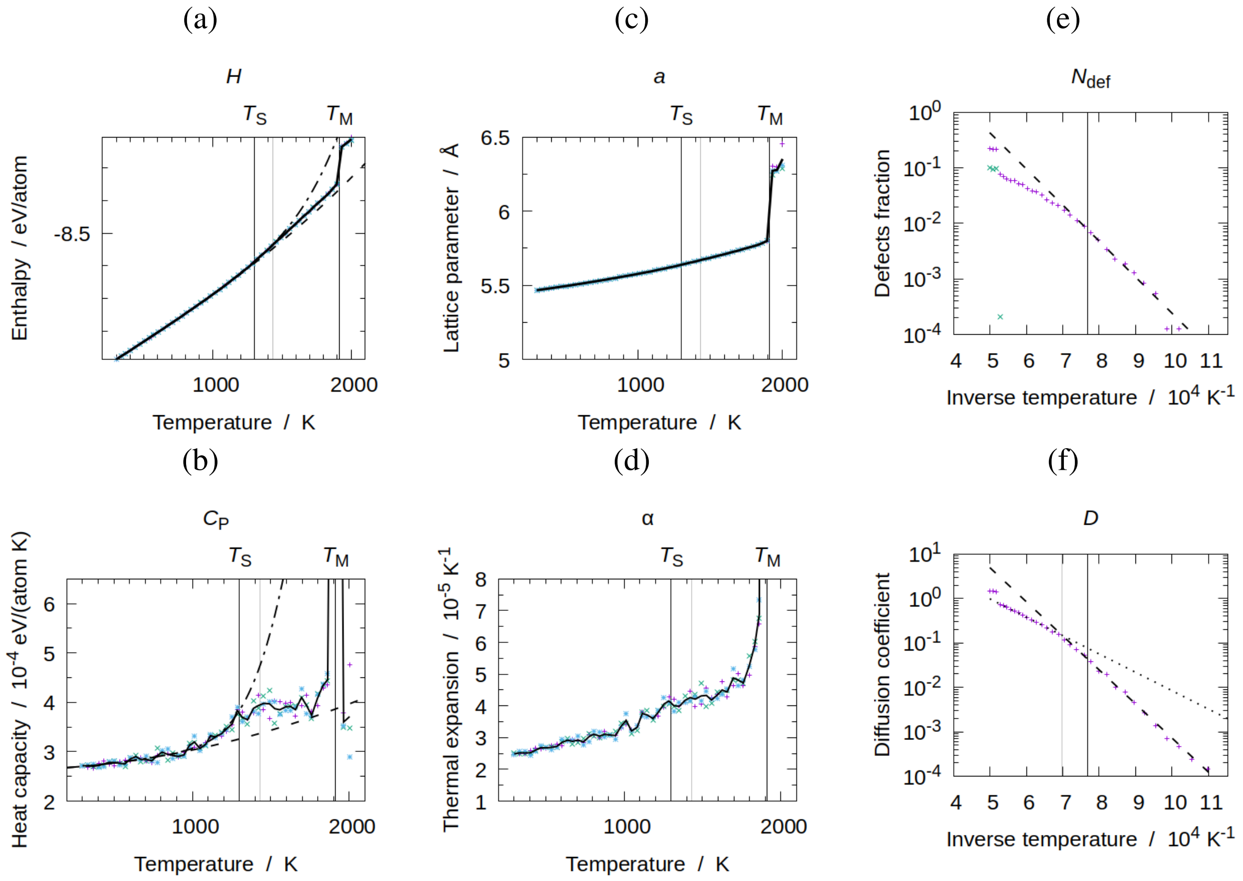


Figure S4: Thermodynamical properties from MD simulations of $CaF_2$ using the Bingham potential.

## 1.5 $CaF_2$ – Catlow

| | |
|---|---|
| Superionic transition temperature: | $T_S$ = 1650 K |
| Mechanical melting point: | $T_M$ = 2673 K |
| Crossover temperature: | $T_C$ = 1722 K |
| Frenkel pair formation enthalpy: | $H_f$ = 3.171 eV |
| Diffusion activation energy in the crystal phase: | $E_a^c$ = 1.989 eV |
| Diffusion activation energy in the superionic phase: | $E_a^s$ = 0.576 eV |

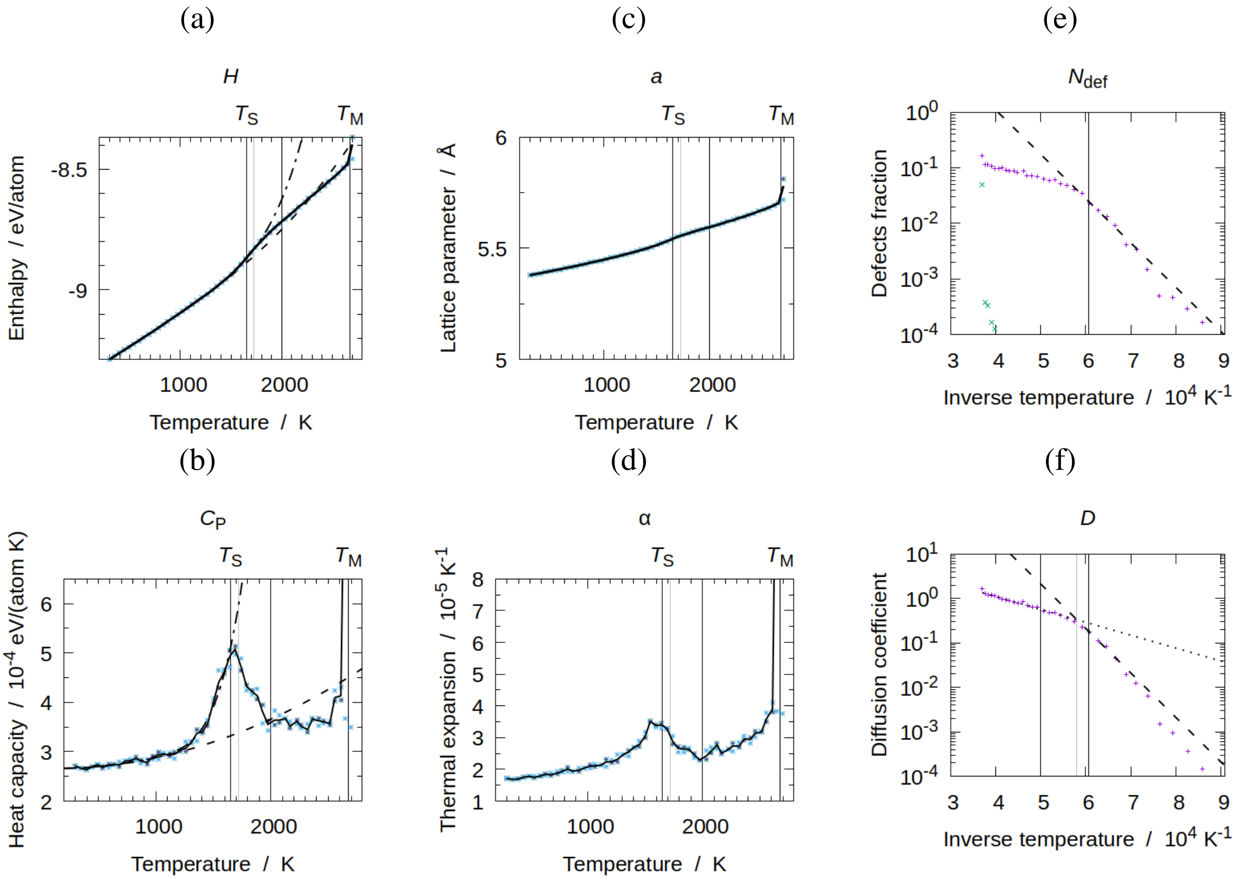


Figure S5: Thermodynamical properties from MD simulations of $CaF_2$ using the Catlow potential.

## 1.6 $CaF_2$ – Evangelakis

| | |
|---|---|
| Superionic transition temperature: | $T_S = 1400$ K |
| Mechanical melting point: | $T_M = 2434$ K |
| Crossover temperature: | $T_C = 1553$ K |
| Frenkel pair formation enthalpy: | $H_f = 3.318$ eV |
| Diffusion activation energy in the crystal phase: | $E_a^c = 2.122$ eV |
| Diffusion activation energy in the superionic phase: | $E_a^s = 0.488$ eV |

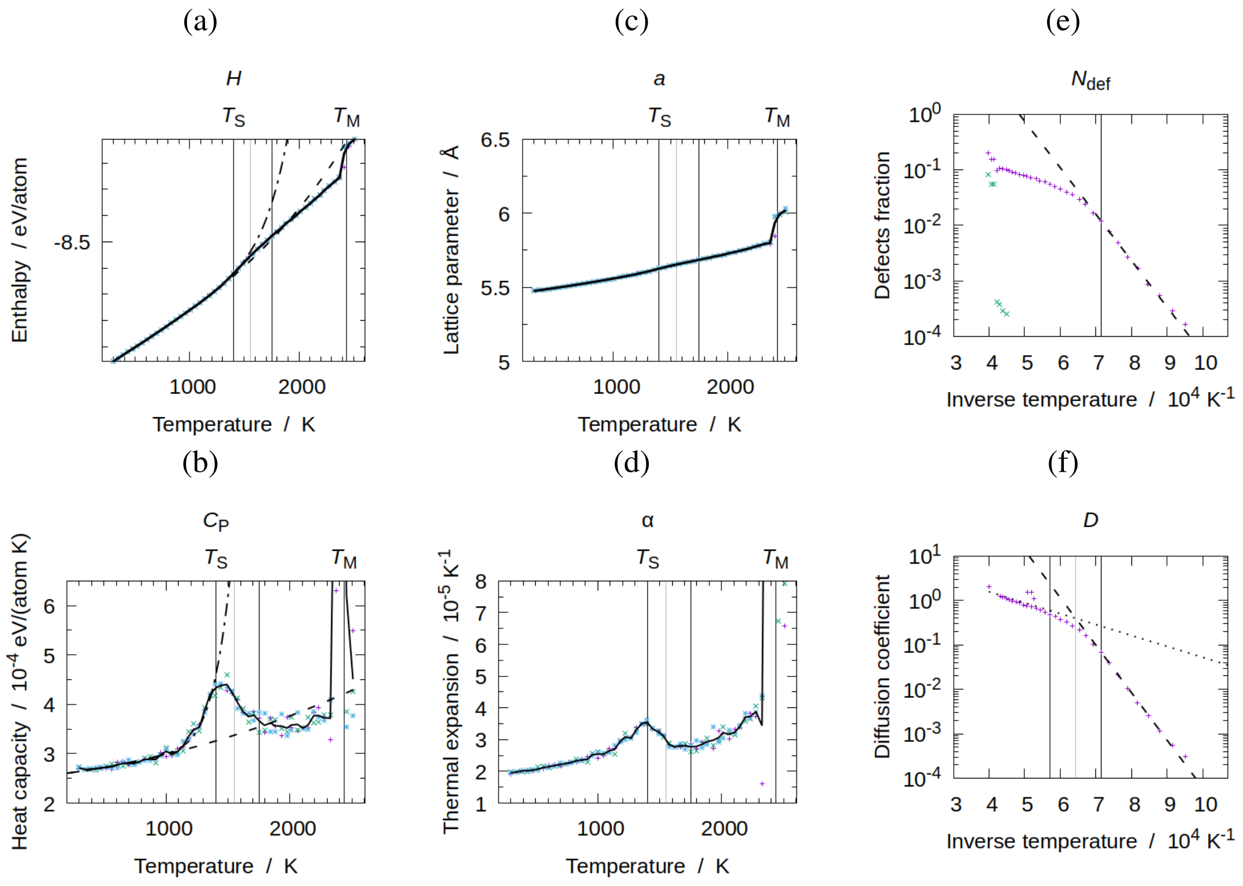


Figure S6: Thermodynamical properties from MD simulations of $CaF_2$ using the Evangelakis potential.

## 1.7 $CaF_2$ – Sayle

| | |
|---|---|
| Superionic transition temperature: | $T_S$ = 1300 K |
| Mechanical melting point: | $T_M$ = 1983 K |
| Crossover temperature: | $T_C$ = 1476 K |
| Frenkel pair formation enthalpy: | $H_f$ = 2.514 eV |
| Diffusion activation energy in the crystal phase: | $E_a^c$ = 1.628 eV |
| Diffusion activation energy in the superionic phase: | $E_a^s$ = 0.817 eV |

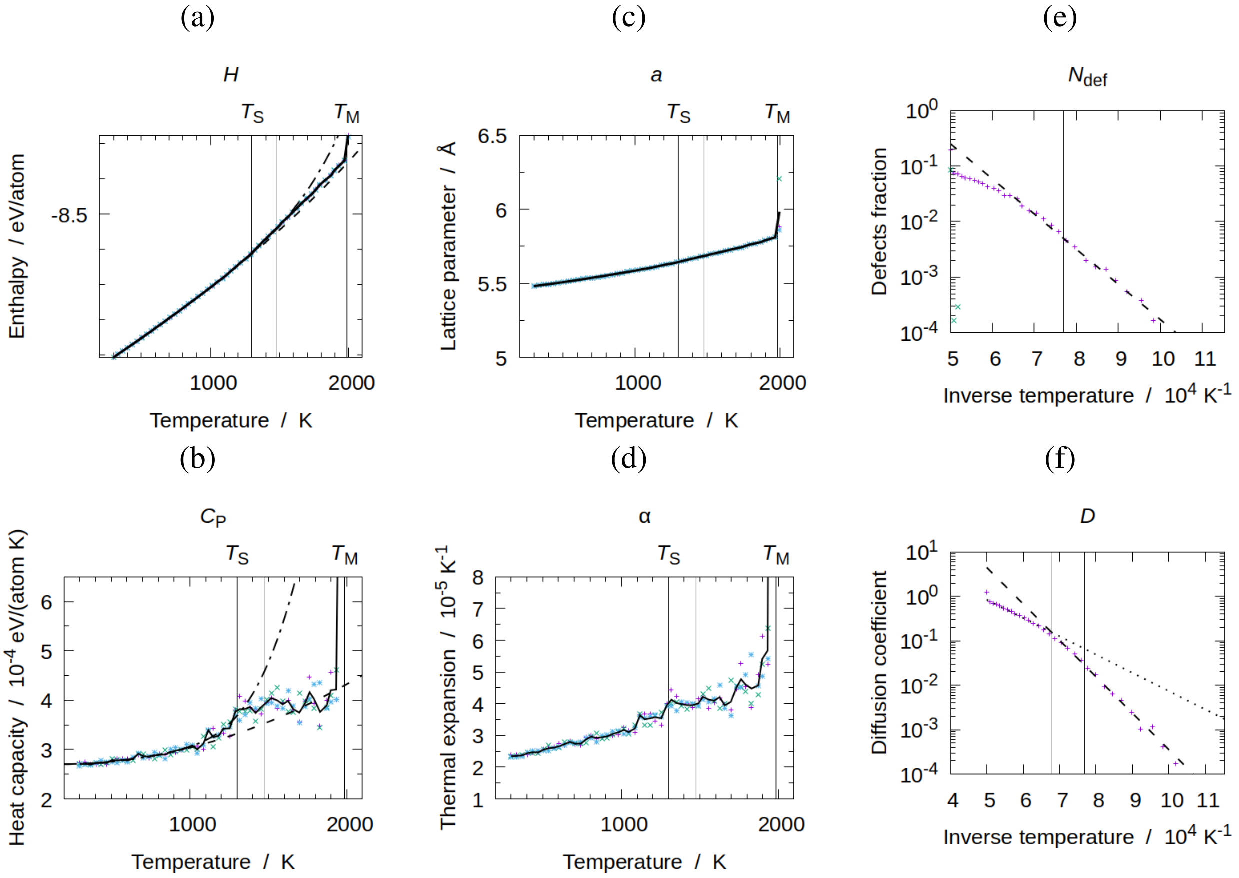


Figure S7: Thermodynamical properties from MD simulations of $CaF_2$ using the Sayle potential.

## 1.8 $Li_2O$ – Asahi

| | |
|---|---|
| Superionic transition temperature: | $T_S = 1275$ K |
| Mechanical melting point: | $T_M = 1913$ K |
| Crossover temperature: | $T_C = 1369$ K |
| Frenkel pair formation enthalpy: | $H_f = 2.977$ eV |
| Diffusion activation energy in the crystal phase: | $E_a^c = 1.968$ eV |
| Diffusion activation energy in the superionic phase: | $E_a^s = 0.584$ eV |

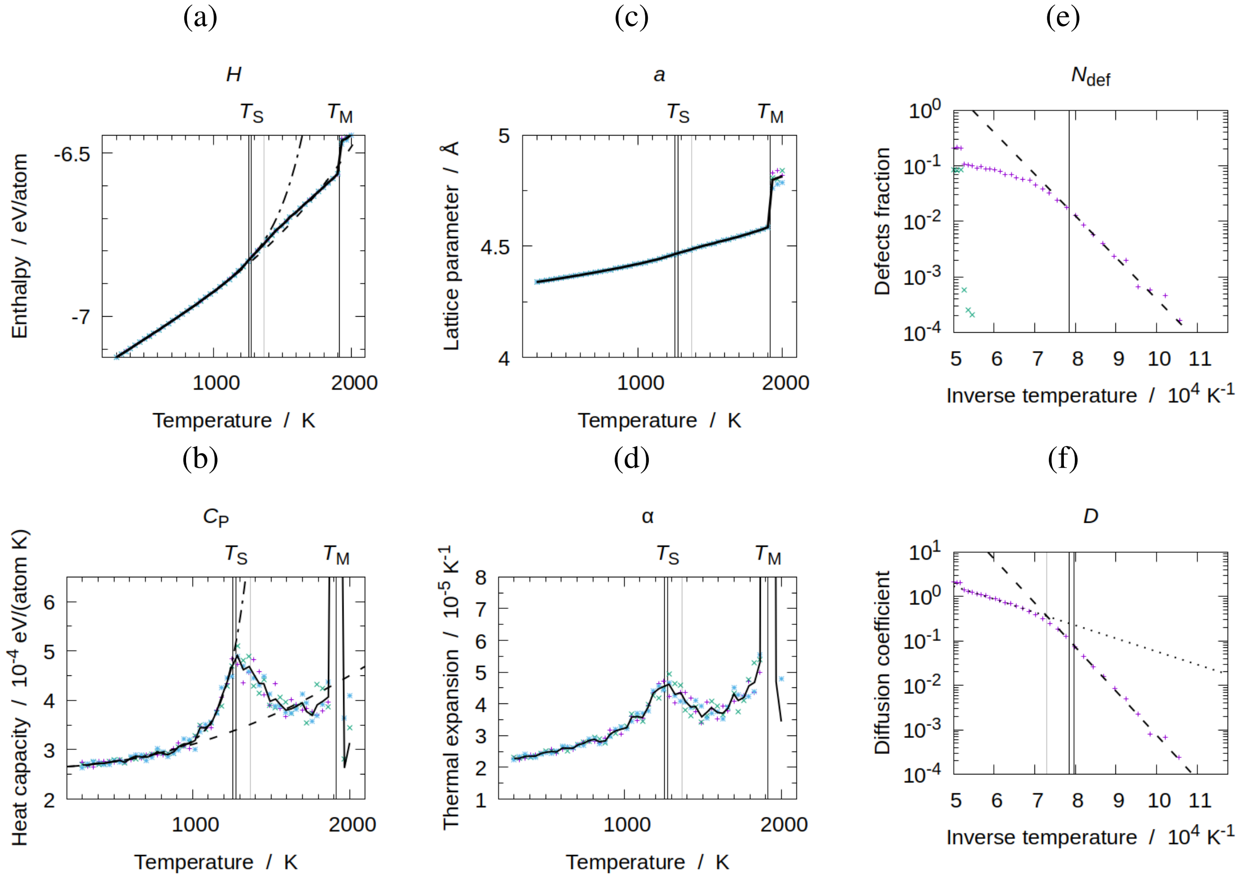


Figure S8: Thermodynamical properties from MD simulations of $Li_2O$ using the Asahi potential.

## 1.9 $Li_2O$ – Oda

| | |
|---|---|
| Superionic transition temperature: | $T_S = 1300$ K |
| Mechanical melting point: | $T_M = 1947$ K |
| Crossover temperature: | $T_C = 1382$ K |
| Frenkel pair formation enthalpy: | $H_f = 2.299$ eV |
| Diffusion activation energy in the crystal phase: | $E_a^c = 1.674$ eV |
| Diffusion activation energy in the superionic phase: | $E_a^s = 0.593$ eV |

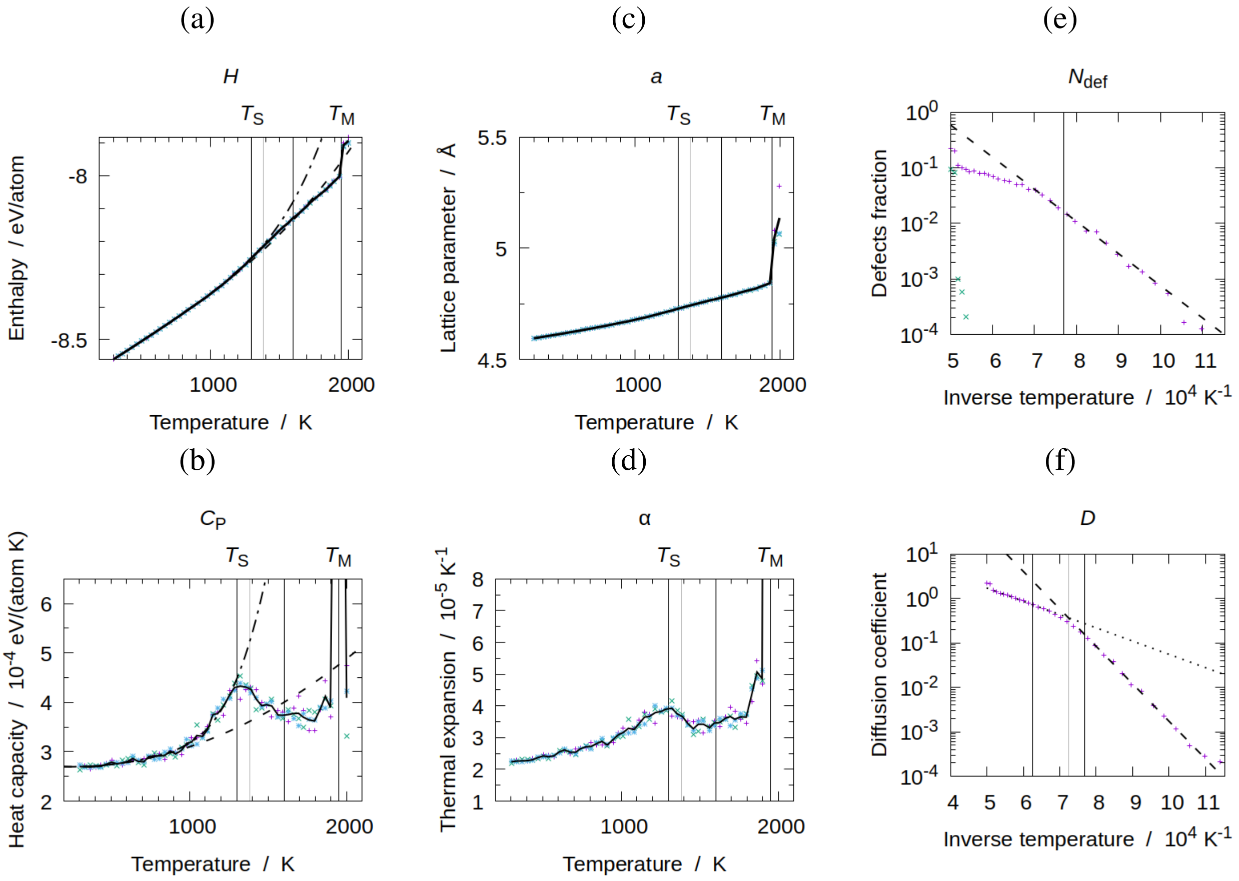


Figure S9: Thermodynamical properties from MD simulations of $Li_2O$ using the Oda potential.

## 1.10 $Li_2O$ – Pedone

| | |
|---|---|
| Superionic transition temperature: | $T_S = 1050$ K |
| Mechanical melting point: | $T_M = 1656$ K |
| Crossover temperature: | $T_C = 1125$ K |
| Frenkel pair formation enthalpy: | $H_f = 2.350$ eV |
| Diffusion activation energy in the crystal phase: | $E_a^c = 1.722$ eV |
| Diffusion activation energy in the superionic phase: | $E_a^s = 0.451$ eV |

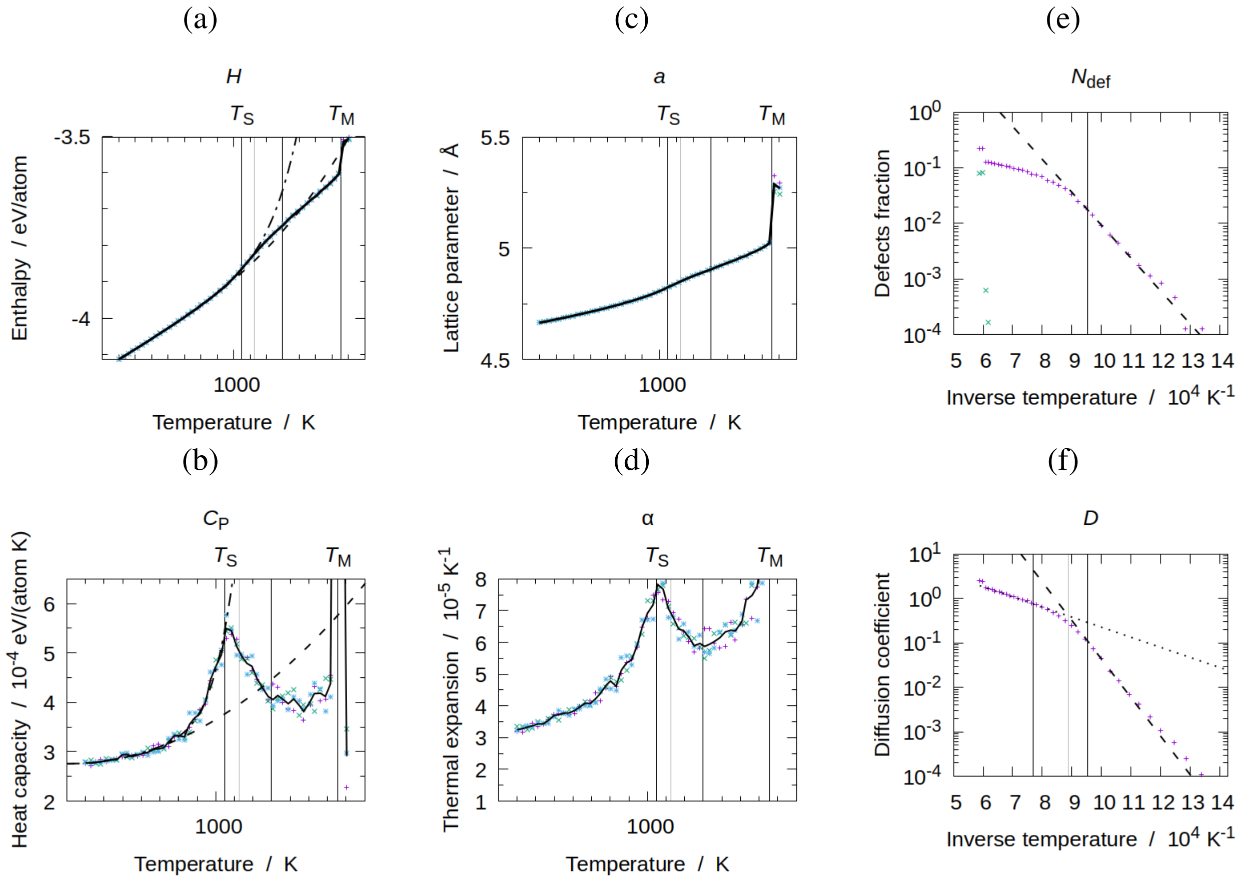


Figure S10: Thermodynamical properties from MD simulations of $Li_2O$ using the Pedone potential.

## 1.11 $\alpha$-$PbF_2$ – Catlow

| | |
|---|---|
| Superionic transition temperature: | $T_S$ = 1400 K |
| Mechanical melting point: | $T_M$ = 1952 K |
| Crossover temperature: | $T_C$ = 1490 K |
| Frenkel pair formation enthalpy: | $H_f$ = 2.934 eV |
| Diffusion activation energy in the crystal phase: | $E_a^c$ = 1.952 eV |
| Diffusion activation energy in the superionic phase: | $E_a^s$ = 0.805 eV |

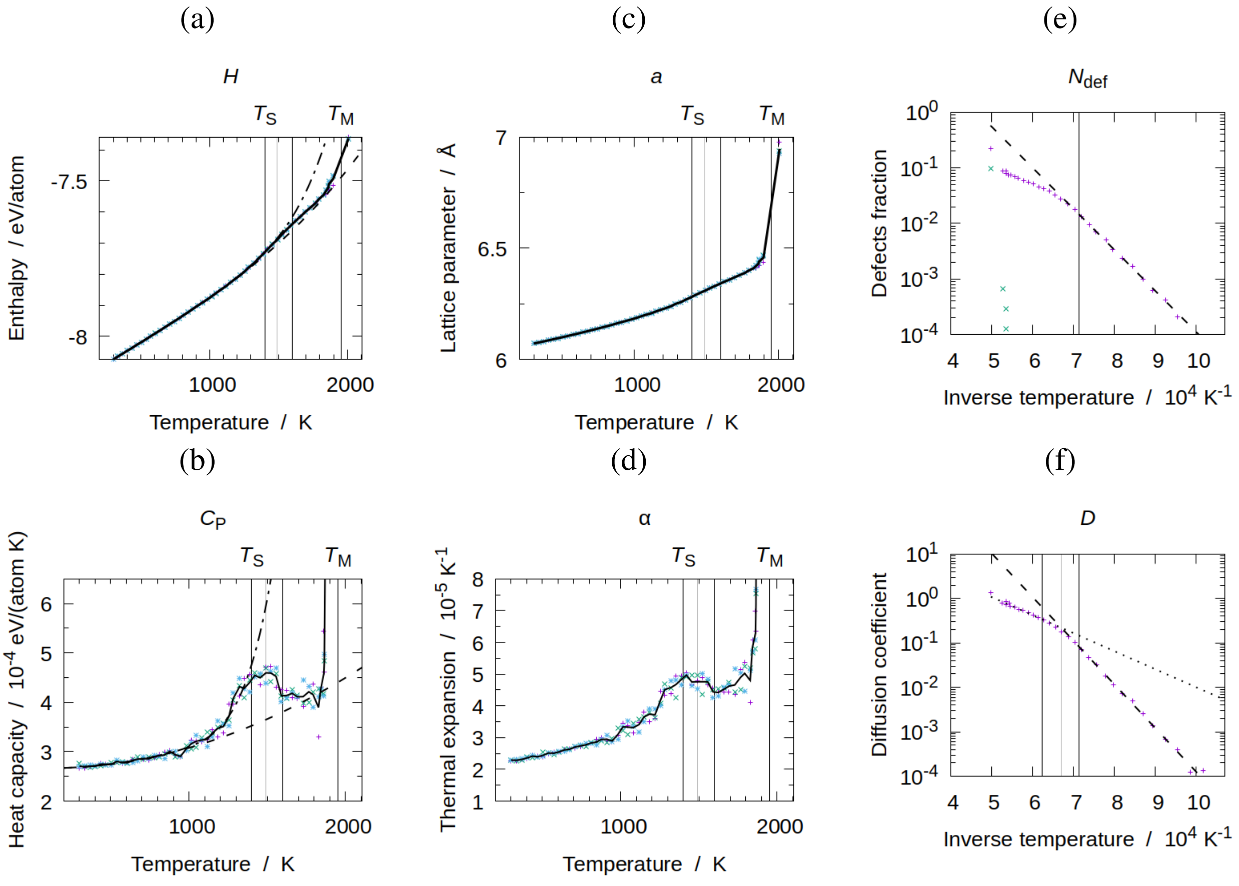


Figure S11: Thermodynamical properties from MD simulations of $\alpha$-$PbF_2$ using the Catlow potential.

## 1.12 $SrCl_2$ – Bendall

| | |
|---|---|
| Superionic transition temperature: | $T_S$ = 1200 K |
| Mechanical melting point: | $T_M$ = 1511 K |
| Crossover temperature: | $T_C$ = 1260 K |
| Frenkel pair formation enthalpy: | $H_f$ = 2.643 eV |
| Diffusion activation energy in the crystal phase: | $E_a^c$ = 1.885 eV |
| Diffusion activation energy in the superionic phase: | $E_a^s$ = 0.832 eV |

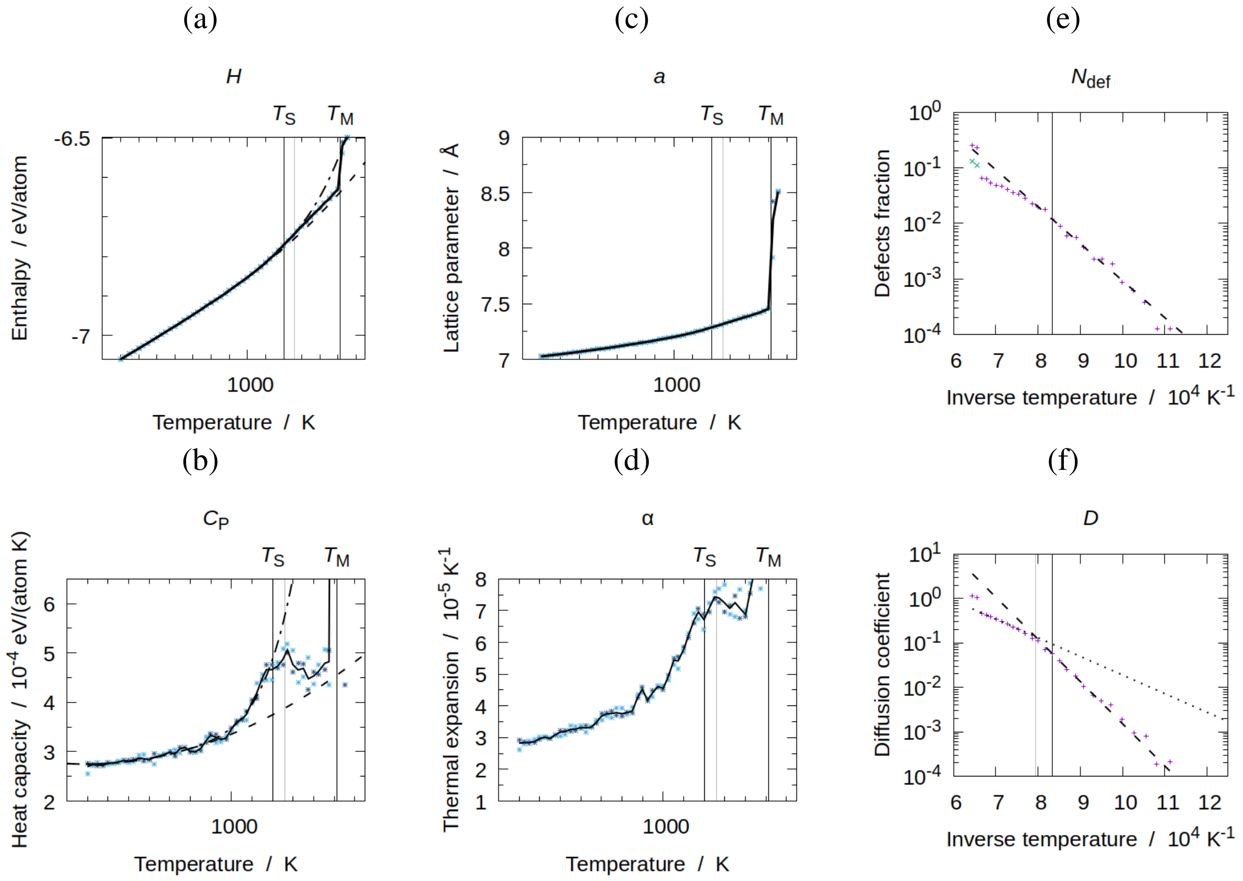


Figure S12: Thermodynamical properties from MD simulations of $SrCl_2$ using the Bendall potential.

## 1.13 $SrCl_2$ – Gillan

| | |
|---|---|
| Superionic transition temperature: | $T_S = 1050$ K |
| Mechanical melting point: | $T_M = 1288$ K |
| Crossover temperature: | $T_C = 1101$ K |
| Frenkel pair formation enthalpy: | $H_f = 1.839$ eV |
| Diffusion activation energy in the crystal phase: | $E_a^c = 1.111$ eV |
| Diffusion activation energy in the superionic phase: | $E_a^s = 0.713$ eV |

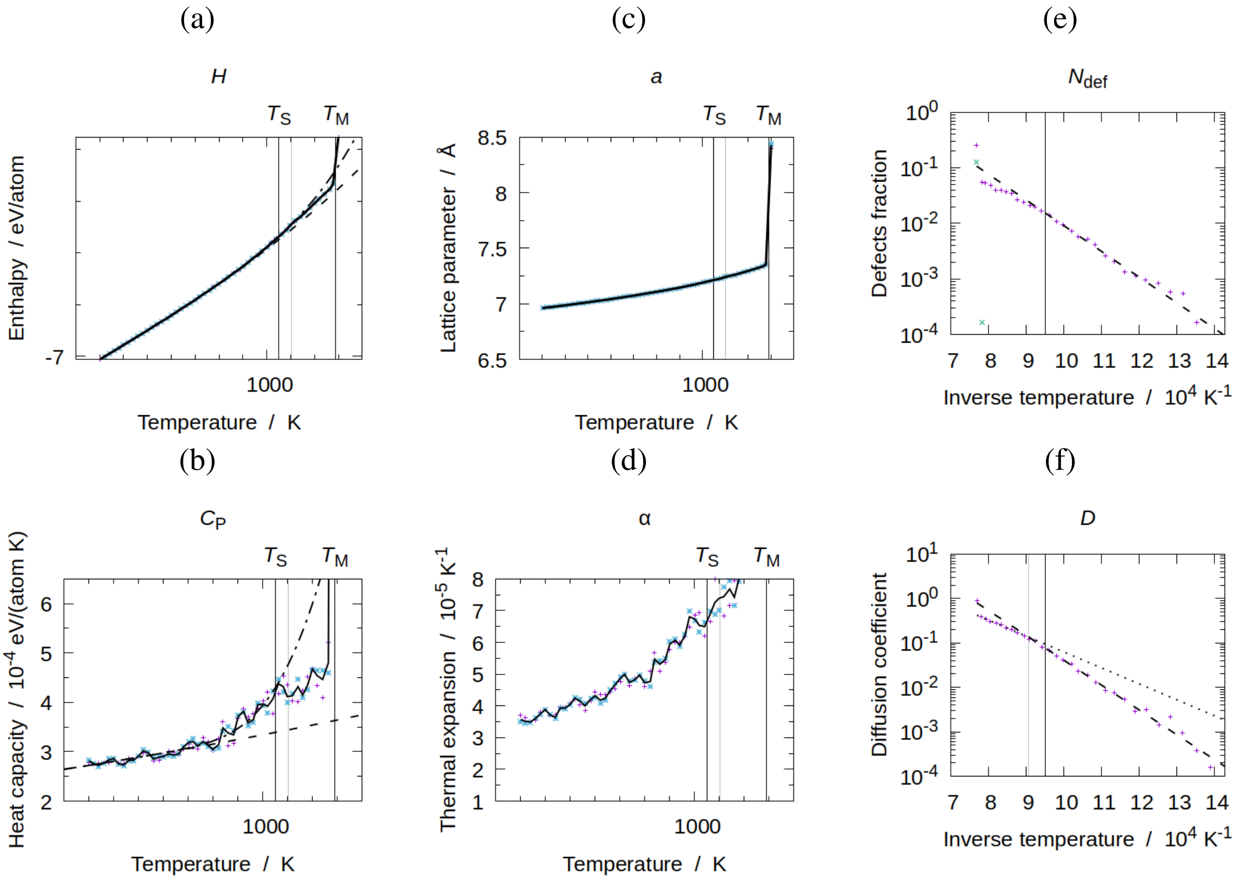


Figure S13: Thermodynamical properties from MD simulations of $SrCl_2$ using the Gillan potential.

## 1.14 $SrF_2$ – Bingham

| | |
|---|---|
| Superionic transition temperature: | $T_S$ = 1200 K |
| Mechanical melting point: | $T_M$ = 1812 K |
| Crossover temperature: | $T_C$ = 1349 K |
| Frenkel pair formation enthalpy: | $H_f$ = 2.372 eV |
| Diffusion activation energy in the crystal phase: | $E_a^c$ = 1.509 eV |
| Diffusion activation energy in the superionic phase: | $E_a^s$ = 0.751 eV |

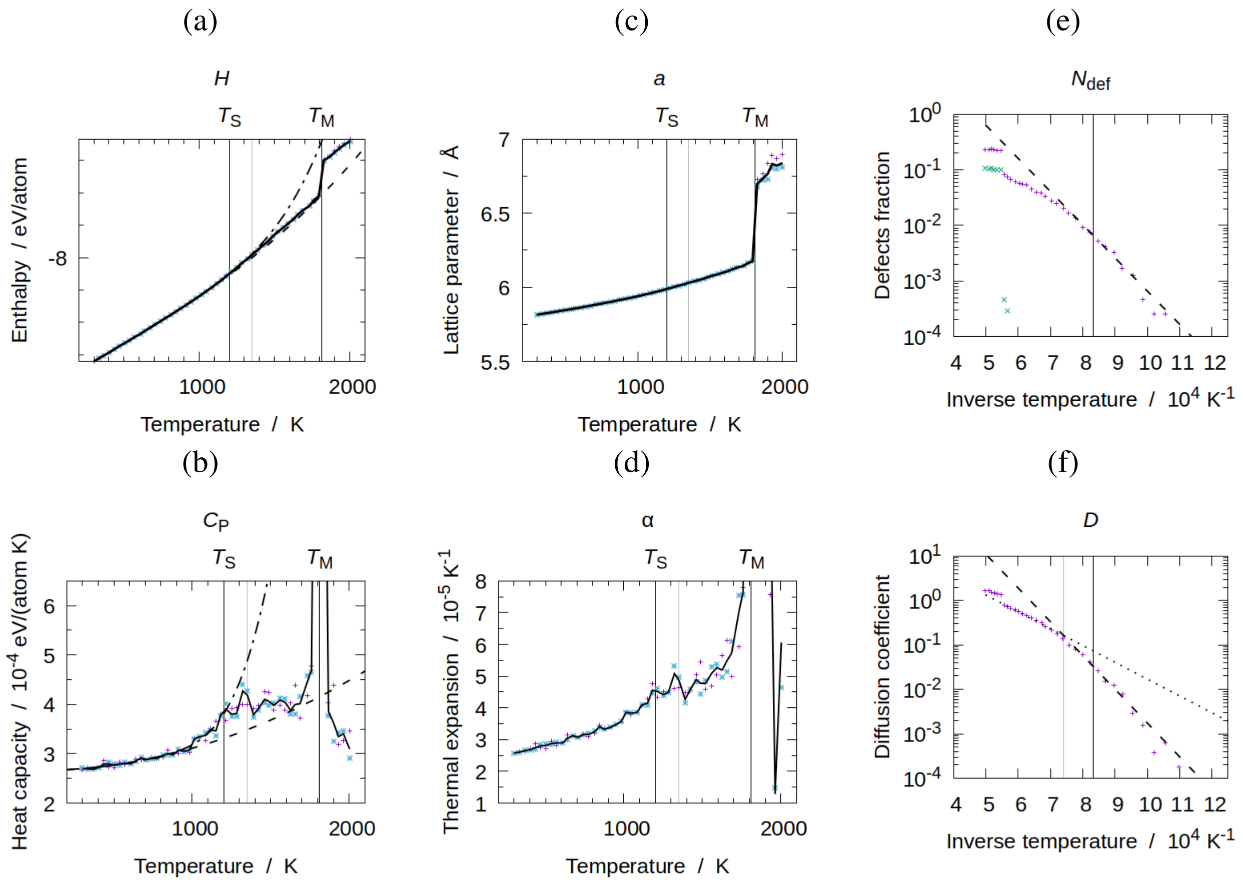


Figure S14: Thermodynamical properties from MD simulations of $SrF_2$ using the Bingham potential.

## 1.15 $SrF_2$ – Catlow

| | |
|---|---|
| Superionic transition temperature: | $T_S$ = 1630 K |
| Mechanical melting point: | $T_M$ = 2531 K |
| Crossover temperature: | $T_C$ = 1715 K |
| Frenkel pair formation enthalpy: | $H_f$ = 3.655 eV |
| Diffusion activation energy in the crystal phase: | $E_a^c$ = 2.755 eV |
| Diffusion activation energy in the superionic phase: | $E_a^s$ = 0.629 eV |

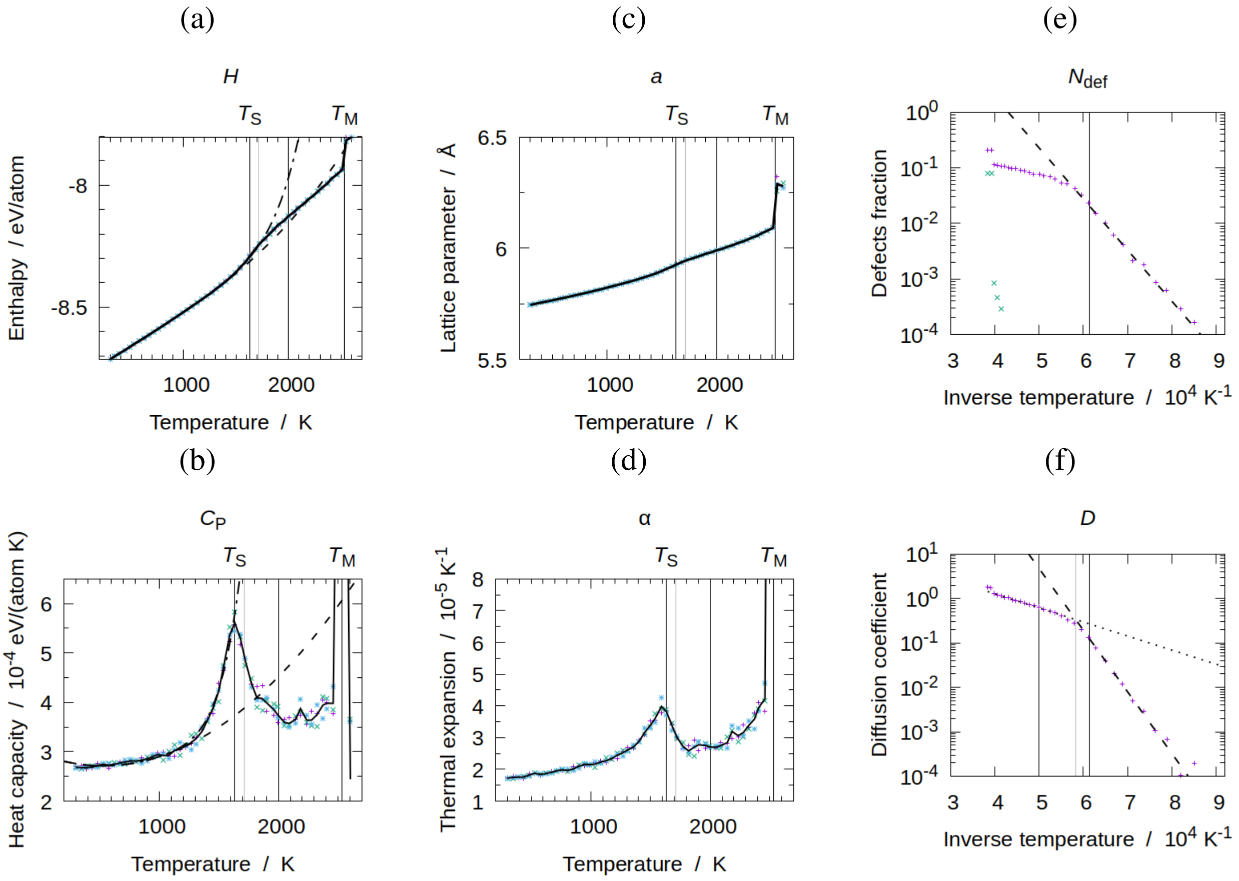


Figure S15: Thermodynamical properties from MD simulations of $SrF_2$ using the Catlow potential.

## 1.16 $SrF_2$ – Cazorla

| | |
|---|---|
| Superionic transition temperature: | $T_S = 1500$ K |
| Mechanical melting point: | $T_M = 2079$ K |
| Crossover temperature: | $T_C = 1572$ K |
| Frenkel pair formation enthalpy: | $H_f = 3.867$ eV |
| Diffusion activation energy in the crystal phase: | $E_a^c = 2.408$ eV |
| Diffusion activation energy in the superionic phase: | $E_a^s = 0.785$ eV |

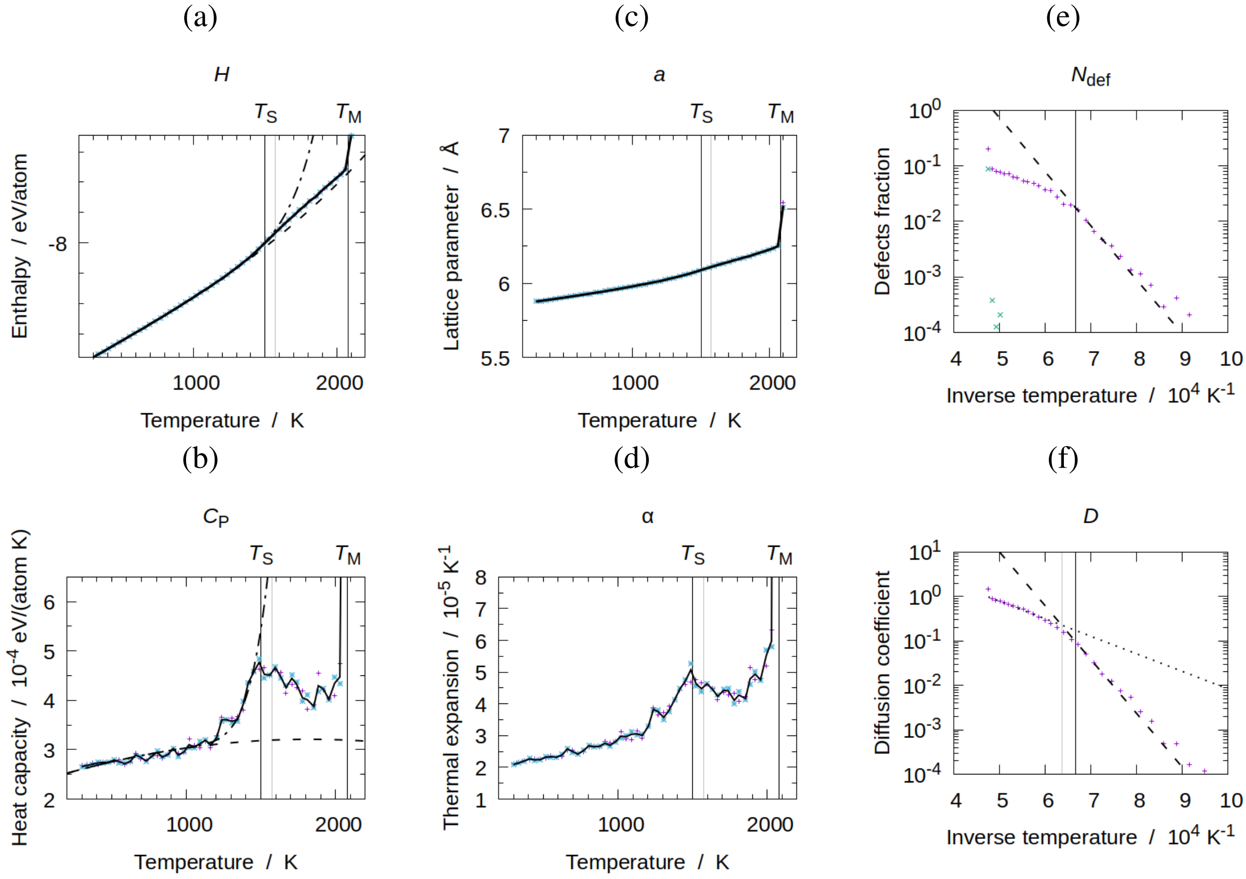


Figure S16: Thermodynamical properties from MD simulations of $SrF_2$ using the Cazorla potential.

## 1.17 $UO_2$ – CRG

| | |
|---|---|
| Superionic transition temperature: | $T_S = 2550$ K |
| Mechanical melting point: | $T_M = 3437$ K |
| Crossover temperature: | $T_C = 2607$ K |
| Frenkel pair formation enthalpy: | $H_f = 8.583$ eV |
| Diffusion activation energy in the crystal phase: | $E_a^c = 5.330$ eV |
| Diffusion activation energy in the superionic phase: | $E_a^s = 1.504$ eV |

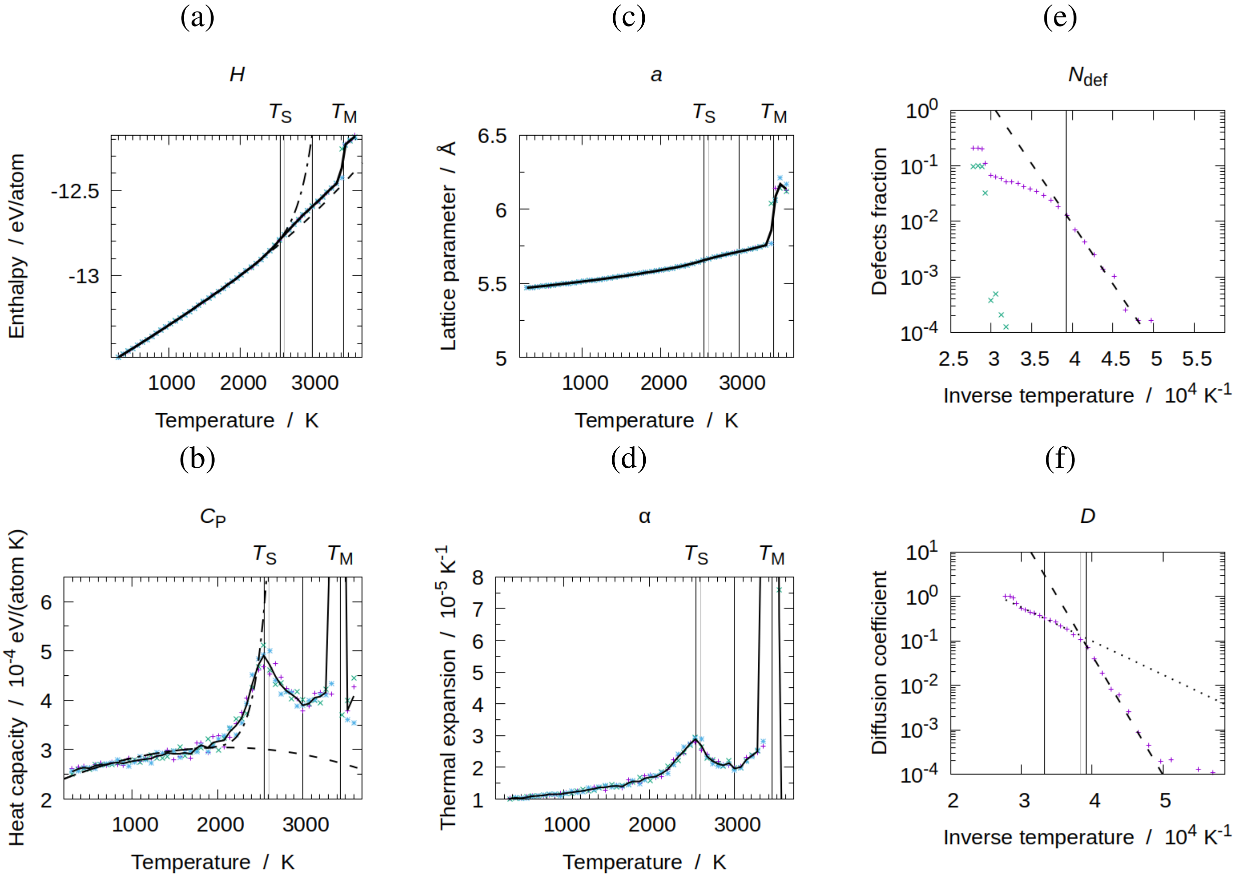


Figure S17: Thermodynamical properties from MD simulations of $UO_2$ using the CRG potential.

## 1.18 $UO_2$ – Morelon

| | |
|---|---|
| Superionic transition temperature: | $T_\mathrm{S}$ = 2600 K |
| Mechanical melting point: | $T_\mathrm{M}$ = 4094 K |
| Crossover temperature: | $T_\mathrm{C}$ = 2853 K |
| Frenkel pair formation enthalpy: | $H_\mathrm{f}$ = 4.354 eV |
| Diffusion activation energy in the crystal phase: | $E_\mathrm{a}^\mathrm{c}$ = 2.725 eV |
| Diffusion activation energy in the superionic phase: | $E_\mathrm{a}^\mathrm{s}$ = 1.363 eV |

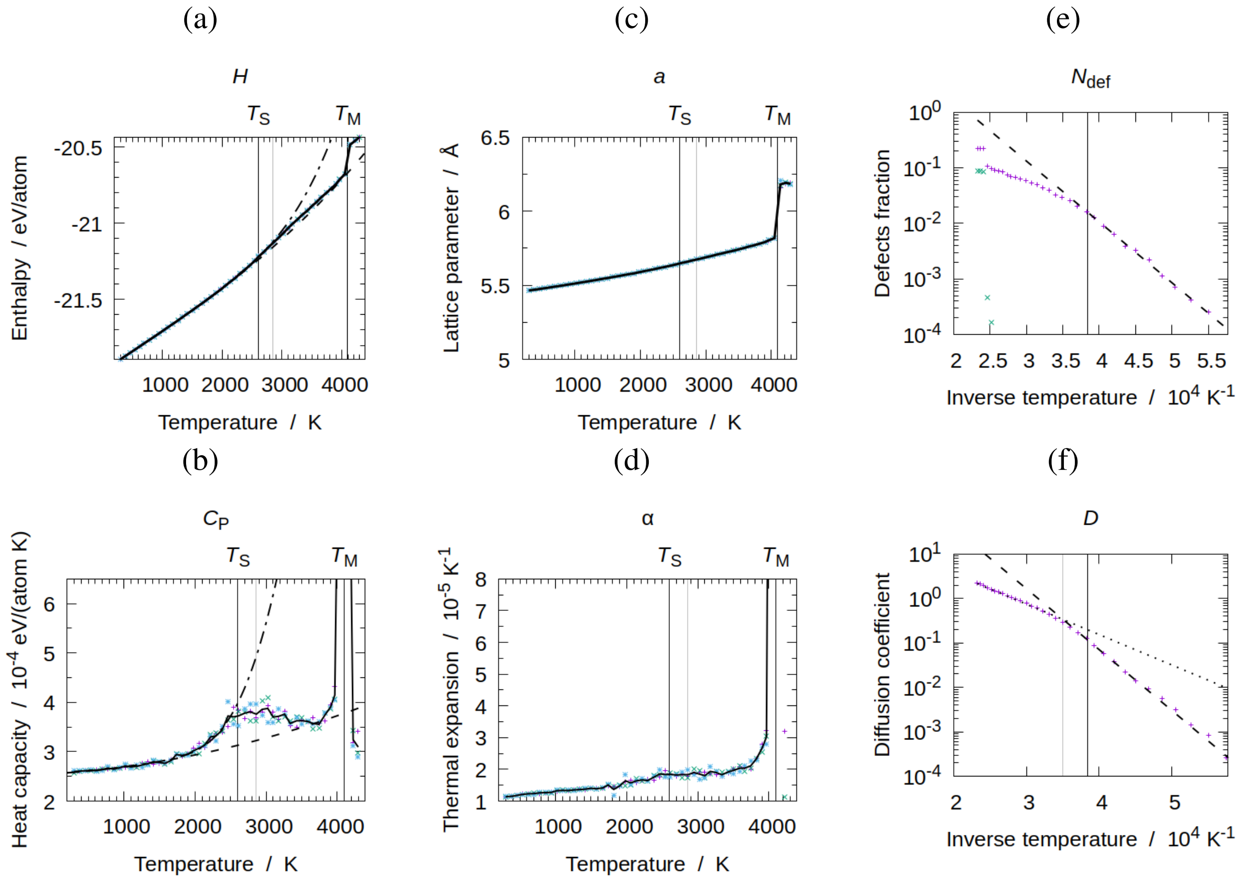


Figure S18: Thermodynamical properties from MD simulations of $UO_2$ using the Morelon potential.

## 2 SIMULATED POWDER XRD PATTERNS

The main article contains a discussion of thermodynamical and defects properties of fluorite compounds simulated with empirical potentials. For the sake of conciseness, only one potential is shown in the figures 3 and 4. This document contains the equivalent figures for the other potentials. The reader is referred to the main article for references and a discussion of these figures.

### 2.1 $BaF_2$ — Catlow

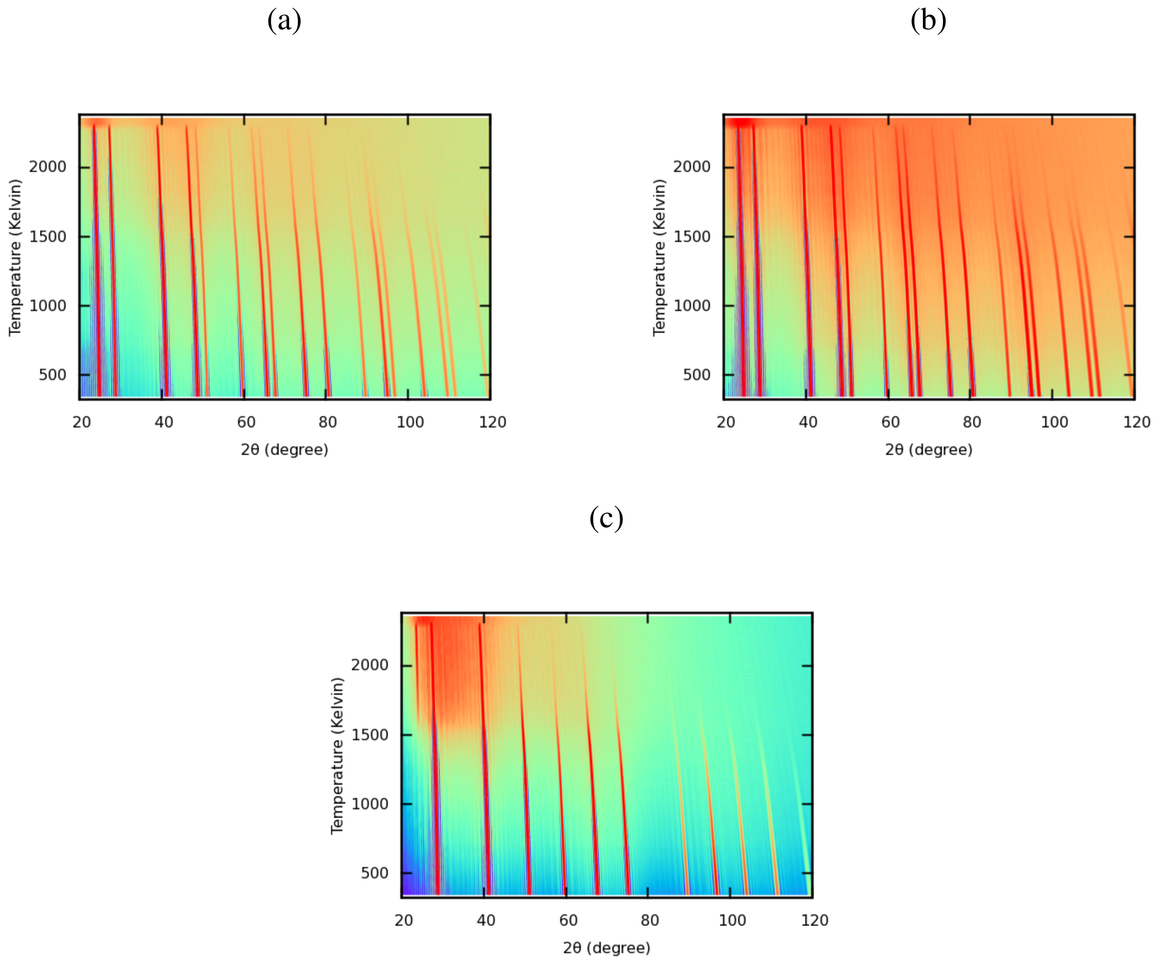


Figure S19: Full-crystal simulated XRD patterns from MD simulations of $BaF_2$ using the Catlow potential: (a) full structure; (b) Ba sublattice only; (c) F sublattice only.

## 2.2 $BaF_2$ — Sayle

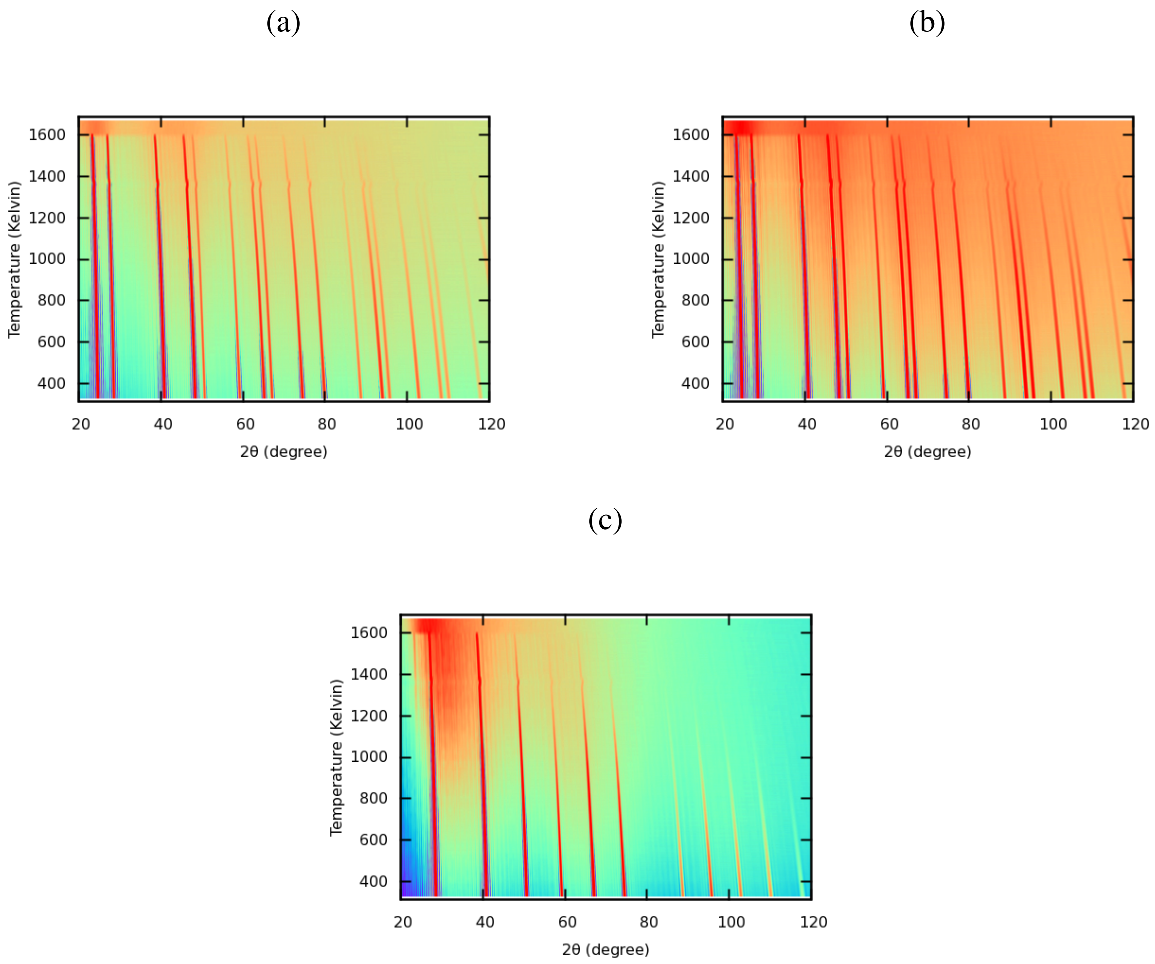


Figure S20: Full-crystal simulated XRD patterns from MD simulations of $BaF_2$ using the Sayle potential: (a) full structure; (b) Ba sublattice only; (c) F sublattice only.

## 2.3 $CaF_2$ — Bingham

(a)

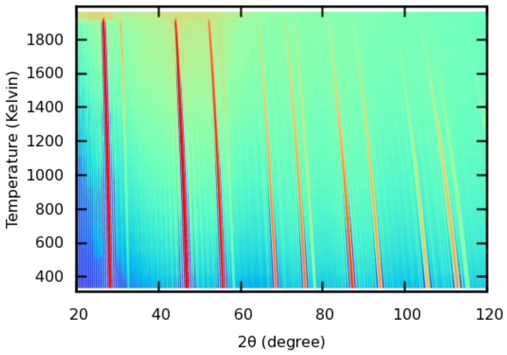


(b)

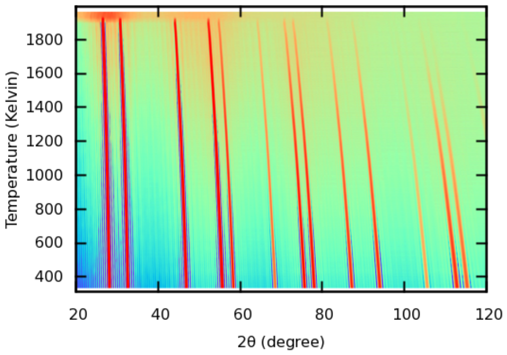


(c)

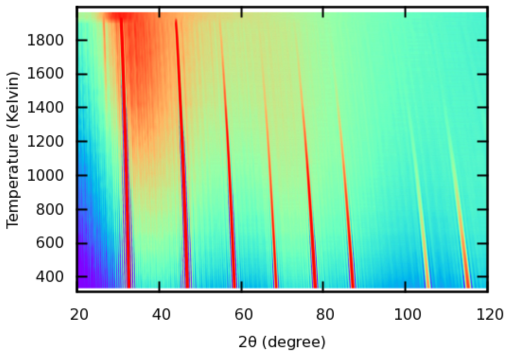


Figure S21: Full-crystal simulated XRD patterns from MD simulations of $CaF_2$ using the Bingham potential: (a) full structure; (b) Ca sublattice only; (c) F sublattice only.

## 2.4 $CaF_2$ — Catlow

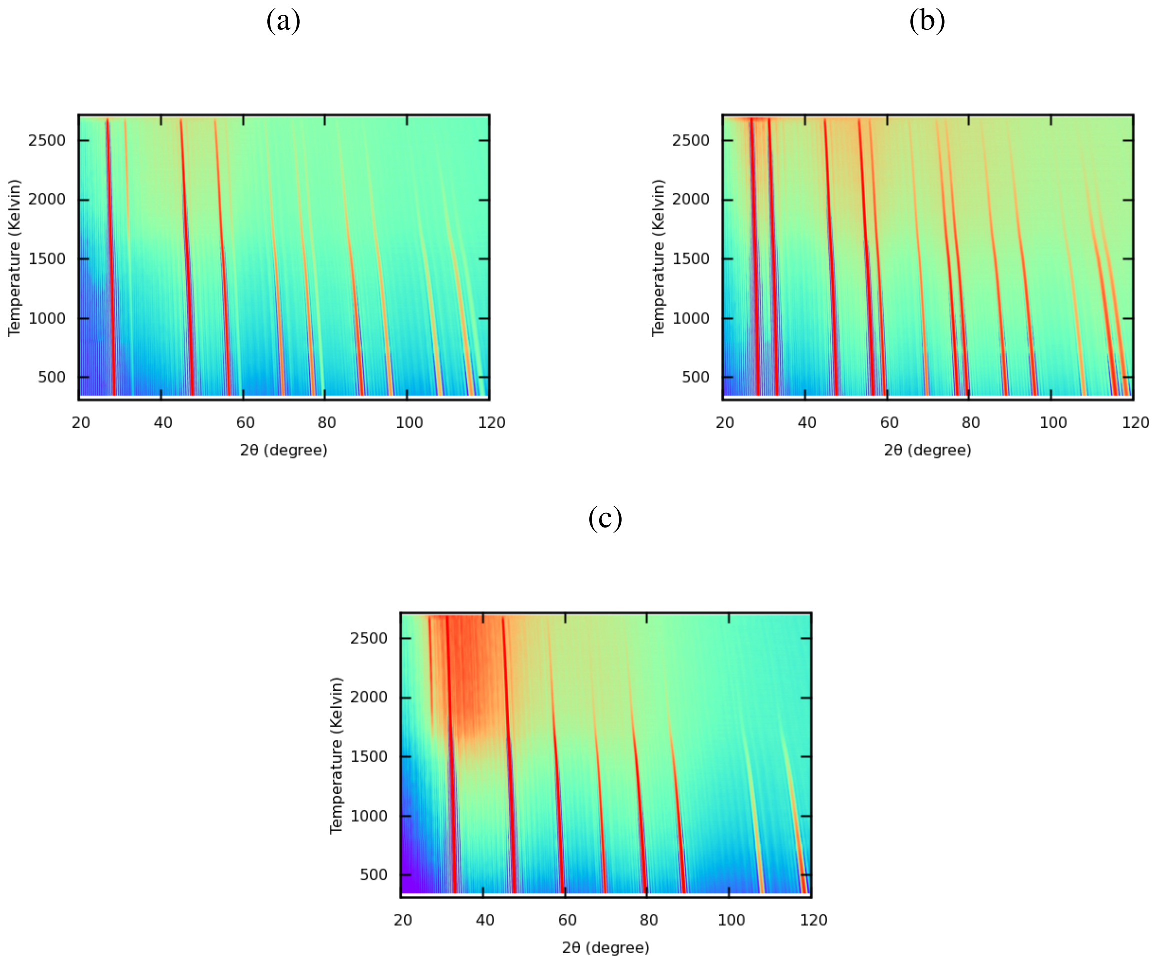


Figure S22: Full-crystal simulated XRD patterns from MD simulations of $CaF_2$ using the Catlow potential: (a) full structure; (b) Ca sublattice only; (c) F sublattice only.

## 2.5 $CaF_2$ — Evangelakis

(a) (b)

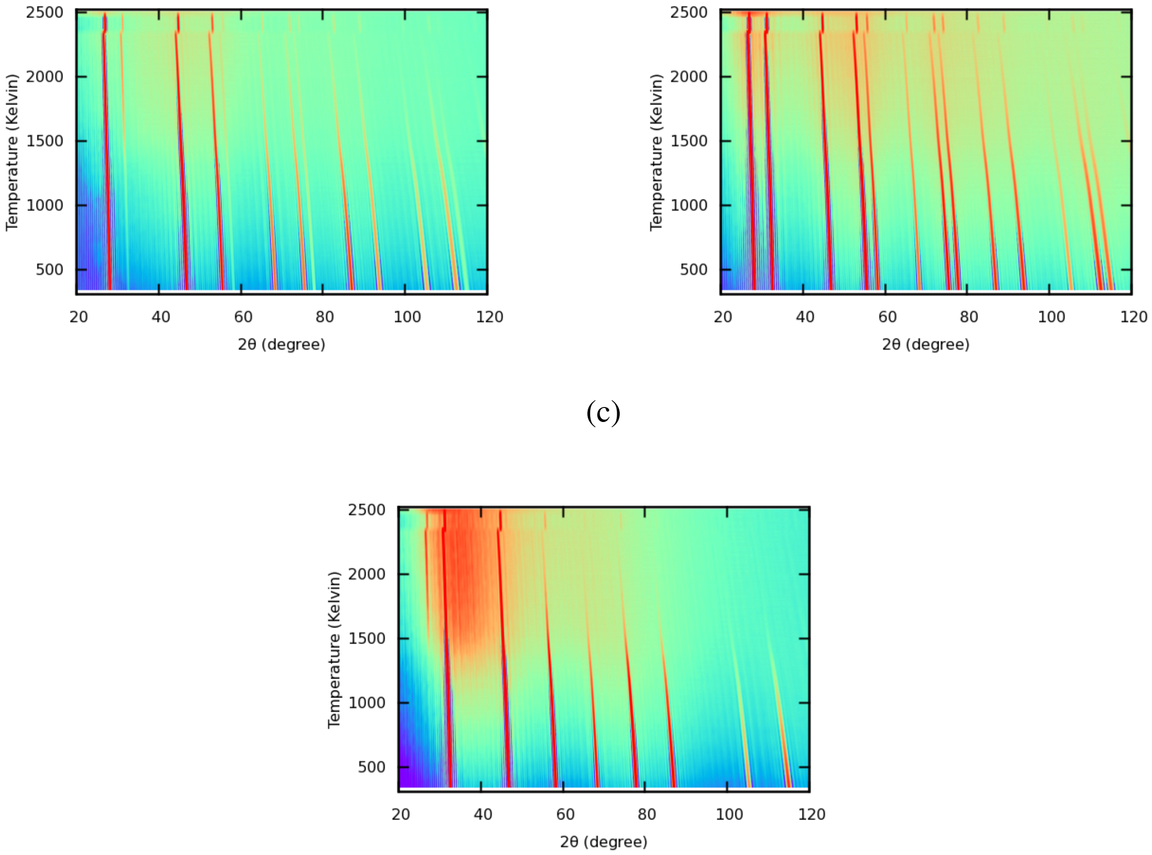


Figure S23: Full-crystal simulated XRD patterns from MD simulations of $CaF_2$ using the Evangelakis potential: (a) full structure; (b) Ca sublattice only; (c) F sublattice only.

## 2.6 $CaF_2$ — Sayle

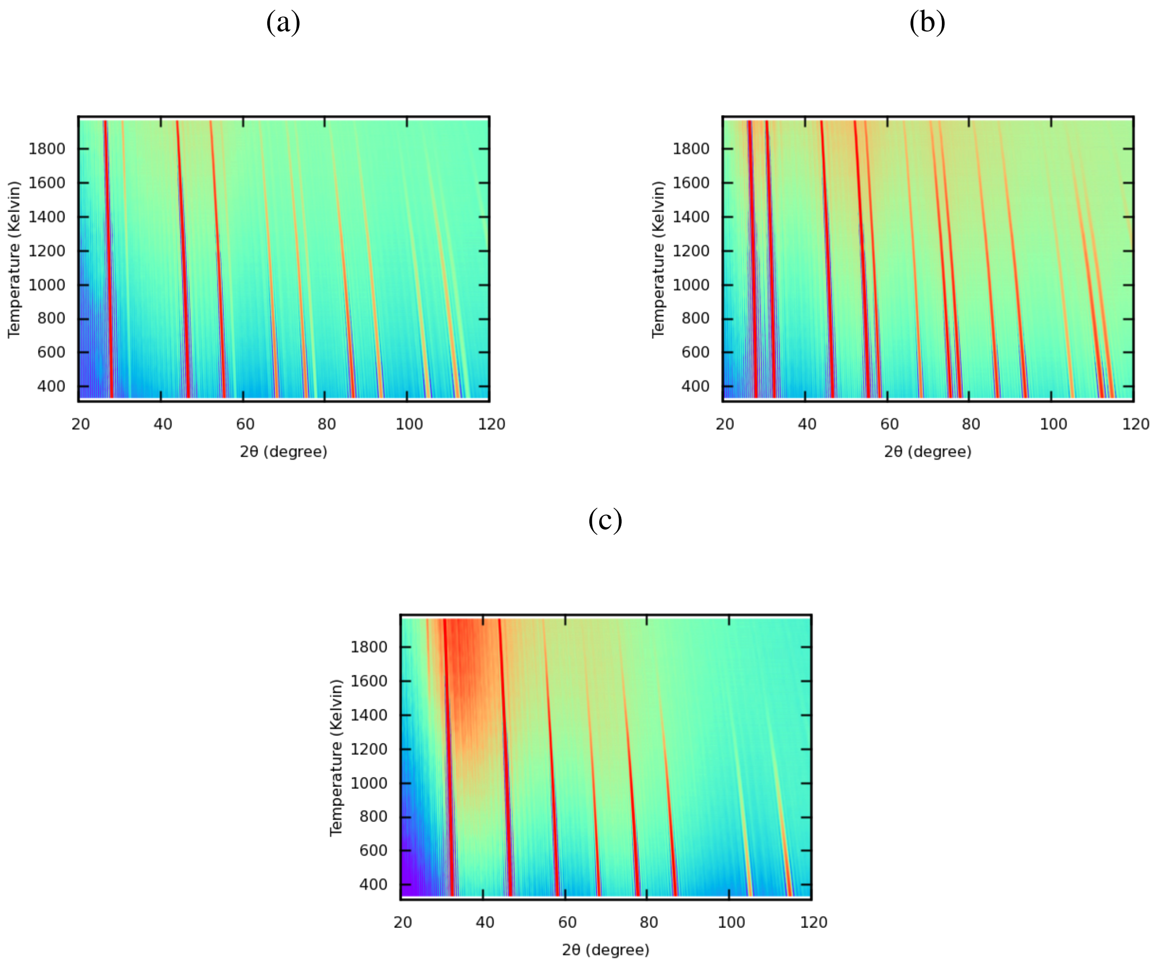


Figure S24: Full-crystal simulated XRD patterns from MD simulations of $CaF_2$ using the Sayle potential: (a) full structure; (b) Ca sublattice only; (c) F sublattice only.

## 2.7 $Li_2O$ — Asahi

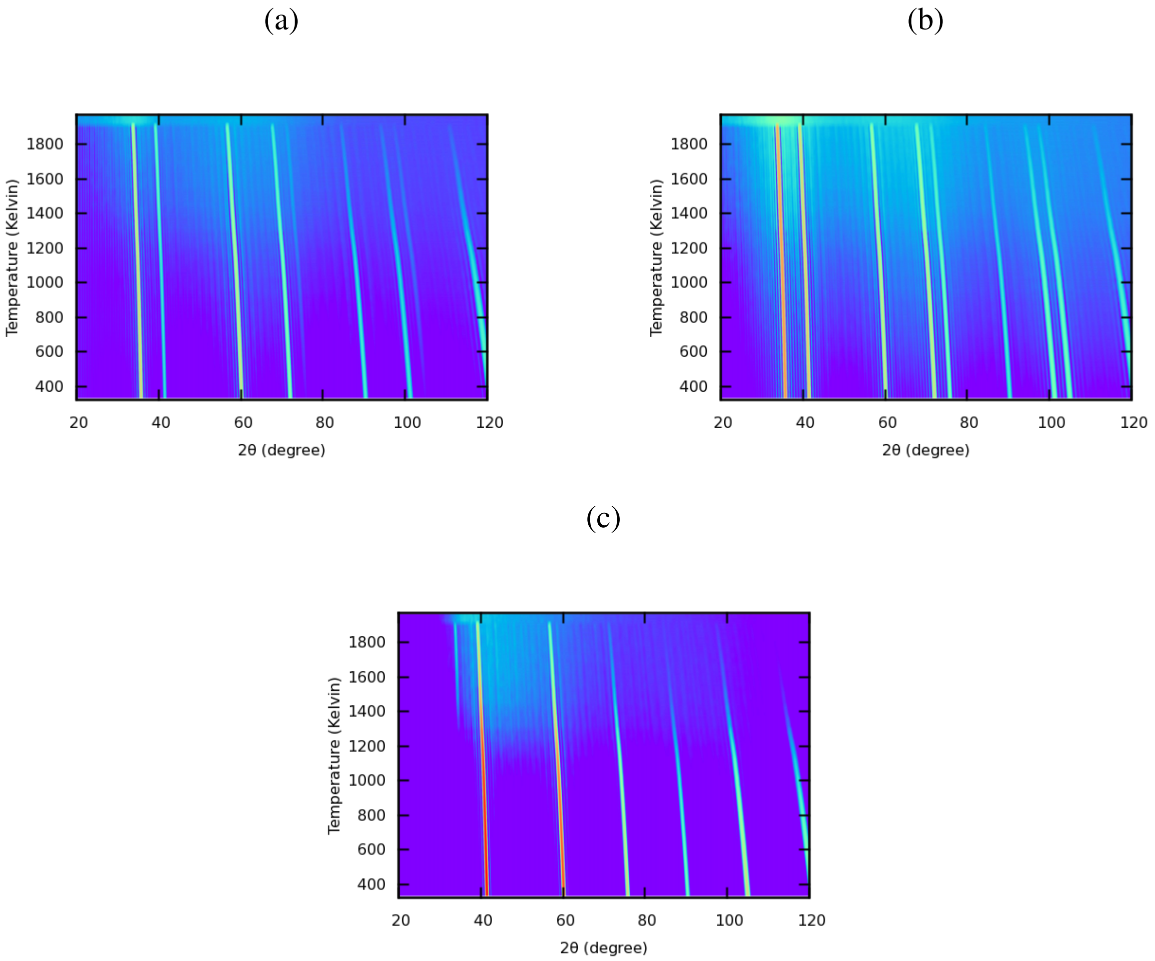


Figure S25: Full-crystal simulated XRD patterns from MD simulations of $Li_2O$ using the Asahi potential: (a) full structure; (b) O sublattice only; (c) Li sublattice only.

## 2.8 $Li_2O$ — Oda

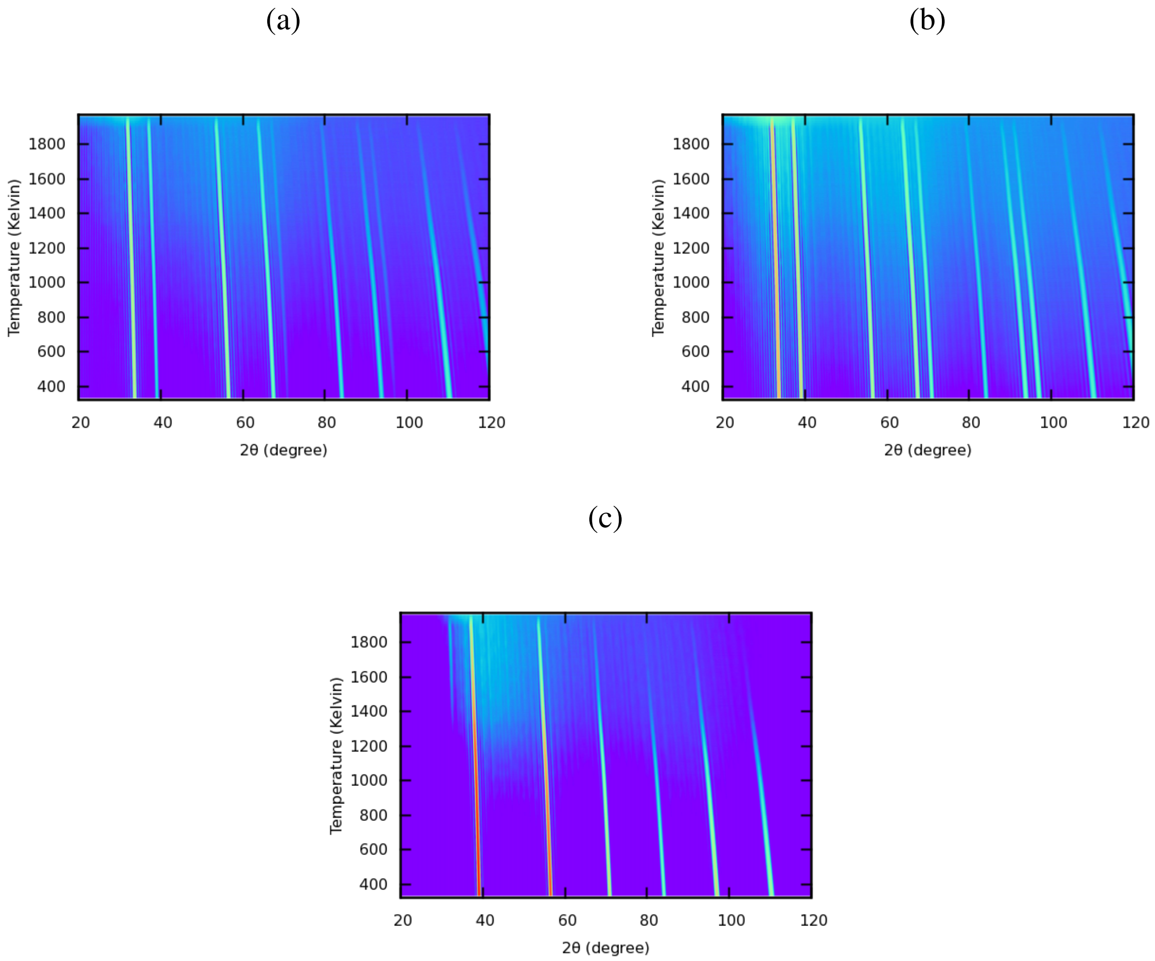


Figure S26: Full-crystal simulated XRD patterns from MD simulations of $Li_2O$ using the Oda potential: (a) full structure; (b) O sublattice only; (c) Li sublattice only.

## 2.9 $Li_2O$ — Pedone

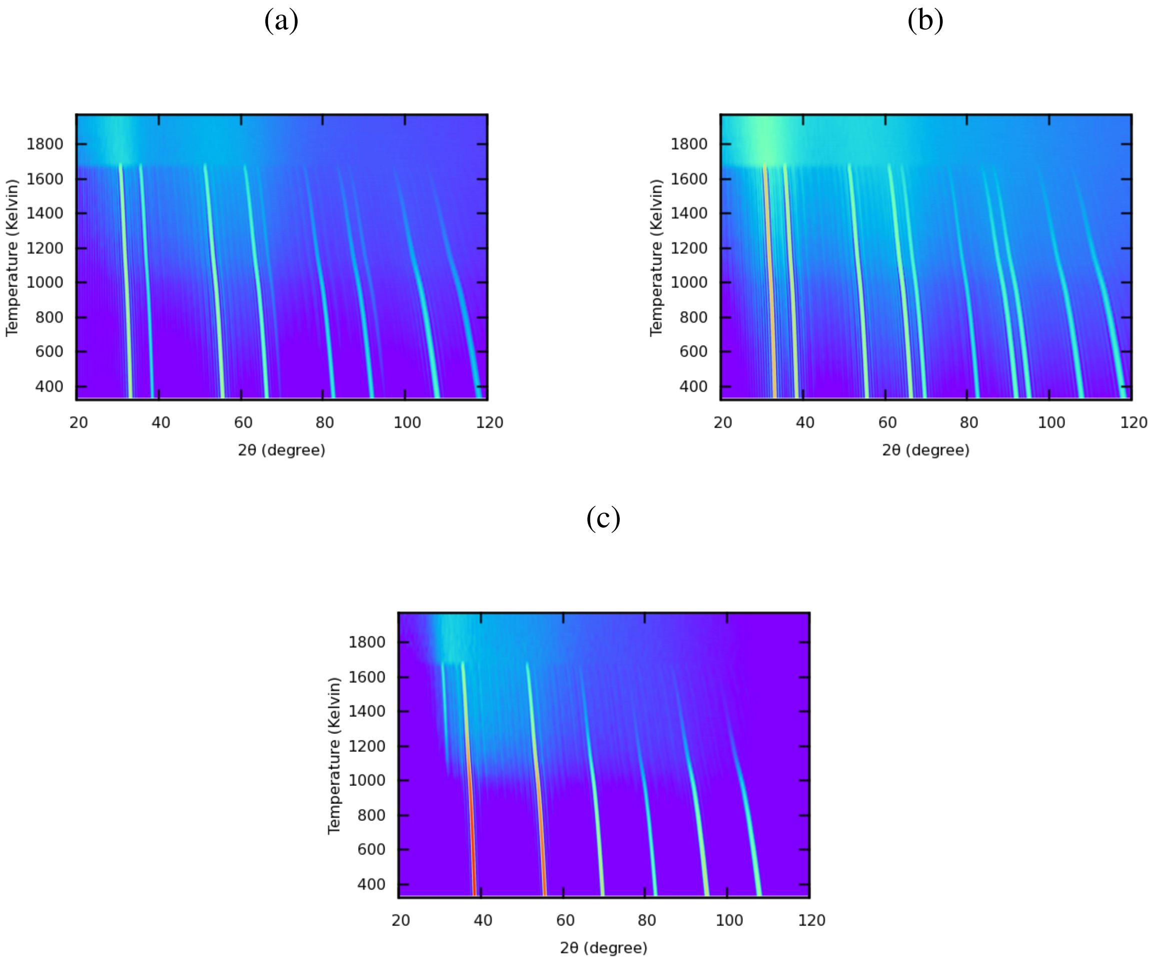


Figure S27: Full-crystal simulated XRD patterns from MD simulations of $Li_2O$ using the Pedone potential: (a) full structure; (b) O sublattice only; (c) Li sublattice only.

## 2.10 $\beta$-$PbF_2$ — Catlow

(a)

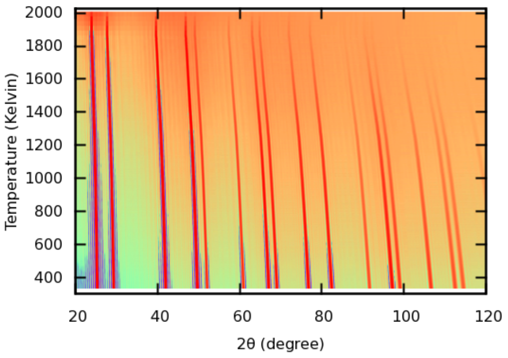


(b)

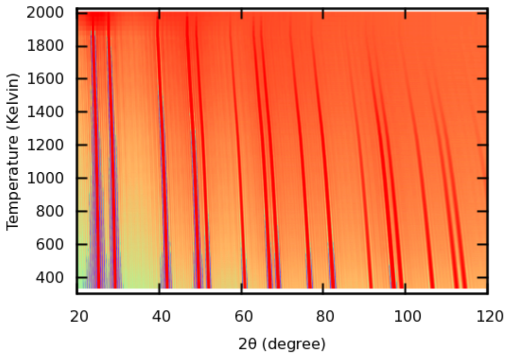


(c)

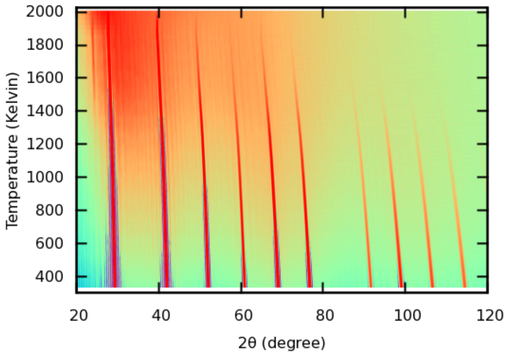


Figure S28: Full-crystal simulated XRD patterns from MD simulations of $\beta$-$PbF_2$ using the Catlow potential: (a) full structure; (b) Pb sublattice only; (c) F sublattice only.

## 2.11 $SrCl_2$ — Bendall

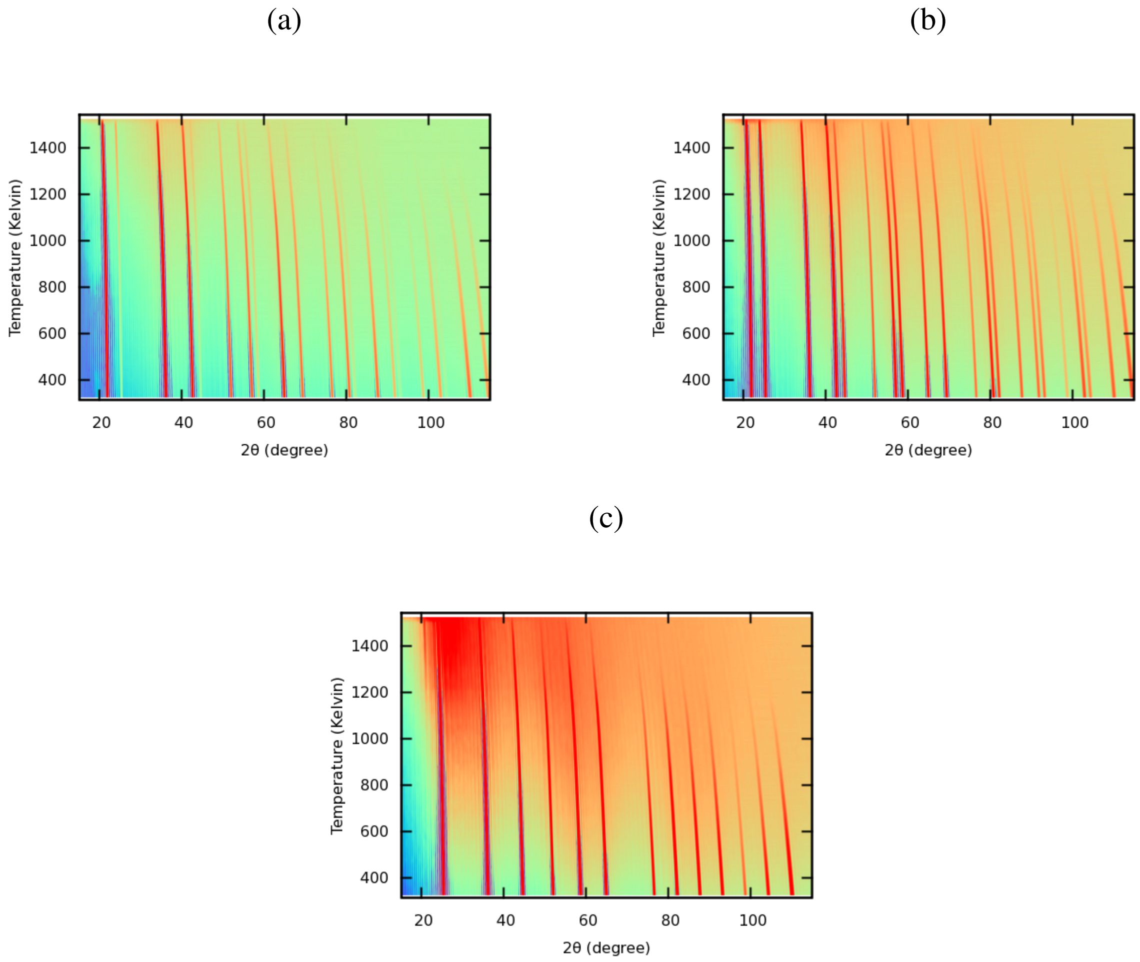


Figure S29: Full-crystal simulated XRD patterns from MD simulations of $SrCl_2$ using the Bendall potential: (a) full structure; (b) Sr sublattice only; (c) Cl sublattice only.

## 2.12 $SrCl_2$ — Gillan

(a)

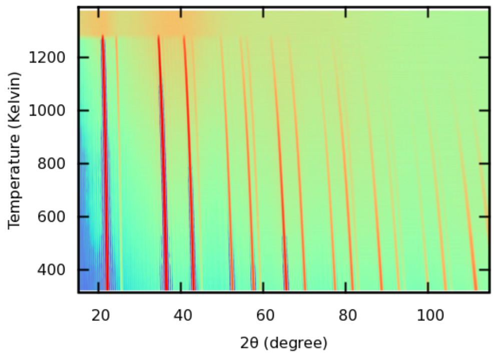


(b)

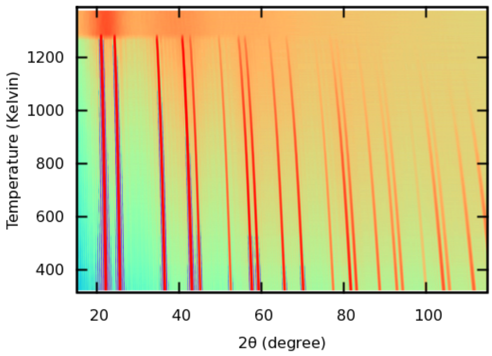


(c)

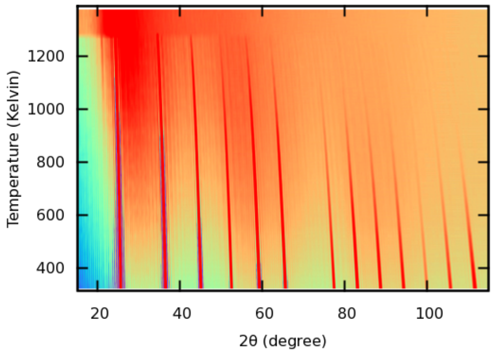


Figure S30: Full-crystal simulated XRD patterns from MD simulations of $SrCl_2$ using the Gillan potential: (a) full structure; (b) Sr sublattice only; (c) Cl sublattice only.

## 2.13 $SrF_2$ — Bingham

(a)

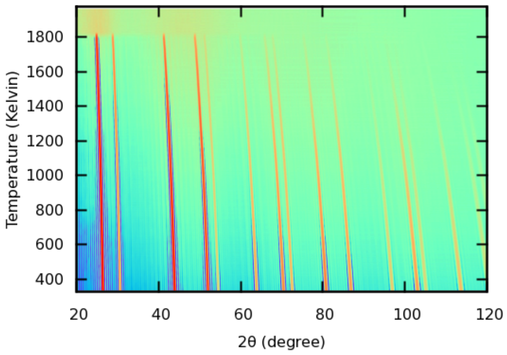


(b)

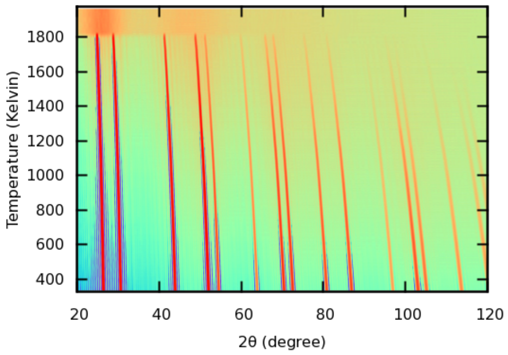


(c)

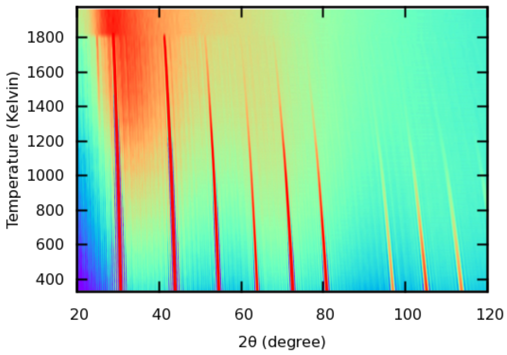


Figure S31: Full-crystal simulated XRD patterns from MD simulations of $SrF_2$ using the Bingham potential: (a) full structure; (b) Sr sublattice only; (c) F sublattice only.

## 2.14 $SrF_2$ — Catlow

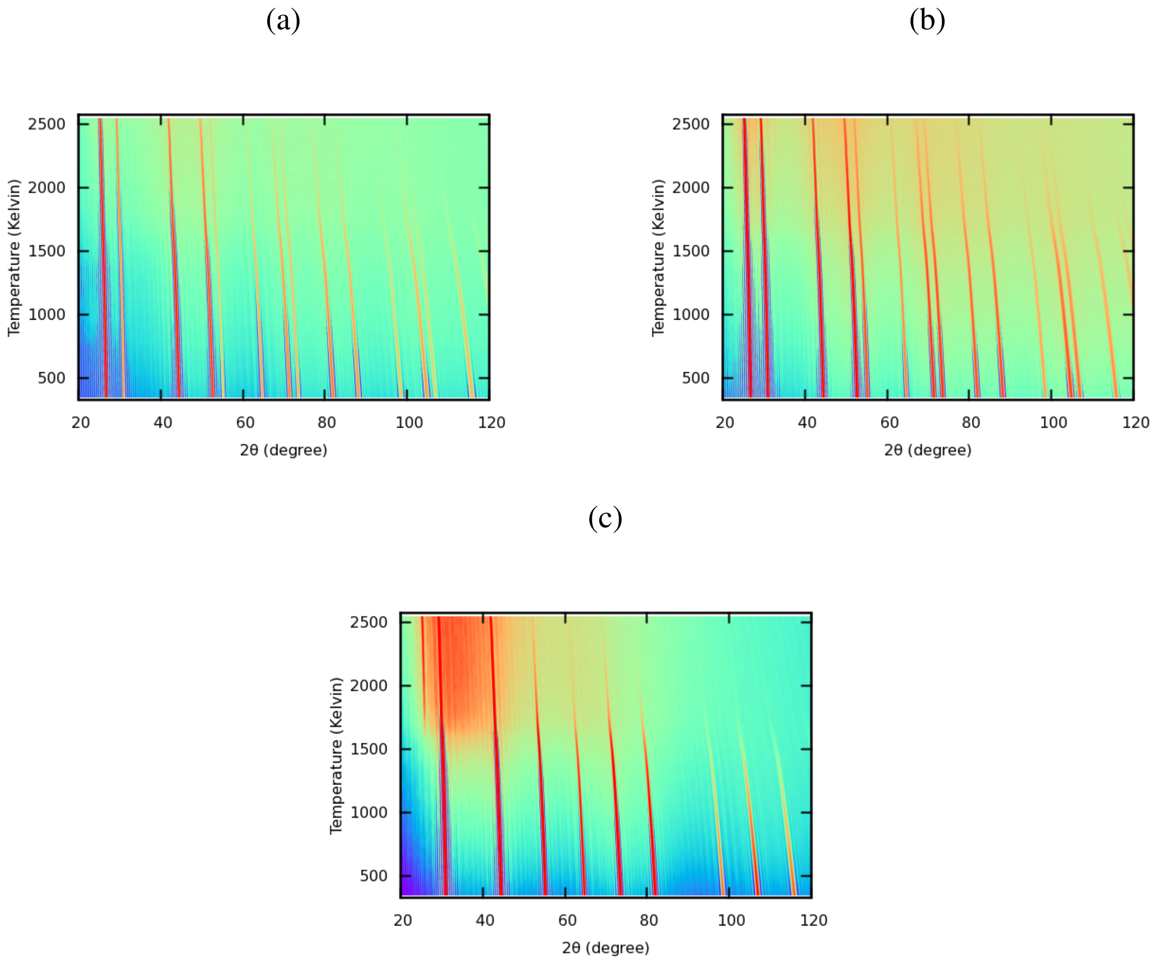


Figure S32: Full-crystal simulated XRD patterns from MD simulations of $SrF_2$ using the Catlow potential: (a) full structure; (b) Sr sublattice only; (c) F sublattice only.

## 2.15 $SrF_2$ — Cazorla

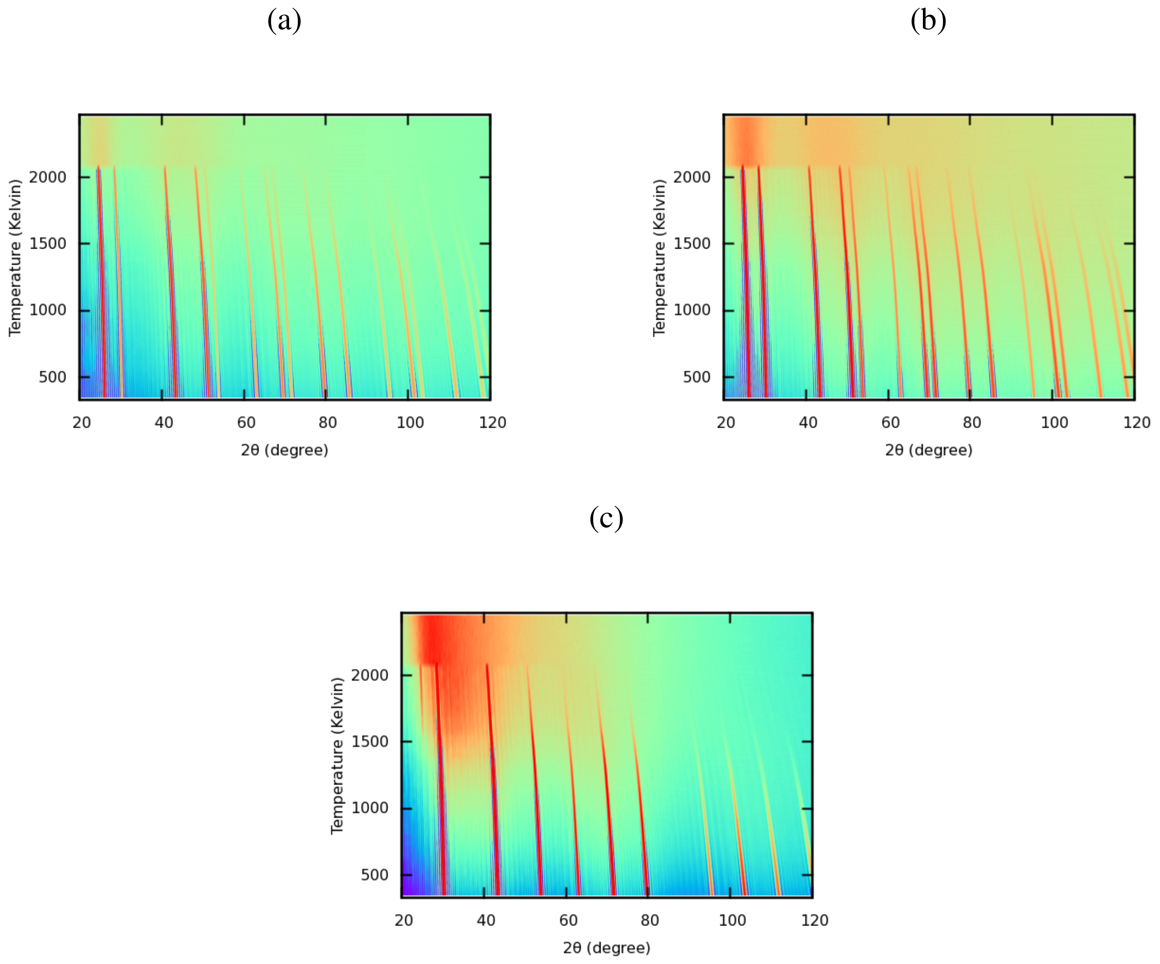


Figure S33: Full-crystal simulated XRD patterns from MD simulations of $SrF_2$ using the Cazorla potential: (a) full structure; (b) Sr sublattice only; (c) F sublattice only.

## 2.16 $UO_2$ — Cooper

(a)

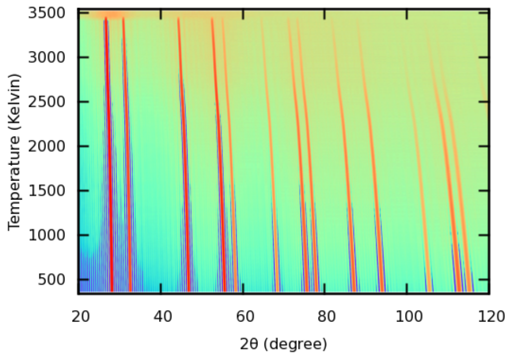


(b)

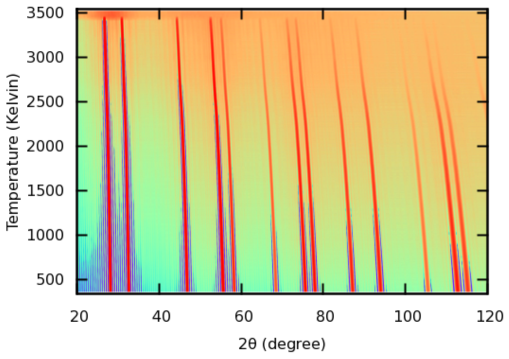


(c)

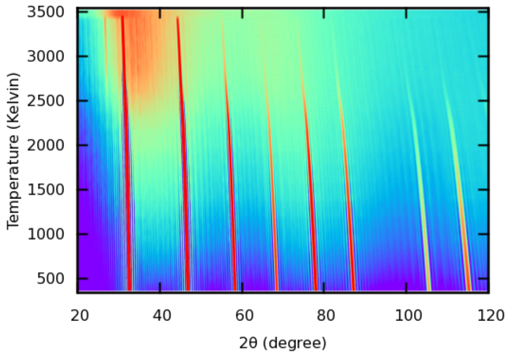


Figure S34: Full-crystal simulated XRD patterns from MD simulations of $UO_2$ using the Cooper potential: (a) full structure; (b) U sublattice only; (c) O sublattice only.

## 2.17 $UO_2$ — Morelon

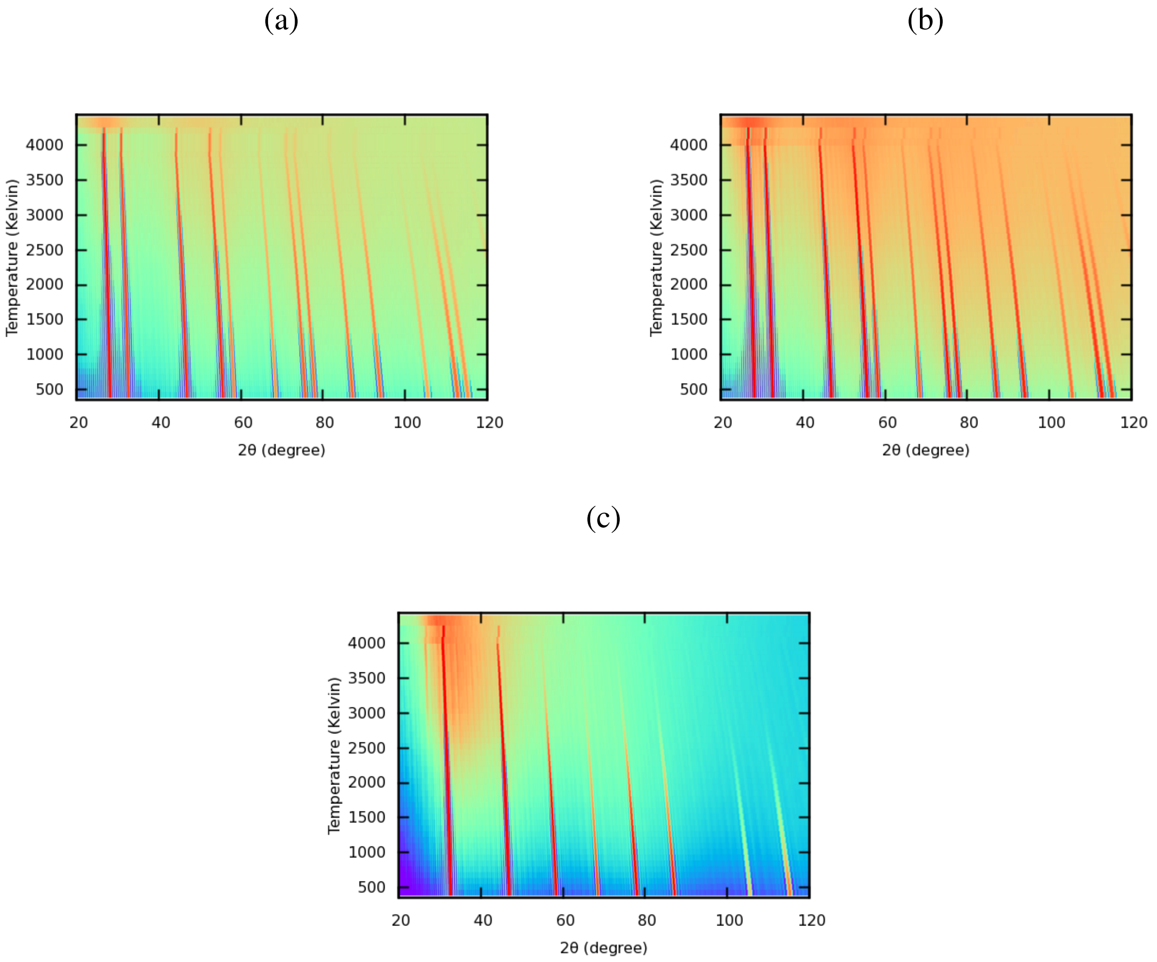


Figure S35: Full-crystal simulated XRD patterns from MD simulations of $UO_2$ using the Morelon potential: (a) full structure; (b) U sublattice only; (c) O sublattice only.
